# Supplementary material for: An ERAD‐independent role for rhomboid pseudoprotease Dfm1 in mediating sphingolipid homeostasis
Source: EMBO J. 2022 Nov 9;42(4):e112275. doi: 10.15252/embj.2022112275 (PMC9929635; doi:10.15252/embj.2022112275)
Supplement: Supplementary file 1 — Appendix S1 [file EMBJ-42-e112275-s001.pdf]

Appendix Tables S1-S3 for An ERAD-independent role for rhomboid pseudoprotease Dfm1 in mediating sphingolipid homeostasis

Appendix Table S1-related to Methods: Plasmids used in this study, pg. 2-3

Appendix Table S2-related to Methods: Yeast strains used in this study, pg. 4-8

Appendix Table-S3-related to Methods: Reagents used in this study, pg. 9-10

Appendix Table S1. Plasmids used in this study, Related to Methods

Plasmid	Gene		
pSN184	YIp	ADE2/ URA3	pTDH3-HMG2-GFP
pSN149	2μ	URA3	pGAL- BIRA-3xFLAG
pSN148	2μ	URA3	pGAL-DFM1-BIRA-3xFLAG
pSN246	YCp	URA3	pORM2-RFP
pSN247	YCp	LEU2	pLCB1-RFP
pSN266	YCp	URA3	pORM2-GFP
pSN267	YCp	URA3	pORM2-3D-GFP
pSN268	YCp	URA3	pORM2-3A-GFP
pSN60	YCp	LEU2	pDFM1-L64V
pSN90	YCp	LEU2	pDFM1
pSN93	YCp	LEU2	pDFM1-K67E
pSN230	YCp	LEU2	pDFM1-R98L, S99A, S100A, Q101L
pSN95	YCp	LEU2	pDFM1-F58S
pSN162	YCp	LEU2	pDFM1-5Ashp-3HA
pSN161	YCp	LEU2	pDER1-SHP-3HA
pSN164	YCp	LEU2	pDFM1-AR-3HA
pSN163	YCp	LEU2	pDFM1-Ax$_3$G-3HA
pSN269	YCp	TRP1	pORM2-RFP

pSN261	YCp	TRP1	CHS5-mCherry
pSN258	YCp	LEU2/HIS3	SEC61-mCherry
pSN252	YCp	URA3	HDEL-GFP

Appendix Table S2. Yeast strains used in this study, Related to Methods

Strain	Genotype	Reference
SEN 1	*Mata ade2-101 met2 lys-801 ura3-52 trp1::hisG leu2 his3Δ200*	This study
BY4741	*Mata met15Δ0 his3Δ1 leu2Δ0 ura3Δ0*	This study
SEN 322	*SEN1 2μ::URA3::GAL1pr-Dfm1-BIRA-FLAG*	This study
SEN 324	*SEN1 2μ::URA3::GAL1pr- BIRA-FLAG*	This study
SEN 167	*SEN1 dfm1Δ::KanMX ade2-101::ADE2::URA3::TDH3pr-HMG2-GFP 2μ::URA3::GAL1pr-Dfm1-BIRA-3xFLAG*	This study
SEN 168	*SEN1 dfm1Δ::KanMX ade2-101::ADE2::URA3::TDH3pr- HMG2-GFP 2μ::URA3::GAL1pr-BIRA-3xFLAG*	This study
SEN 169	*SEN1 dfm1Δ::KanMX ade2-101::ADE2::URA3::TDH3pr- HMG2-GFP 2μ::URA3*	This study
SEN 280	*BY4741 DFM1::DFM1-GFP::HIS*	This study
SEN 439	*BY4741 DFM1::DFM1-GFP::HIS CEN::URA3:: prORM2-RFP*	This study
SEN 438	*BY4741 DFM1::DFM1-GFP::HIS CEN::LEU2:: prLCB1-RFP*	This study
SEN 635	*SEN1 dfm1Δ::KanMX*	This study
SEN 637	*SEN1 tsc3Δ::NatR*	This study
SEN 638	*SEN1 dfm1Δ::KanMX tsc3Δ::NatR*	This study
SEN 640	*SEN1 orm1Δ::KanMX*	This study
SEN 641	*SEN1 orm1Δ::KanMX tsc3Δ::NatR*	This study
SEN 644	*SEN1 orm2Δ::KanMX*	This study
RHY 646	*SEN1 orm2Δ::KanMX tsc3Δ::NatR*	This study
SEN 285	*SEN1 dfm1Δ::KanMX orm1Δ::NatR*	This study

SEN 291	*SEN1 dfm1Δ::KanMX orm2Δ::NatR*	This study
SEN 280	*BY4741 orm1Δ::KanMX*	This study
SEN 281	*BY4741 orm2Δ::KanMX*	This study
SEN 369	*BY4741 orm1Δ::KanMX orm2Δ::LEU2*	This study
SEN 282	*BY4741 Lcb2-DaMP*	This study
SEN 266	*BY4741 dfm1Δ::KanMX Lcb2-DaMP*	This study
SEN 392	*SEN1 sac1Δ:NatR*	This study
SEN 301	*SEN1 dfm1Δ::KanMX sac1Δ:NatR*	This study
SEN 599	*SEN1 CEN::URA3:: prORM2-RFP*	This study
SEN 659	*SEN1 dfm1Δ::KanMX CEN::URA3:: prORM2-RFP*	This study
SEN 615	*SEN1 der1Δ::KanMX CEN::URA3:: prORM2-RFP*	This study
SEN 623	*SEN1 dfm1Δ::KanMX CEN::URA3:: prORM2-RFP CEN::LEU2:: prDFM1-3xHA*	This study
SEN 624	*SEN1 dfm1Δ::KanMX CEN::URA3:: prORM2-RFP CEN::LEU2*	This study
SEN 625	*SEN1 dfm1Δ::KanMX CEN::URA3:: prORM2-RFP CEN::LEU2:: prDER1-SHP1*	This study
SEN 629	*SEN1 dfm1Δ::KanMX CEN::URA3:: prORM2-RFP CEN::LEU2:: prDFM1-5Ashp*	This study
SEN 626	*SEN1 dfm1Δ::KanMX CEN::URA3:: prORM2-RFP CEN::LEU2:: prDFM1-Ax3G*	This study
SEN 628	*SEN1 dfm1Δ::KanMX CEN::URA3:: prORM2-RFP CEN::LEU2:: prDFM1-AR*	This study
SEN 635	*SEN1 dfm1Δ::KanMX CEN::URA3:: prORM2-RFP CEN::LEU2:: prDFM1-F58S*	This study
SEN 632	*SEN1 dfm1Δ::KanMX CEN::URA3:: prORM2-RFP CEN::LEU2:: prDFM1-L64V*	This study
SEN 634	*SEN1 dfm1Δ::KanMX CEN::URA3:: prORM2-RFP CEN::LEU2:: prDFM1-K67E*	This study
SEN 630	*SEN1 dfm1Δ::KanMX CEN::URA3:: prORM2-RFP CEN::LEU2:: prDFM1-R98L, S99V, S100V, Q101L*	This study
SEN 598	*SEN1 hrd1Δ::KanMX CEN::URA3:: prORM2-RFP*	This study

SEN 604	*SEN1 doa10Δ::NatR CEN::URA3:: prORM2-RFP*	This study
SEN 607	*SEN1 asi1Δ::HphMX CEN::URA3:: prORM2-RFP*	This study
SEN 622	*SEN1 tul1Δ::KanMX CEN::URA3:: prORM2-RFP*	This study
SEN 606	*SEN1 cdc48Δ::cdc48-2::NatR CEN::URA3:: prORM2-RFP*	This study
SEN 608	*SEN1 hrd2-1 CEN::URA3:: prORM2-RFP*	This study
SEN 345	*SEN1 hrd3Δ::KanMX*	This study
SEN 346	*SEN1 tsc3Δ::KanMX hrd3Δ::NatR*	This study
SEN 437	*SEN1 tsc3Δ::KanMX hrd1Δ::NatR*	This study
SEN 3	*SEN1 hrd1Δ::KanMX*	This study
SEN 652	*SEN1 doa10Δ::HphMx*	This study
SEN 654	*SEN1 doa10Δ:: HphMx tsc3Δ::KanMX*	This study
SEN 649	*SEN1 der1Δ::NatR*	This study
SEN 650	*SEN1 der1Δ::NatR tsc3Δ::KanMX*	This study
SEN 521	*BY4741 LCB1-GFP::HIS3*	This study
SEN 522	*BY4741 LCB2-GFP::HIS3*	This study
SEN 523	*BY4741 ORM1-GFP::HIS3*	This study
SEN 524	*BY4741 ORM2-GFP::HIS3*	This study
SEN 525	*BY4741 SAC1-GFP::HIS3*	This study
SEN 526	*BY4741 TSC3-GFP::HIS3*	This study
SEN 527	*BY4741 YPK1-GFP::HIS3*	This study
SEN 528	*BY4741 TSC10-GFP::HIS3*	This study
SEN 662	*SEN1 dfm1Δ::KanMX orm1Δ::NatR CEN::LEU2::prDFM1-3xHA*	This study
SEN 661	*SEN1 dfm1Δ::KanMX orm1Δ::NatR*	This study

	CEN::LEU2	
SEN 675	*SEN1 dfm1Δ::KanMX orm1Δ::NatR CEN::LEU2::prDER1-SHP*	This study
SEN 676	*SEN1 dfm1Δ::KanMX orm1Δ::NatR CEN::LEU2::prDFM1-5Ashp*	This study
SEN 677	*SEN1 dfm1Δ::KanMX orm1Δ::NatR CEN::LEU2::prDFM1-AR*	This study
SEN 678<br>SEN 665	*SEN1 dfm1Δ::KanMX orm1Δ::NatR CEN::LEU2::prDFM1-Ax3G*<br>*SEN1 dfm1Δ::KanMX orm1Δ::NatR CEN::LEU2::prDFM1-L64V*	This study<br>This study
SEN 664	*SEN1 dfm1Δ::KanMX orm1Δ::NatR CEN::LEU2::prDFM1-F58S*	This study
SEN 666	*SEN1 dfm1Δ::KanMX orm1Δ::NatR CEN::LEU2::prDFM1-K67E*	This study
SEN 667	*SEN1 dfm1Δ::KanMX orm1Δ::NatR CEN::LEU2::prDFM1-R98L, S99V, S100V, Q101L*	This study
SEN 552	*SEN1 orm1Δ::NatR*	This study
SEN 551	*SEN1 orm1Δ::NatR hrd1Δ::KanMX*	This study
SEN 543	*SEN1 hrd2-1*	This study
SEN 544	*SEN1 cdc48::cdc48-2::NatR*	This study
SEN 545	*SEN1 doa10Δ:: HphMx*	This study
SEN 546	*SEN1 hrd2-1 orm1Δ::NatR*	This study
SEN 547	*SEN1 cdc48::cdc48-2::NatR orm1Δ::KanMx*	This study
SEN 548	*SEN1 doa10Δ:: HphMx orm1Δ::NatR*	This study
SEN 671	*SEN1 orm2Δ::KanMX tsc3Δ::NatR CEN::URA3::prORM2-3R-GFP*	This study
SEN 672	*SEN1 orm2Δ::KanMX tsc3Δ::NatR CEN::URA3::prORM2-3A-GFP*	This study

SEN 673	*BY4741 orm2Δ::KanMX CEN::URA3::prORM2-3R-GFP*	This study
SEN 674	*BY4741 orm2Δ::KanMX CEN::URA3::prORM2-3A-GFP*	This study
SEN 679	*SEN1 CEN::TRP1::ORM2-RFP CEN::URA3::HDEL-GFP*	This study
SEN 680	*SEN1 dfm1Δ::KanMX CEN::TRP1::ORM2-RFP CEN::URA3::HDEL-GFP*	This study
SEN 681	*SEN1 tul1Δ::KanMX CEN::TRP1::ORM2-RFP CEN::URA3::HDEL-GFP*	This study
SEN 682	*SEN1 tul1Δ::KanMX CEN::TRP1::ORM2-RFP CEN::URA3:VPS4-GFP*	This study
SEN 683	*SEN1 CEN::URA3::ORM2-3R-GFP CEN::LEU2::HIS3::SEC61-mCherry*	This study
SEN 684	*SEN1 CEN::URA3::ORM2-3R-GFP CEN::TRP1::CHS5-mCherry*	This study
SEN 685	*SEN1 dfm1Δ::KanMX CEN::URA3::ORM2-3R-GFP CEN::LEU2::HIS3::SEC61-mCherry*	This study
SEN 686	*SEN1 dfm1Δ::KanMX CEN::URA3::ORM2-3R-GFP CEN::TRP1::CHS5-mCherry*	This study
SEN 687	*SEN1 CEN::URA3::ORM2-3A-GFP CEN::LEU2::HIS3::SEC61-mCherry*	This study
SEN 688	*dfm1Δ::KanMX CEN::URA3::ORM2-3A-GFP CEN::LEU2::HIS3::SEC61-mCherry*	This study
SEN 708	*SEN1 dfm1Δ::KanMX CEN::URA3:: prORM2-GFP CEN::LEU2:: prDFM1-3xHA*	This study
SEN 709	*SEN1 dfm1Δ::KanMX CEN::URA3:: prORM2-3D-GFP CEN::LEU2:: prDFM1-3xHA*	This study
SEN 710	*SEN1 dfm1Δ::KanMX CEN::URA3:: prORM2-3A-GFP CEN::LEU2:: prDFM1-3xHA*	This study
SEN 711	*SEN1 dfm1Δ::KanMX CEN::URA3:: prORM2-GFP CEN::LEU2*	This study
SEN 712	*SEN1 dfm1Δ::KanMX orm2Δ::NatR CEN::LEU2:: prDFM1-3xHA*	This study
SEN 713	*BY4741 ORM2-GFP::HIS3 CEN::LEU2:: prDFM1-3xHA*	This study
SEN 714	*BY4741 ORM2-GFP::HIS3 ypk1Δ::KanMX CEN::LEU2:: prDFM1-3xHA*	This study
SEN 715	*BY4741 ORM2-GFP::HIS3 npr1Δ::KanMX CEN::LEU2:: prDFM1-3xHA*	This study
SEN 716	*BY4741 CEN::LEU2:: prDFM1-3xHA*	This study

Appendix Table S3. Reagents used in this study. Related to Methods

REAGENT or RESOURCE	SOURCE	IDENTIFIER
Antibodies		
Mouse monoclonal anti-GFP	Clontech Laboratories, Inc.	Cat#632381; RRID: AB_2313808
Mouse monoclonal anti-HA	Thermo Fisher Scientific	Cat#32-6700; RRID: AB_2533092
Rabbit polyclonal anti-myc	Genscript	Cat#A00172; RRID: AB_914457
Rabbit polyclonal anti-Cdc48	Gift from Randolph Hampton	Neal et al., 2016
Mouse monoclonal anti-PGK	Thermo Fisher Scientific	Cat#459250; RRID: AB_2569747
Mouse monoclonal anti-Ubiquitin	Gift from Richard Gardner	This study
Mouse monoclonal anti-FLAG	Sigma-Aldrich	Cat# F3165 RRID:AB_259529
Rabbit Polyclonal anti-Sec61	Gift from Randolph Hampton	(Flagg et al., 2021)
Rabbit polyclonal anti-Tul1	Gift from Peter Espenshade	Tong et al., 2014
Rabbit polyclonal anti-Dsc2	Gift from Peter Espenshade	Tong et al., 2014
Rabbit polyclonal anti-Orm1	Gift from Teresa Dunn	Gable et al., 2000
Rabbit polyclonal anti-Lcb1	Gift from Teresa Dunn	Gable et al., 2000
Rabbit polyclonal anti-Lcb2	Gift from Teresa Dunn	Gable et al., 2000
**Bacterial Strain**		
Escherichia coli DH5 alpha Competent Cells	Thermo Fisher Scientific	Cat#18265017
**Chemicals, Peptides, and Recombinant Proteins**		
MG132 (benzyloxycarbonyl-Leu-Leu-aldehyde)	Sigma-Aldrich	474787; CAS: 133407-82-6
Cycloheximide	Sigma-Aldrich	C7698; CAS: 66-819
Protein A Sepharose	GE Healthcare	17-0780-01
GFP-Trap Agarose	ChromoTek	RRID: AB_2631357
RFP-Trap Agarose	ChromoTek	RRID: AB_2631362
Anti-HA Agarose	Thermo Fisher Scientific	Cat# 26181
Estreptavidina C1 Dynabeads™ MyOne™	Thermo Fisher Scientific	Cat# 65001
n-Dodecyl β-D-maltoside	Sigma-Aldrich	Cat# D4641

Primers for gene knockout		Primer name:
ATGACCGAATTAGATTATCAAGGAACTGCT GACATGGAGGCCCAGAATACCCTCCTTGAC		ORM1 For
TCAACTAATTTGGGCGCGACCTGTGATACC CAGTATAGCGACCAGCATTCACATACGATT		ORM1 Rev
ATGATTGACCGCACTAAAAACGAATCTCCA GACATGGAGGCCCAGAATACCCTCCTTGAC		ORM2 For
TTAATCTCTTTTTAAAGGATCCGGCTTGGA CAGTATAGCGACCAGCATTCACATACGATT		ORM2 Rev
ATGGCACACATCCCAGAGGTTTTACCCAAA GACATGGAGGCCCAGAATACCCTCCTTGAC		LCB1 For
TTATTTATTAGATTCTTGGCAACAGGCAAG CAGTATAGCGACCAGCATTCACATACGATT		LCB1 Rev
ATGACACAACATAAAAGCTCGATGGTGTAC GACATGGAGGCCCAGAATACCCTCCTTGAC		TSC3 For
AAGGAAGCAATACTTTAGTATACCGAAGAA CAGTATAGCGACCAGCATTCACATACGATT		TSC3 Rev
ATGGATGTTGATTCTGACGTTAATGTCTC GACATGGAGGCCCAGAATACCCTCCTTGAC		DOA10 For
TTAACTTTCATCTGGTAAATTTTCTAAAGC CAGTATAGCGACCAGCATTCACATACGATT		DOA10 Rev
ATGGTGCCAGAAAATAGAAGGAAACAGTTG GACATGGAGGCCCAGAATACCCTCCTTGAC		HRD1 For
CTAGATATGCTGGATAAATTTATCTGGTAT CAGTATAGCGACCAGCATTCACATACGATT		HRD Rev
ATGATAACACTCTTATTATACCTGTGCGTA GACATGGAGGCCCAGAATACCCTCCTTGAC		HRD3 For
TCATATGGCGAATATCTGAACATTGAAGTC CAGTATAGCGACCAGCATTCACATACGATT		HRD3 Rev
ATGGATGCTGTAATACTGAATCTCTTAGGC GACATGGAGGCCCAGAATACCCTCCTTGAC		DER1 For
TTAGGGTGTTTCAGTGTTGCGGAACCAGTC CAGTATAGCGACCAGCATTCACATACGATT		DER1 Rev
ATGTATTCTTGGAAGTCAAAGTTTAAGTTT GACATGGAGGCCCAGAATACCCTCCTTGAC		YPK1 For
CTATCTAATGCTTCTACCTTGCACCATTGA CAGTATAGCGACCAGCATTCACATACGATT		YPK1 Rev
ATGTCTTCATTAACTCGATTGCTACAGGAA GACATGGAGGCCCAGAATACCCTCCTTGAC		NPR1 For
TTATTGATTATTTTGCTTTTTCTTTTTCTT CAGTATAGCGACCAGCATT		NPR1 Rev
