# Supplementary material for: An ERAD‐independent role for rhomboid pseudoprotease Dfm1 in mediating sphingolipid homeostasis
Source: EMBO J. 2022 Nov 9;42(4):e112275. doi: 10.15252/embj.2022112275 (PMC9929635; doi:10.15252/embj.2022112275)
Supplement: Supplementary file 2 — Expanded View Figures PDF [file EMBJ-42-e112275-s002.pdf]

Expanded View Figures

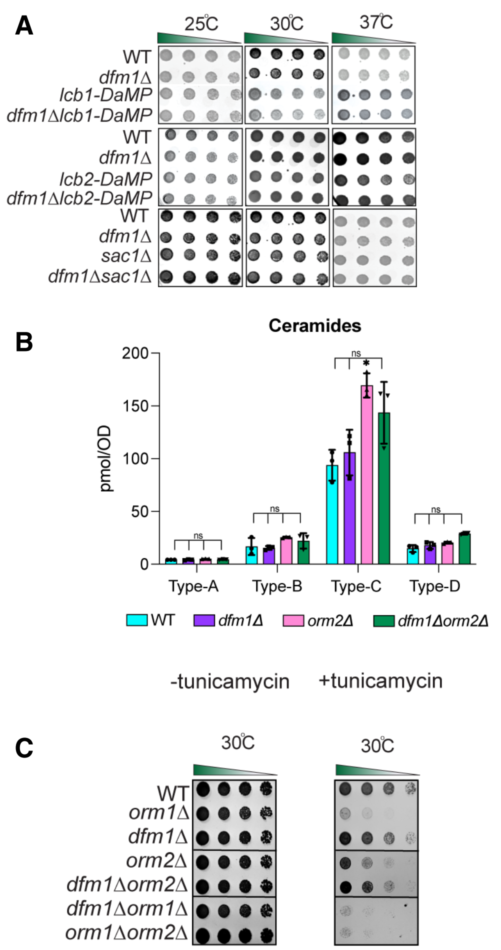

Figure EV1. Dfm1 does not genetically interact with Lcb1, Lcb2, and Sac1. Related to Fig 3.

A Indicated strains were spotted fivefold dilutions on SC plates in triplicates, and plates were incubated at room temperature, 30°C, and 37°C (3 biological replicates, 2 technical replicates; $n = 3$). *Upper panel*: WT, *dfm1Δ*, *Lcb1-DaMP, and dfm1ΔLcb1*-DaMP were compared for growth by dilution assay. *Middle panel*: WT, *dfm1Δ*, *Lcb2-DaMP, and dfm1ΔLcb2*-DaMP were compared for growth by dilution assay. *Bottom panel*: WT, *dfm1Δ, and dfm1Δ sac1Δ* were compared for growth by dilution assay.

B WT, *dfm1Δ*, *orm2Δ*, and *orm2Δdfm1Δ* cells were grown to log phase at 30°C and lipids were extracted and subjected to LC–MS/MS. A-, B-, C-, and D-type ceramides containing C16, C18, C20, C22, C24, and C26 fatty acids were measured (3 biological replicates; $n = 3$). Values represent the means ± S.E.M. Statistically significant differences compared to WT cells are indicated (pairwise Dunnett's test followed by Bonferroni's *post hoc* analysis; ns, non-significant, $*P < 0.05$).

C Serial dilution growth was performed on YPD plates in the presence or absence of 1 μg/ml tunicamycin using WT, *orm1Δ*, *orm2Δ*, *dfm1Δ*, *dfm1Δorm1Δ*, *orm1Δorm2Δ*, and *dfm1Δorm2Δ* cells (3 biological replicates, 2 technical replicates; $n = 3$).

© 2022 The Authors

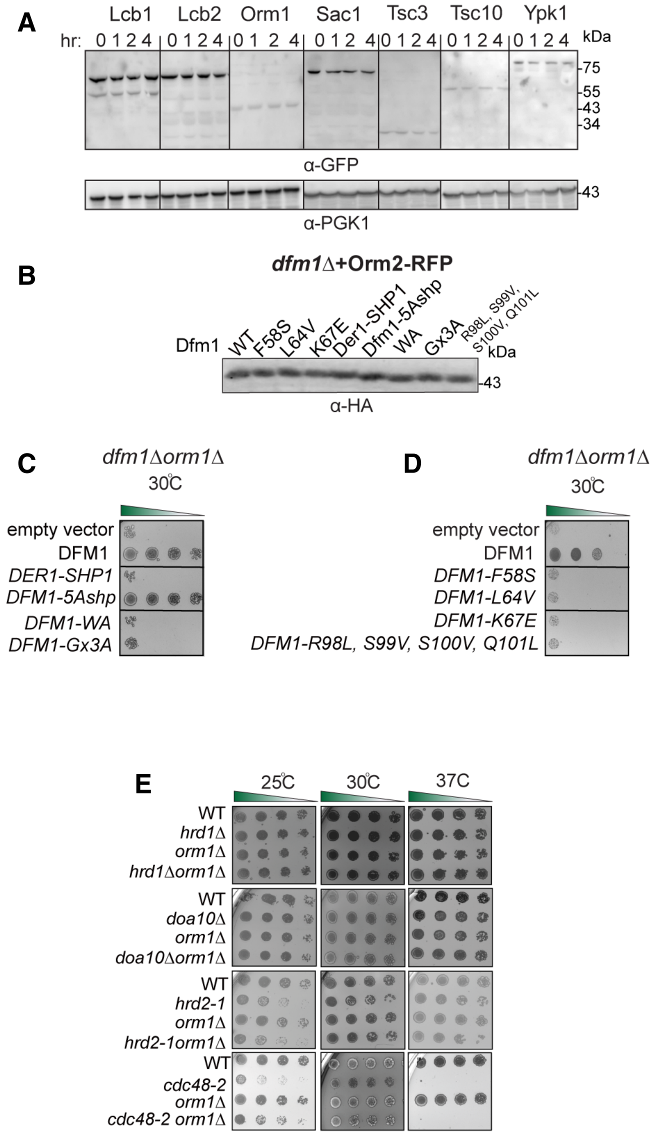

Figure EV2. Orm2 is degraded in WT strains. Related to Fig 5.

A In the indicated WT strains, degradation of Lcb1-GFP, Lcb2-GFP, Orm2-GFP, Orm2-GFP, Sac1-GFP, Tsc3-GFP, Tsc10-GFP, and Ypk1-GFP was measured by CHX-chase assay (3 biological replicates; $n = 3$). Cells were analyzed by SDS–PAGE and immunoblotted with α-GFP.

B Steady-state levels of Dfm1 and corresponding Dfm1 mutants. Cells were analyzed by SDS–PAGE and immunoblotted with α-HA.

C Serial dilution growth assay was performed on *dfm1Δorm1Δ* and strains with DFM1, DER1-SHP, DFM1-AA, DFM1-Ax3A, DFM1-5Ashpmtnt, and empty vector add back (3 biological replicates, 2 technical replicates; $n = 3$).

D Same as (C), except serial dilution growth assay was performed on *dfm1Δorm1Δ* strains with L1 mutant add back: F58S, L64V, K67E, and TMD2 quad mutant add back: DFM1-R98L, S99V, S100V, and Q101L. Indicated strains were grown on SC-Leu plates at room temperature, 30°C, and 37°C, and imaged on Day 2 and Day 7 (3 biological replicates, 2 technical replicates; $n = 3$).

E ERAD mutants do not genetically interact with *orm1Δ*. Indicated strains were spotted fivefold dilutions on SC plates in triplicate, and plates were incubated at room temperature, 30°C, and 37°C (3 biological replicates, 2 technical replicates; $n = 3$).

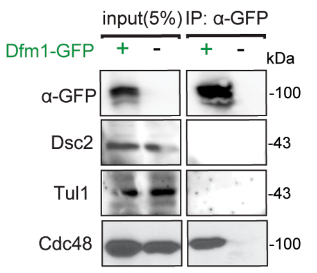

Figure EV3. Dfm1 does not interact with EGAD components. Related to Fig 6.

Dfm1-GFP binding to EGAD members, Dsc2 and Tul1, were analyzed by co-IP. As a negative control, cells not expressing Dfm1-GFP were used (2 biological replicates; $n = 2$).

© 2022 The Authors

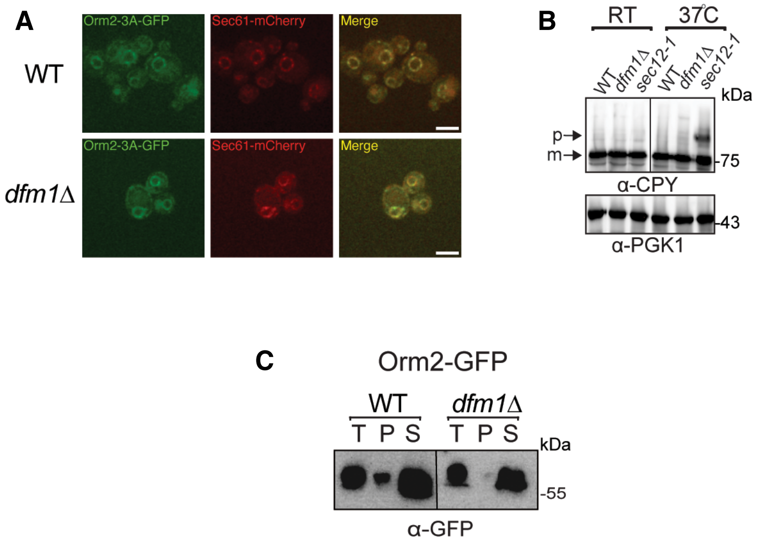

Figure EV4. Orm2-3A accumulates exclusively in the ER. Related to Fig 7.

A Fluorescence imaging was performed as in Fig 7B except for WT and *dfm1Δ* cells expressing Orm2-3A-GFP was used. Sec61-RFP (ER marker, red) was used to test for co-localization with Orm2-3A-GFP (2 biological replicates; $n = 2$). *Arrowheads* indicate Orm2 co-localizing in post-ER compartments. Scale bar, 5 μm.

B *dfm1Δ* cells do not abrogate COPII-mediated export of CPY. The indicated cells were either grown at room temperature or shifted to non-permissive growth at 37°C. Cells were analyzed by SDS–PAGE and immunoblotted for CPY with α-CPY and PGK1 with α-PGK1.

C Western blot of aggregated versus soluble Orm2-GFP at the ER. Lysates from WT and *dfm1Δ* cells containing ORM2-GFP were blotted using anti-GFP to detect Orm2. P is ER aggregated fraction and S is ER soluble fraction.

© 2022 The Authors
