# Supplementary material for: An ERAD‐independent role for rhomboid pseudoprotease Dfm1 in mediating sphingolipid homeostasis
Source: EMBO J. 2022 Nov 9;42(4):e112275. doi: 10.15252/embj.2022112275 (PMC9929635; doi:10.15252/embj.2022112275)
Supplement: Supplementary file 5 — PDF+ [file EMBJ-42-e112275-s003.pdf]

Article

TRANSPARENT PROCESS OPEN ACCESS

THE EMBO JOURNAL

An ERAD-independent role for rhomboid pseudoprotease Dfm1 in mediating sphingolipid homeostasis

Satarupa Bhaduri[1,†], Analine Aguayo[1,†], Yusuke Ohno[2], Marco Proietto[1], Jasmine Jung[1], Isabel Wang[1], Rachel Kandel[1], Narinderbir Singh[1], Ikran Ibrahim[1], Amit Fulzele[‡], Eric J Bennett[1], Akio Kihara[2] & Sonya E Neal[1,*]

Abstract

Nearly one-third of nascent proteins are initially targeted to the endoplasmic reticulum (ER), where they are correctly folded and assembled before being delivered to their final cellular destinations. To prevent the accumulation of misfolded membrane proteins, ER-associated degradation (ERAD) removes these client proteins from the ER membrane to the cytosol in a process known as retrotranslocation. Our previous work demonstrated that rhomboid pseudoprotease Dfm1 is involved in the retrotranslocation of ubiquitinated membrane integral ERAD substrates. Herein, we found that Dfm1 associates with the SPOTS complex, which is composed of serine palmitoyltransferase (SPT) enzymes and accessory components that are critical for catalyzing the first rate-limiting step of the sphingolipid biosynthesis pathway. Furthermore, Dfm1 employs an ERAD-independent role for facilitating the ER export and endosome- and Golgi-associated degradation (EGAD) of Orm2, which is a major antagonist of SPT activity. Given that the accumulation of human Orm2 homologs, ORMDLs, is associated with various pathologies, our study serves as a molecular foothold for understanding how dysregulation of sphingolipid metabolism leads to various diseases.

Keywords endoplasmic reticulum; ERAD; Orm1/2; rhomboid pseudoprotease; sphingolipid homeostasis

Subject Categories Membranes & Trafficking; Post-translational Modifications & Proteolysis

DOI 10.15252/embj.2022112275 | Received 2 August 2022 | Revised 20 September 2022 | Accepted 19 October 2022 | Published online 9 November 2022

The EMBO Journal (2023) 42: e112275

Introduction

The endoplasmic reticulum (ER) carries out a vast range of functions including protein synthesis and transport, protein folding, lipid and steroid synthesis, carbohydrate metabolism, and calcium storage. Almost all eukaryotic membranes and secreted proteins are co-translationally imported into the ER where they are subsequently folded (Wang & Dehesh, 2018; Sicari *et al*, 2019). Proteins frequently fail to fold or assemble properly, at which point they are eliminated by ER-associated degradation (ERAD; Ruggiano *et al*, 2014; Mehrtash & Hochstrasser, 2019; Sun & Brodsky, 2019).

ERAD describes a range of pathways that target and ubiquitinate a large repertoire of secretory and membrane substrates for proteasomal degradation. To date, most of the knowledge about ERAD has been obtained in yeast and mammals. In yeast, ERAD substrates are classified according to the location of their lesions and are referred to as ERAD-L (lesion in luminal domain), ERAD-M (lesion within the transmembrane domain), and ERAD-C (lesion in the cytosolic domain). The HMG-CoA reductase degradation (HRD) pathway utilizes the E3 ligase, Hrd1, to target ERAD-M and ERAD-L substrates, and the degradation of alpha 2 (DOA) pathway utilizes the E3 ligase, Doa10, to target ERAD-C substrates (Hampton *et al*, 1996; Hiller *et al*, 1996; Laney & Hochstrasser, 2003; Carvalho *et al*, 2006; Foresti *et al*, 2013). Moreover, unassembled ER subunits escaping to the inner nuclear membrane (INM) are targeted by the Asi complex (Foresti *et al*, 2014; Natarajan *et al*, 2020). ERAD pathways are much more diverse in mammalian cells due to the complexity of lesions or degrons within ERAD substrates (Leto *et al*, 2019). Although there are no Asi homologs present in mammals, there are at least 20 ER-localized ubiquitin ligases characterized to date that contribute to ERAD (Leto *et al*, 2019; Fenech *et al*, 2020). A common theme for all ERAD pathways is the removal or retrotranslocation of ubiquitinated substrates from the ER membrane or INM followed by degradation by the proteasome (Hampton & Sommer, 2012). Retrotranslocation has been well characterized in yeast, with two derlins, Der1 and Dfm1, serving as major mediators of retrotranslocation for ERAD-L and ERAD-M substrates, respectively (Neal *et al*, 2018; Wu *et al*, 2020). Moreover, previous structural studies suggest that the multi-membrane spanning yeast E3 ligases,

1 Department of Cell and Developmental Biology, School of Biological Sciences, University of California San Diego, La Jolla, CA, USA
2 Laboratory of Biochemistry, Faculty of Pharmaceutical Sciences, Hokkaido University, Sapporo, Japan
*Corresponding author. Tel: +1 858 822 1488; E-mail: seneal@ucsd.edu
†These authors contributed equally to this work as first (co-)authors
‡Present address: Institute of Molecular Biology, Mainz, Germany

© 2022 The Authors. Published under the terms of the CC BY 4.0 license.

Hrd1 and Doa10, function as channels for the retrotranslocation of luminal and single-spanning membrane substrates, respectively (Wu *et al*, 2020; Schmidt *et al*, 2020a, 2020b). No analogous channel for multi-spanning membrane substrates had been determined, until Neal *et al* (2018) identified the yeast derlin, Dfm1, as a major retrotranslocation factor for a subset of membrane substrates (Neal *et al*, 2018).

Dfm1 is an ER-resident multi-spanning membrane protein and is classified as a rhomboid pseudoprotease. Recently, we showed that Dfm1 utilizes its conserved rhomboid protein residues for substrate engagement and its lipid-thinning properties to allow retrotranslocation of multi-spanning membrane substrates (Nejatfard *et al*, 2021). To identify interacting partners of Dfm1 that may assist with retrotranslocation, we employed proximity-based labeling followed by mass spectrometry. Remarkably, we identified several proteins enriched with Dfm1, which are known to play a role in the sphingolipid biosynthesis pathway. Sphingolipids constitute a major class of lipids defined by their amino-alcohol backbone with mainly 18 carbons and are synthesized in the ER from non-sphingolipid precursors (Hannun & Obeid, 2018). Modification of this basic structure gives rise to the vast family of sphingolipids, which have essential roles in cell signaling and function. Serine palmitoyltransferase (SPT) is the first rate-limiting enzyme in the *de novo* synthesis of sphingolipids, and its sole function is to catalyze the initial step in sphingolipid biosynthesis by converting serine and palmitoyl-CoA into a sphingolipid precursor, 3-keto-sphinganine (Hanada, 2003). SPT is essential for the viability of all eukaryotic cells, and mutations of SPT are linked to hereditary sensory neuropathy type 1 (HSAN1) and early-onset amyotrophic lateral sclerosis (ALS; Bode *et al*, 2015; Mohassel *et al*, 2021). Accordingly, SPT serves as the key point for the regulation of sphingolipid biosynthesis. SPT forms the SPOTS complex, comprised of Orm1 and Orm2 (members of the orosomucoid (ORM) gene family), Tsc3, and Sac1. The SPOTS complex is highly conserved from yeast to mammals (Breslow *et al*, 2010). Functionally, Orm1, Orm2, and phosphoinositide phosphatase Sac1 are evolutionarily conserved negative regulators of SPT, while Tsc3 is a positive regulator.

Previous studies have demonstrated that levels of several SPOT complex members and sphingolipid biosynthesis enzymes are regulated through protein degradation pathways in order to control sphingolipid levels. For example, the TORC2–Ypk1 signaling axis phosphorylates Orm2, triggering its export from the ER to the Golgi, where it is selectively ubiquitinated by the Dsc complex before being retrotranslocated and degraded by the cytosolic proteasome. This pathway is known as ER Golgi-associated degradation (EGAD; Schmidt *et al*, 2019, 2020a, 2020b). Another study analyzing the systematic turnover of proteins in yeast revealed that several enzymes and regulators involved in the *de novo* sphingolipid biosynthesis pathway are degraded in separate organelles, such as the Golgi and vacuole (Christiano *et al*, 2020). Although many enzymes and regulators associated with sphingolipid biosynthesis reside in the ER, an ER-localized regulator for sphingolipid homeostasis has not yet been identified. In this study, we report a novel role for the ER-resident Dfm1 in maintaining sphingolipid homeostasis. We find that Dfm1 physically and genetically interacts with SPOTS complex components. This includes a genetic interaction with TSC3, a positive regulator of SPT, whose function is essential for stimulating SPT activity at 37°C. Specifically, loss of Dfm1 rescues the growth lethality of *tsc3*Δ cells by increasing ceramide and complex sphingolipid levels. DFM1 also genetically interacts with ORM1, a negative regulator of SPT activity, in which *orm1*Δ*dfm1*Δ cells have an exacerbated growth defect due to increased flux in sphingolipid biosynthesis. Finally, we provide the first evidence that Dfm1 is required for Orm2 degradation, a function that is independent of Dfm1's canonical ERAD-M retrotranslocation function. We confirm the independence of Dfm1's ERAD function and demonstrate that the EGAD client, Orm2, does not require ERAD or inner nuclear membrane-associated degradation (INMAD) pathways, which is in agreement with an earlier study (Schmidt *et al*, 2019). To better understand the role of Dfm1 in Orm2 degradation, we show that loss of Dfm1 results in the accumulation of phosphorylated Orm2 at the ER, suggesting a novel role for Dfm1 in controlling Orm2 export from the ER and its subsequent degradation by EGAD. We further show that Dfm1 does not function with COPII dynamics and trafficking, but functions upstream of ER export where Dfm1 interacts with Ypk1-dependent phosphorylated Orm2. Overall, our work identifies the highly conserved derlin Dfm1 as a critical mediator of sphingolipid homeostasis and provides a new therapeutic target for maladies associated with a dysregulation in sphingolipid homeostasis.

Results

Derlin Dfm1 interacts with members of the sphingolipid biosynthetic pathway

To identify potential Dfm1 interacting proteins, proximity-dependent biotin identification (BioID) was employed. Briefly, BirA-3xFLAG was fused to Dfm1 to survey for potential interacting partners (Fig 1A). Because Dfm1 included an added BirA-3xFLAG epitope at the C-terminus, we wished to confirm that the tag did not affect the expression and function of Dfm1. To this end, tagged DFM1 was placed under a galactose-inducible promoter (GAL_{pr}) and cells expressing GAL-driven Dfm1-BirA-3xFLAG were grown in the presence of 2% galactose. Under these conditions, induced expression of Dfm1 was observed at the expected molecular weight of ~ 60 kDa (Fig 1B). BirA-3xFLAG alone expressed at both the expected size of ~ 25 kDa and at a larger size, which most likely represents BirA aggregates (marked by asterisks, Fig 1B). To test whether Dfm1-BirA-3xFLAG function is still intact, we performed cycloheximide (CHX) chase of a well-characterized Dfm1 substrate, Hmg2 (Hampton *et al*, 1996). We observed Hmg2-GFP degradation upon add back of Dfm1-BirA-3xFLAG while Hmg2-GFP degradation was stabilized with both empty vector and BirA-3xFLAG alone (Fig 1C). To validate the identification of Dfm1 interactors via biotinylation, cells were treated with biotin and biotinylated proteins were enriched with streptavidin beads. As expected, the ATPase Cdc48, which has previously been shown to bind directly to Dfm1 (Sato & Hampton, 2006; Neal *et al*, 2018), was enriched in the biotin-treated samples, whereas neither Cdc48 nor Dfm1 was enriched in untreated or BirA-3xFLAG alone cells (Fig 1D). Next, proteins that were enriched with streptavidin beads were digested to obtain tryptic peptides and analyzed by LC/MS/MS. Quantified proteins were mapped on volcano plots based on the significance and the ratio between biotin-treated Dfm1-BirA-3xFLAG and untreated

© 2022 The Authors

control cells. High-confidence interacting proteins were identified using DEP and Maxquant analysis (Fig 1E; Dataset EV1; Zuzow *et al*, 2018). By applying gene ontology (GO) enrichment analyses for the sets of Dfm1-interacting proteins identified, we found GO terms related to "Ceramide Metabolic Process" to be the most enriched (Fig 1F). The interactions were validated by the presence of several ERAD components (Hrd1, Cdc48, and proteasome subunits: Rpt2 and Pre9). Interestingly, closer analysis revealed unexpected interactions with SPOTS complex members (Orm1, Tsc3, and Lcb2). Taken together, these results suggest that our data have a high level of confidence and represent a rich source of Dfm1 interactome proteins, which include members of the sphingolipid biosynthesis pathway.

To validate the interaction of Dfm1 with SPOTS complex members, we performed co-immunoprecipitation (co-IP). Cells co-expressing Dfm1-GFP and members of the SPOTS complex (Lcb1-

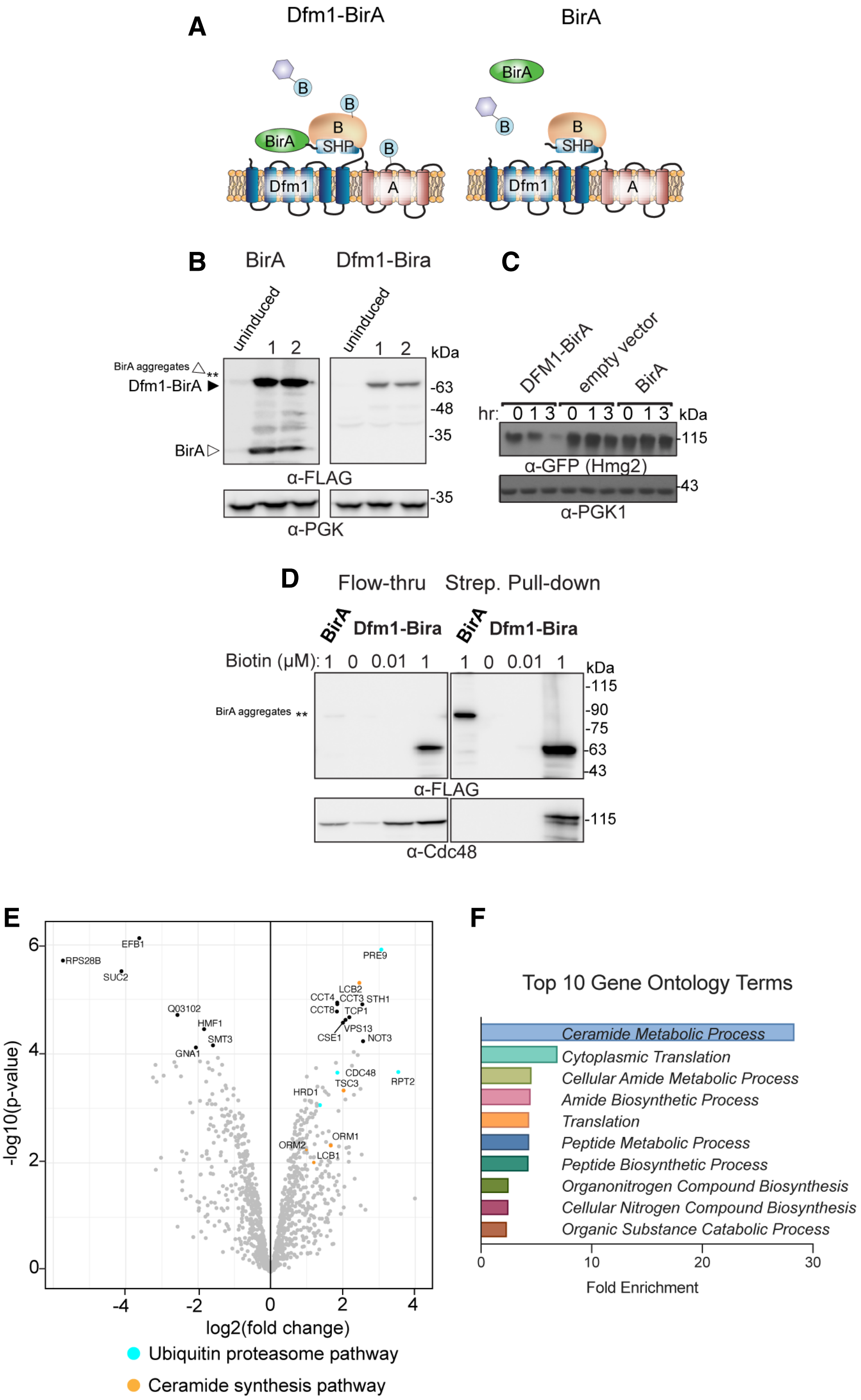

Figure 1.

Figure 1. BioID proximity-based labeling to identify interaction partners of Dfm1.

A Schematic of Dfm1 fused with a biotin ligase, BirA, at the C-terminus along with untagged Dfm1. Cartoon representation of the labeling of rhomboid pseudoprotease Dfm1 with the biotin ligase, BirA.

B GALpr-Dfm1-BirA-Flag and GALpr-BirA-Flag levels were measured by western blotting with α-FLAG at 0 (uninduced) vs. 5 h post-galactose induction (3 biological replicates; $n = 3$).

C Dfm1-BirA is still functional and able to degrade Hmg2-GFP. *dfm1Δ* + Hmg2-GFP strains containing DFM1-BIRA, empty vector, or BIRA-only add backs were grown to log phase and degradation was measured by CHX. After CHX addition, cells were lysed at the indicated times and analyzed by SDS–PAGE and immunoblotted for Hmg2-GFP with α-GFP.

D Yeast strains expressing Dfm1-Bira and BirA-only negative control were incubated with different amounts of biotin: 0, 0.1, and 1 mM. *dfm1Δ* + Hmg2-GFP. Microsomes were isolated from each strain and subjected to streptavidin pulldown (3 biological replicates; $n = 3$). Flow-through and pull-down fractions were detected by western blotting for Dfm1-BirA with α-Flag and Cdc48 with α-Cdc48 antibodies.

E A volcano plot showing enrichment versus the significance of proteins identified in Dfm1-BirA experiments relative to control (BirA only) experiments. Components that were significantly enriched were ERAD components in blue (Hrd1, Cdc48, Pre9, and Rpt2) and sphingolipid biosynthesis in orange (Lcb2, Tsc3, and Orm1). Statistical analysis of interactome data was carried out using differential enrichment analysis of proteomics (DEP). Filter cut-offs were set at $\log_2$FC ≥2, *P*-value of ≤ 0.01, and at least two quantitative peptide features.

F Top 10 gene ontology (GO) terms and their enrichment factor for the set of genes with the highest significance and fold enrichment.

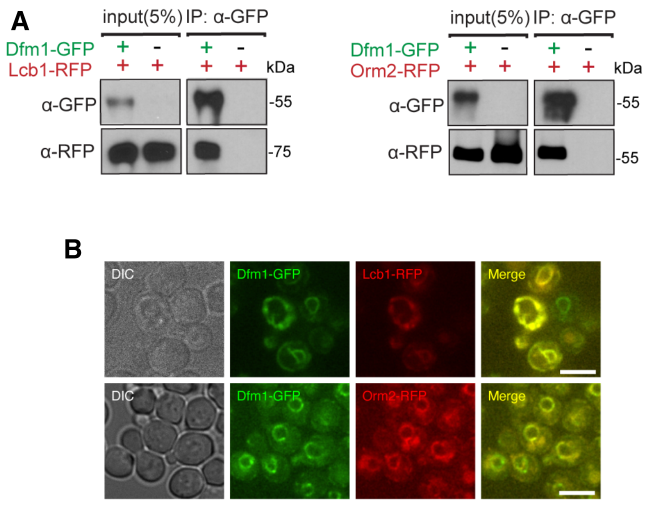

Figure 2. Dfm1 colocalizes and binds to SPOTS complex proteins.

A Dfm1-GFP binding to Lcb2-RFP and Orm2-RFP was analyzed by co-IP. As a negative control, cells not expressing Dfm1-GFP were used (3 biological replicates; $n = 3$).

B Dfm1-GFP colocalizes with Lcb1-RFP and Orm22-RFP. Strains were grown to mid-exponential phase in minimal media GFP, and RFP fluorescence was examined on an AxioImager.M2 fluorescence microscope using a 100x objective and 28HE-GFP or 20HE-rhodamine filter sets (3 biological replicates; $n = 3$; Zeiss). Scale bar, 5 μm.

RFP and Orm2-RFP) were subjected to immunoprecipitation via GFP Trap. Notably, Lcb1-RFP and Orm2-RFP co-immunoprecipitated with Dfm1-GFP, whereas no detectable association was seen in control cells without Dfm1-GFP (Fig 2A). These interactions were also validated by fluorescence microscopy, in which the majority of Dfm1-GFP co-localized with Lcb1-RFP and Orm2-RFP at the ER (Fig 2B).

DFM1 genetically interacts with TSC3

We next examined whether DFM1 genetically interacts with SPOTS complex members. The SPOTS complex consists of the SPT enzymes, Lcb1 and Lcb2, and the smaller subunit, Tsc3, which has been required to positively regulate SPT at high temperatures (Gable *et al*, 2000). Furthermore, SPT activity is negatively regulated by two yeast paralogs, Orm1 and Orm2, through direct interactions, and by Sac1, which negatively regulates SPT through an unknown mechanism (Breslow *et al*, 2010; Han *et al*, 2010). To survey for gene interactions, we generated double-mutant yeast strains of *dfm1Δ* along with respective SPOTS complex members and performed serial dilution growth assays to test whether double-knockout cells confer any distinct growth phenotypes compared with WT and single knockouts. To test the involvement of essential enzymes Lcb1/Lcb2 and non-essential regulator Sac1, we utilized *Lcb1-DaMP*, *Lcb2-DamP*, and *sac1Δ* mutants and observed no genetic interactions, since growth of *dfm1ΔLcb1-DaMP*, *dfm1ΔLcb2-DaMP*, and *dfm1Δsac1Δ* was similar to that of WT cells at 25°C, 30°C, and 37°C (Fig EV1A). A small subunit of the SPT, Tsc3, directly interacts with Lcb1/Lcb2 to stimulate their activity and increase the synthesis of the sphingolipid precursor, 3-ketosphinganine. The stimulatory function of Tsc3 is essential at the higher temperature where the *tsc3Δ* temperature-sensitive phenotype is lethal due to a lack of phytosphingosine (PHS) production (Gable *et al*, 2000; Fig 3A). In line with this observation, we observed a growth defect and lethality from *tsc3Δ* cells at 30°C and 37°C, respectively (Fig 3A, *filled triangle*), and rescue of lethality when PHS was supplied to *tsc3Δ* cells (Fig 3B, *right panel*; *filled triangle*). Remarkably, the removal of DFM1 in this background – *dfm1Δtsc3Δ* – completely rescued the lethality at 37°C (Fig 3A, *open circle*). Thus, the removal of DFM1 suppresses *tsc3Δ* lethality at 37°C.

dfm1Δtsc3Δ cells have increased steady-state levels of ceramides and complex sphingolipids

We predicted that the removal of DFM1 was able to reverse the temperature-sensitive lethality in *tsc3Δ* as a result of increased production of sphingolipid precursors. Notably, myriocin is a potent inhibitor of SPT, the first committed step in the sphingolipid biosynthesis pathway, and treatment with myriocin reduces sphingolipid levels in both *S. cerevisiae* and mammals (Breslow, 2013). Because SPT activity is essential, myriocin treatment exacerbates growth due to decreased flux in the sphingolipid biosynthesis pathway. We, therefore, wanted to test whether *dfm1Δtsc3Δ* cells are resistant to myriocin inhibition. To this end, a sublethal dose of myriocin was

 © 2022 The Authors

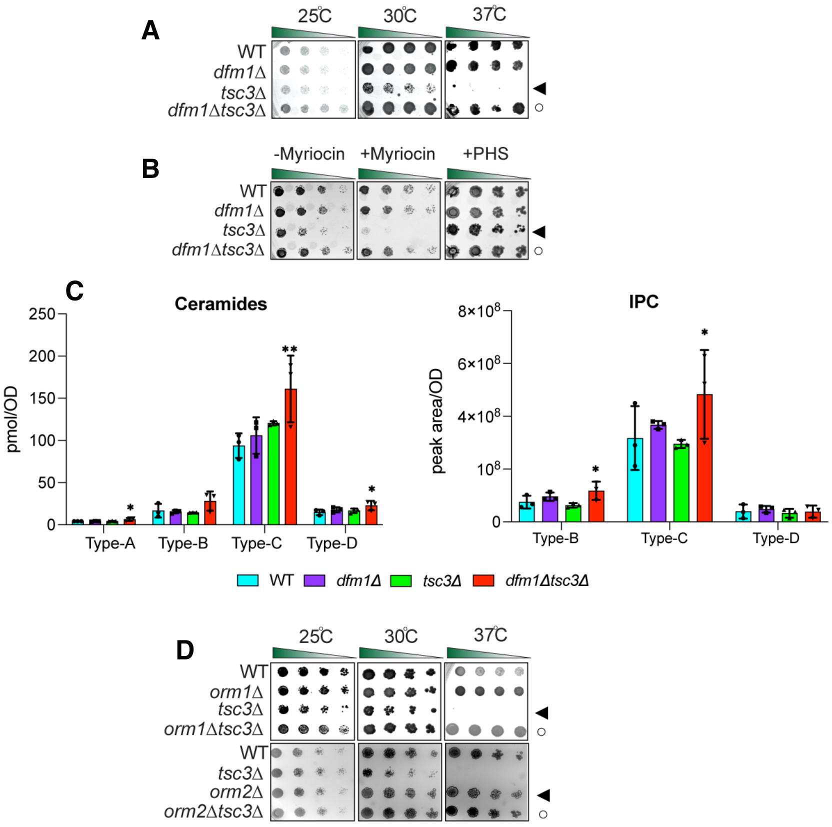

Figure 3. Dfm1 genetically interacts with Tsc3.

A Indicated strains were spotted fivefold dilutions on synthetic complete (SC) plates, and plates were incubated at room temperature, 30 and 37°C (3 biological replicates, 2 technical replicates; *n* = 5). WT, *dfm1*Δ, *tsc3*Δ, and *dfm1*Δ*tsc3*Δ were compared for growth in the dilution assay. Arrowhead indicates the growth phenotype of *tsc3*Δ cells; the open circle indicates the growth phenotype of *dfm1*Δ*tsc3*Δ cells.

B *dfm1*Δ*tsc3*Δ confers resistance to myrocin. WT, *dfm1*Δ, *tsc3*Δ, and *dfm1*Δ*tsc3*Δ strains were grown to log-phase in YPD medium, and fivefold serial dilutions of cultures were spotted on (SC) plates containing either drug vehicle alone, or 1 μM of myriocin and 10 μM of PHS (3 biological replicates, 2 technical replicates; *n* = 5). Arrowhead indicates the growth phenotype of *tsc3*Δ cells; the open circle indicates the growth phenotype of *dfm1*Δ*tsc3*Δ cells.

C WT, *dfm1*Δ, *tsc3*Δ, and *dfm1*Δ*tsc3*Δ cells were grown to log-phase at 30°C and lipids were extracted and subjected to LC–MS/MS. A-, B-, C-, and D-type ceramides containing C16, C18, C20, C22, C24, and C26 fatty acid (left graph) and B-, C-, and D-type IPCs containing C24 and C26 fatty acid (right graph) were measured. Values represent the means ± S.E.M (3 biological replicates; *n* = 3). Pairwise Dunnett's test followed by Bonferroni's *post hoc* analysis was used to determine statistically significant differences in comparison to WT cells (**P* < 0.05, ***P* < 0.01).

D Indicated strains were spotted fivefold dilutions on SC plates in three biological replicates and two technical replicates (*n* = 5), and plates were incubated at room temperature, 30°C, and 37°C (*n* = 3). *Upper panel*: WT, *orm1*Δ, *tsc3*Δ, *and orm1*Δ*tsc3*Δ were compared for growth by dilution assay. *Middle panel*: WT, *orm2*Δ, *tsc3*Δ, *and orm2*Δ*tsc3*Δ were compared for growth by dilution assay. *Bottom panel*: WT, *orm1*Δ, *orm2*Δ, *tsc3*Δ, *and orm1*Δ*orm2*Δ*tsc3*Δ were compared for growth by dilution assay. Upper panel: arrowhead indicates growth phenotype of *tsc3*Δ cells; the open circle indicates growth phenotype of *orm1*Δ*tsc3*Δ cells. Lower panel: arrowhead indicates growth phenotype of *orm2*Δ cells; the open circle indicates growth phenotype of *orm2*Δ*tsc3*Δ cells.

used in the serial growth assay to reduce sphingolipid synthesis without impairing cell growth. As expected, *tsc3*Δ cells were sensitive to myriocin treatment at 30°C since these cells already have decreased sphingolipid levels (Fig 3B, *left panel*; *filled triangle*). By contrast, the *dfm1*Δ*tsc3*Δ cells were resistant to myriocin treatment, suggesting that these cells have higher levels of sphingolipids (Fig 3B, *left panel*; *open circle*). Indeed, when grown at 30°C, lipidomic analysis demonstrated that *dfm1*Δ*tsc3*Δ cells significantly produced higher levels of ceramides (Type A, C, and D) and complex sphingolipid inositol phosphorylceramide (IPC; Type B & C) in comparison to WT cells (Fig 3C). Altogether, *dfm1*Δ*tsc3* cells appear to produce higher steady-state levels of ceramide and complex sphingolipids.

DFM1 genetically interacts with ORM1

Because the removal of DFM1 leads to higher levels of ceramides and complex sphingolipids in *tsc3*Δ cells, we hypothesized that Dfm1 is antagonizing the sphingolipid biosynthesis pathway. If this hypothesis is correct, *dfm1*Δ cells should phenocopy both *orm1*Δ and *orm2*Δ cells, which are established negative regulators of the SPT enzymes, in the growth assays. To test this hypothesis, *orm1*Δ*tsc3*Δ and *orm2*Δ*tsc3*Δ cells were generated and employed in the growth assays at 25°C, 30°C, and 37°C (Fig 3D). Under these conditions, both *orm1*Δ and *orm2*Δ phenocopied *dfm1*Δ; both *orm1*Δ*tsc3*Δ and *orm2*Δ*tsc3*Δ cells were able to rescue the temperature-sensitive lethality displayed by *tsc3*Δ cells (Fig 3D).

© 2022 The Authors

Because *dfm1*Δ cells phenocopy both *orm1*Δ and *orm2*Δ cells, we next examined whether DFM1 genetically interacts with either ORM1 or ORM2. Although no growth defect was observed for *dfm1*Δ*orm2*Δ cells, we did observe a growth defect in *dfm1*Δ*orm1*Δ cells at room temperature, 30°C, and 37°C, suggesting that DFM1 functions with ORM1 in a parallel pathway (Fig 4A, *filled triangle*). Furthermore, lipidomic analysis confirmed that *dfm1*Δ*orm2*Δ cells showed no increase in ceramides compared with WT cells. This was in contrast to *dfm1*Δ*orm1*Δ cells where there were significant changes in ceramide levels compared to WT cells (Figs 4B and

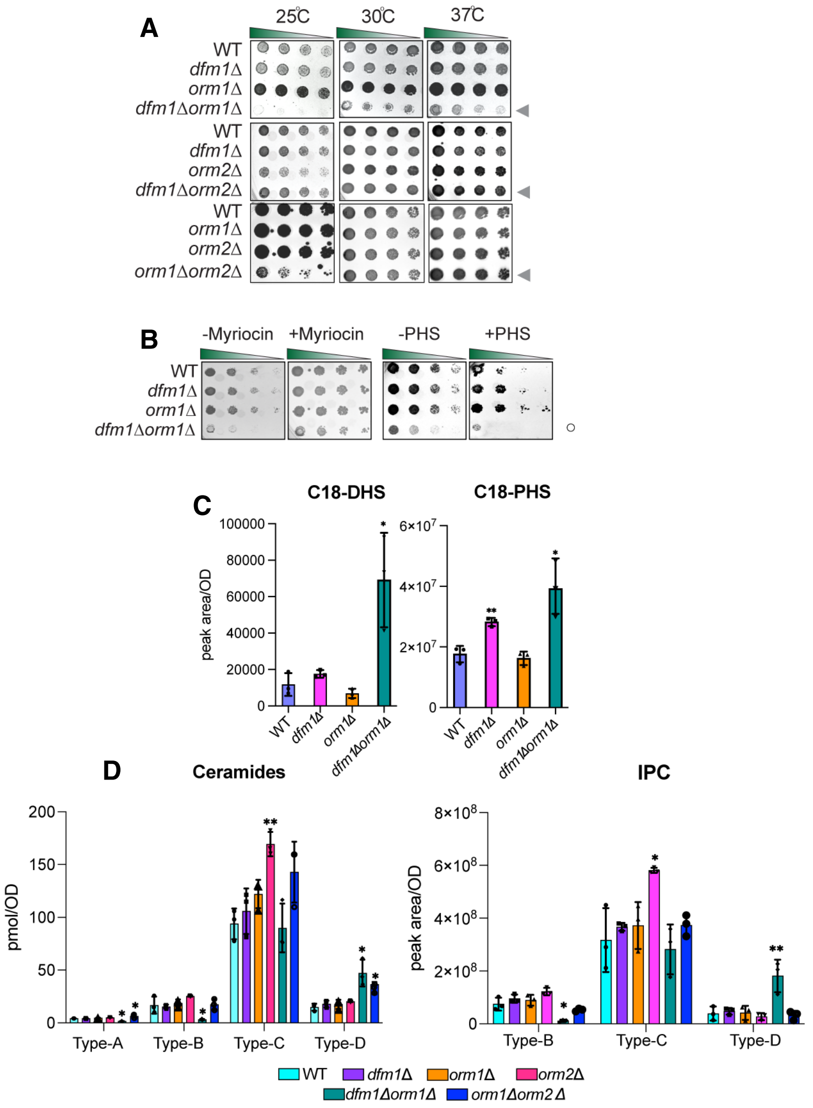

Figure 4. Dfm1 genetically interacts with Orm1.

A Indicated strains were spotted fivefold dilutions on SC plates in three biological replicates and two technical replicates ($n = 5$), and plates were incubated at room temperature, 30°C, and 37°C. *Upper panel*: WT, *dfm1*Δ, *orm1*Δ, *and dfm1*Δ*orm1*Δ were compared for growth by dilution assay. *Middle panel*: WT, *dfm1*Δ, *orm2*Δ, *and orm2*Δ*tsc3*Δ were compared for growth by dilution assay. *Bottom panel*: WT, *dfm1*Δ, *orm1*Δ, *orm2*Δ, *orm2*Δ, and *orm1*Δ*orm2*Δ were compared for growth by dilution assay. Upper, middle, and lower panel: gray arrowhead depicts growth phenotype of *dfm1*Δ*orm1*Δ, *dfm1*Δ*orm2*Δ, and *orm1*Δ*orm2*Δ, respectively.

B *dfm1*Δ*orm1*Δ confers resistance to myriocin and sensitivity to PHS. WT, *dfm1*Δ, *orm1*Δ, and *dfm1*Δ*orm1*Δ strains were grown to log phase in SC medium, and fivefold serial dilutions of cultures were spotted on YPD plates containing either drug vehicle alone, or 1 mM of myriocin and 10 μM of PHS with each condition performed in three biological replicates and two technical replicates ($n = 5$). Plates were incubated at room temperature and photographed after 3 days. Open circle indicates growth phenotype of *dfm1*Δ*orm1*Δ cells.

C WT, *dfm1*Δ, *orm1*Δ, and *orm1*Δ*dfm1*Δ cells were grown to log-phase at 30°C, and lipids were extracted and subjected to LC–MS/MS. C18 PHS and DHS levels were measured as described in Methods (3 biological replicates; $n = 3$). Values represent the means ± S.E.M. Statistically significant differences compared to WT cells are indicated (pairwise Dunnett's test followed by Bonferroni's *post hoc* analysis; $^{*}P < 0.05$, $^{**}P < 0.01$).

D WT, *dfm1*Δ, *orm1*Δ, *orm2*Δ, *dfm1*Δ*orm1*Δ, and *orm1*Δ*orm2*Δ cells were grown to log-phase at 30°C, and lipids were extracted and subjected to LC–MS/MS. A-, B-, C-, and D-type ceramides containing C16, C18, C20, C22, C24, and C26 fatty acid (left graph) and B-, C-, and D-type IPCs containing C24 and C26 fatty acid (right graph) were measured (3 biological replicates; $n = 3$). Values represent the means ± S.E.M. Statistically significant differences compared to WT cells are indicated (pairwise Dunnett's test followed by Bonferroni's *post hoc* analysis; $^{*}P < 0.05$, $^{**}P < 0.01$).

 © 2022 The Authors

EV1B). Orm1 and Orm2 have been shown to coordinate lipid homeostasis with ER protein quality control. This was demonstrated through the growth sensitivity of *orm1Δorm2Δ* cells to agents that increase protein misfolding in the ER (Han *et al*, 2010). Because DFM1 genetically interacts with ORM1, we surmise that *dfm1Δorm1Δ* cells should also exhibit growth sensitivity to ER protein misfolding agents (Fig EV1C). We performed growth assays on plates containing tunicamycin, an inhibitor of N-linked glycosylation. Growth sensitivity to tunicamycin was observed for *orm1Δ* and *orm2Δ* cells whereas exasperated growth defects were observed for *dfm1Δorm1Δ* and *orm1Δorm2Δ*, but not *dfm1Δorm2Δ* cells, further confirming that DFM1 genetically interacts with ORM1 and not ORM2. Finally, cells lacking ORM1 and ORM2 exhibit a growth defect (Fig 4A, *bottom panel*) due to an increased flux in *de novo* sphingolipid synthesis, and the knockout cells were more resistant to myriocin inhibition (Breslow *et al*, 2010; Han *et al*, 2010). Given the growth defect seen in *dfm1Δorm1Δ* cells, we reasoned that the flux in sphingolipid synthesis should be similarly increased. Notably, *dfm1Δorm1Δ* cells were resistant to myriocin treatment (Fig 4B, *left panel*; *open circle*) and sensitive to exogenously added PHS (Fig 4B, *right panel*; *open circle*), since *dfm1Δorm1Δ* cells already exhibit higher levels of sphingolipids. Both dihydrosphingosine (DHS) and phytosphingosine (PHS) are early precursors of the sphingolipid biosynthesis pathway and are derivatives of long-chain bases (LCBs). Lipidomic analysis via mass spectrometry showed that C18-DHS levels were significantly higher in *dfm1Δorm1Δ* cells than in WT cells. Also, C18-PHS levels were significantly higher in both *dfm1Δ* and *dfm1Δorm1Δ* cells in comparison to WT cells, suggesting there is increased flux in sphingolipid biosynthesis in *dfm1Δorm1Δ* cells (Fig 4C). In contrast, the levels of ceramides and complex sphingolipids varied in *dfm1Δorm1Δ* cells. There were higher levels of ceramide and complex sphingolipids (Type D) and lower levels of ceramide (Type A, B, and C) and complex sphingolipids (Type B) in *dfm1Δorm1Δ* cells in comparison to WT cells (Fig 4D). Notably, *orm1Δorm2Δ* control cells also exhibited similar fluctuating levels of the varying types of ceramides and complex sphingolipids (Fig 4D). Despite varying levels of ceramides and complex sphingolipids, *dfm1Δorm1Δ* cells have higher LCB levels and are resistant to myriocin treatment, which suggests that the major physiological effect of *dfm1Δorm1Δ* cells is from increased SPT activity (Fig 4B and C).

Orm2 is targeted by Dfm1 for degradation

Given the myriad biological processes carried out by sphingolipids, it is not surprising that disruptions to sphingolipid homeostasis have deleterious effects and must be tightly regulated. One possible mode of regulation is through the regulated degradation of key enzymes and regulators of sphingolipid biosynthesis in a manner analogous to the regulated degradation of Orm2 by EGAD to establish sphingolipid homeostasis. We, therefore, tested whether key enzymes or regulators within the sphingolipid biosynthesis pathway are targeted for Dfm1-mediated degradation by performing cycloheximide (CHX)-chase assays on candidate substrates (Lcb1, Lcb2, Orm1, Orm2, Sac1, Tsc3, Ypk1, and Tsc10), which function in either SPT synthesis or regulation (Fig EV2A). Of these, Orm2 was rapidly degraded in wild-type strains and its degradation was completely prevented in *dfm1Δ* cells (Fig 5A). The yeast paralog of Dfm1, Der1, has a strong broad role in retrotranslocating ERAD-L substrates (Wu *et al*, 2020). We therefore directly tested the role of Der1 in Orm2 degradation using the CHX-chase assay and found that in both WT and *der1Δ* cells, Orm2 was still degraded (Fig 5B). These results imply that the degradation of Orm2 is specifically dependent on derlin Dfm1 and not Der1.

Derlin Dfm1's Cdc48 recruitment function is not required for Orm2 degradation

We have previously identified specific motifs and residues of Dfm1 that are critical for its ERAD retrotranslocation function. Accordingly, we wished to test the importance of these motifs/residues for Orm2 degradation by performing CHX-chase assays. Dfm1 possesses a unique C-terminal SHP box motif, which recruits the ATPase, Cdc48, directly to the ER surface (Neal *et al*, 2018). Cdc48 functions as an energy source for membrane substrate retrotranslocation and as a retrochaperone where it acts to maintain the solubility of retrotranslocated membrane substrates prior to proteasome degradation (Neal *et al*, 2017). We previously demonstrated that mutations within the SHP box, Dfm1-5Ashp, ablate Cdc48 recruitment and the retrotranslocation function of Dfm1 (Fig 5C; Neal *et al*, 2018). We also demonstrated that a Der1-SHP chimera, which consists of Der1, the paralog of Dfm1, fused to the cytoplasmic SHP tail of Dfm1, supports Cdc48 recruitment via binding of Cdc48 to the chimera's SHP

Figure 5. Dfm1 targets Orm2 for degradation.

A Degradation of Orm2 depends on Dfm1 and not Der1. The indicated strains expressing Orm2-RFP were grown into the log phase and degradation was measured by cycloheximide chase (CHX). After CHX addition, cells were lysed at the indicated times, analyzed by SDS–PAGE, and immunoblotted for Orm2-RFP with α-RFP (3 biological replicates; $n = 3$).
B Same as (A) except degradation of Orm2-RFP was measured in WT and *der1Δ* cells (3 biological replicates; $n = 3$).
C Depiction of Dfm1, Der1, Dfm1-5Ashp, and Der1-Shp. Dfm1 and Der1 are ER-localized membrane proteins with six transmembrane domains (Greenblatt *et al*, 2011). Unlike Der1, Dfm1 has an extended cytoplasmic tail containing two SHP boxes.
D Dfm1's SHP box is not required for degradation of Orm2-RFP. In the indicated strains, degradation of Orm2-RFP was measured by CHX-chase assay. Cells were analyzed by SDS–PAGE and immunoblotted for Orm2-RFP with α-RFP (3 biological replicates; $n = 3$).
E Depiction of Dfm1, which highlights L1, TM2, TM6, and its SHP box domain.
F Table indicating the location and specific function that is impaired for retrotranslocation-deficient Dfm1 mutants (Nejatfard *et al*, 2021).
G Dfm1's WR motif, GxxxG motif, substrate-binding, and lipid-thinning function are required for the degradation of Orm2-RFP. In the indicated strains, degradation of Orm2-RFP was measured by CHX-chase assay. Cells were analyzed by SDS–PAGE and immunoblotted for Orm2-RFP with α-RFP (3 biological replicates; $n = 3$).
H Dfm1 L1 residues are required for binding to Orm2. Orm2-RFP and binding to retrotranslocation-deficient Dfm1 L1 mutants were analyzed by co-IP. The IP was analyzed for the presence of Dfm1-HA. As a negative control, cells not expressing Orm2-RFP were used (3 biological replicates; $n = 3$). Band intensities for all western blots were normalized to PGK1 loading control and quantified by ImageJ.

Data information: t = 0 was taken as 100% and data are represented as mean ± SEM.

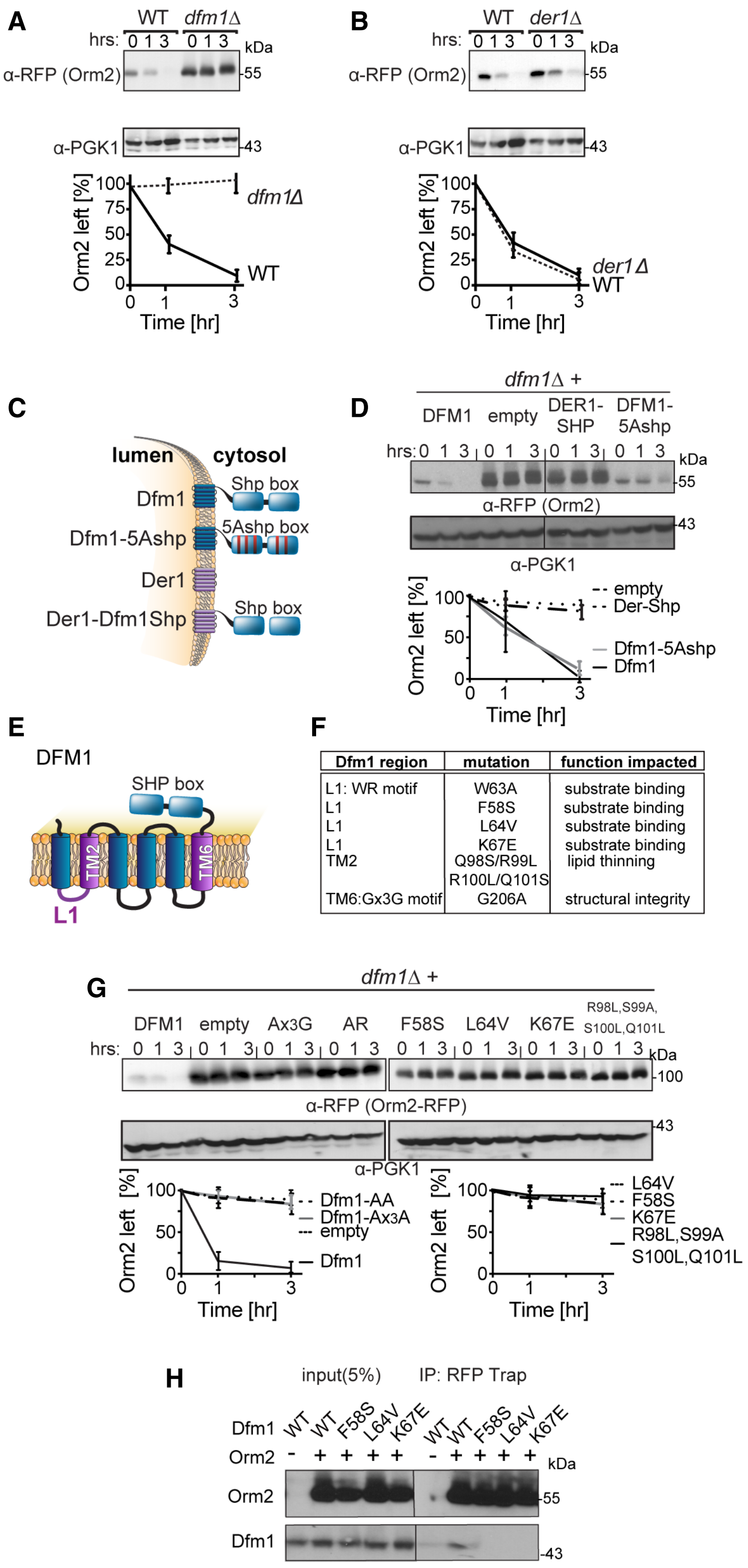

Figure 5.

tail, but does not support retrotranslocation through Der1's transmembrane segment (Fig 5C; Neal *et al*, 2018). We utilized these retrotranslocation-deficient variants in our CHX-chase assay to test whether Dfm1's Cdc48 recruitment function is required for Orm2 degradation. Add back of Der1-SHP in *dfm1*Δ cells impaired Orm2 degradation, whereas Dfm1-5Ashp add back still enabled Orm2

 © 2022 The Authors

degradation (Fig 5D). These results suggest that recruitment of Cdc48 by Dfm1 is dispensable for Orm2 degradation. In addition, the inability of Der1-SHP to facilitate the degradation of Orm2 implies the involvement of additional residues within the transmembrane segments of Dfm1.

Dfm1 contains the highly conserved WR motif in loop 1 (L1) and a Gx3G motif in transmembrane 6 (TM6). Both motifs have previously been substituted for alanine residues (WA and Gx3A) and such mutants are unable to support retrotranslocation (Fig 5E and F; Neal *et al*, 2018). In addition, we have previously identified that the L1 and TM2 regions of Dfm1 are critical for its retrotranslocation function (Fig 5E and F). Specifically, L1 mutants (F58S, L64V, and K67E) impaired membrane substrate binding to Dfm1 and TM2 mutants (R98L, S99V, S100V, and Q101L) impaired Dfm1's lipid thinning distortion function (Nejatfard *et al*, 2021). The lipid distortion function of Dfm1 increases lipid permeability to aid the extraction of integral membrane substrates from the lipid bilayer. All Dfm1 mutants have been characterized where they show robust expression at similar levels as wild-type Dfm1 (Fig EV2B; Neal *et al*, 2018; Nejatfard *et al*, 2021). Accordingly, the effect of these retrotranslocation-deficient mutants on Orm2 degradation was tested with the CHX-chase assay. All Dfm1 mutants, with the exception of Dfm1-5Ashp, completely stabilized Orm2 (Fig 5G). We also employed the substrate-binding co-IP assay to analyze the association of Dfm1 L1 mutants with Orm2-RFP. Orm2-RFP was immunoprecipitated with RFP Trap and immunoblotted for L1 Dfm1 mutants with α-HA. There was no detectable association of Orm2 with Dfm1 L1 mutants, implying that all three L1 residues are required for Orm2 binding (Fig 5H). Overall, the conserved rhomboid motifs, WR and Gx3G, the L1 region for substrate binding, and the TM2 region for lipid thinning, are all required for Orm2 degradation. We employed another functional assay for Dfm1 to test whether the retrotranslocation-deficient Dfm1 mutants can restore growth in *dfm1*Δ*orm1*Δ cells, which normally have impaired growth at 37°C due to increased flux in sphingolipid synthesis. To this end, adding back empty vector or wild-type DFM1 to *dfm1*Δ*orm1*Δ cells resulted in the expected impairment and rescue of normal growth, respectively (Fig EV2C and D). Introduction of Dfm1 mutants to *dfm1*Δ*orm1*Δ cells did not rescue growth defects, with the exception of Dfm1-5Ashp, which was able to rescue the growth defect in a manner similar to that of WT Dfm1 (Fig EV2C). Taken together, these data suggest that the substrate-binding, lipid distortion function, and conserved rhomboid motifs, but not Cdc48 recruitment function, of Dfm1 are required for Orm2 degradation.

Orm2 degradation is dependent on EGAD, but not ERAD or INMAD

Given that the Cdc48 recruitment function of Dfm1 is dispensable for Orm2 degradation, it seems likely that Dfm1's retrotranslocation function in ERAD is not required for Orm2 degradation. Accordingly, we wished to survey all protein degradation pathways in which Dfm1 may participate. The secretory pathway possesses several protein quality control pathways including the INM-associated degradation (INMAD), ERAD, and EGAD, which govern both regulated and quality control degradation of INM proteins, ER proteins, and endosomal/Golgi proteins, respectively (Sicari *et al*, 2019; Sun & Brodsky, 2019). All pathways employ dedicated E3 ligases that determine substrate specificity and ubiquitination. Specifically, the Asi and Doa10 E3 ligases mediate INMAD, the Hrd1 and Doa10 E3 ligases mediate ERAD, and Tul1 E3 ligase mediates EGAD. A unifying theme for all protein degradation pathways is that they require the hexameric AAA ATPase, Cdc48, and the proteasome for retrotranslocation and degradation of all substrates. We utilized the CHX-chase assay to test the requirement for all E3 ligases, Cdc48, and the proteasome for the degradation of Orm2. In line with previous studies (Schmidt *et al*, 2019), Orm2 was still degraded with similar kinetics to wild-type strains in *hrd1Δ*, *doa10Δ*, and *asi1Δ* (Fig 6A). These results indicate that Orm2 degradation does not require either the INMAD or ERAD pathways. As expected, Orm2 degradation was completely inhibited in *tul1Δ* cells, *cdc48-2* cells, and proteasome subunit mutant, *hrd2-1* (Fig 6B; Schmidt *et al*, 2019). These observations are in accordance with previous studies and demonstrate that Orm2 ubiquitination, extraction, and proteasome degradation are mediated solely by EGAD (Schmidt *et al*, 2019). To further confirm that Orm2 degradation is independent of ERAD and INMAD, we performed *in vivo* ubiquitination assays on WT, *asi1Δ*, *hrd1Δ*, *doa10Δ*, *tul1Δ*, *cdc48-2*, and *hrd2-1* strains. Strains were lysed and subjected to immunoprecipitation (IP) using anti-RFP antibodies, followed by immunoblotting (IB) with anti-ubiquitin and anti-RFP antibodies. As suggested by CHX-chase experiments conducted by our lab and others (Schmidt *et al*, 2019), the degree of Orm2 ubiquitination in *asi1Δ*, *hrd1Δ*, and *doa10Δ* was similar to that seen in WT strains, demonstrating that the E3 ligases Asi, Hrd1, and Doa10 are not involved in the polyubiquitination of Orm2 (Fig 6C, *lanes 1*, *2*, *3*, *4*). In line with a previous study, the amount of Orm2 ubiquitination is increased in *cdc48-2* and *hrd2-1* cells, suggesting that Orm2 is on the pathway for retrotranslocation and proteasome degradation (Fig 6C, *lanes 8 and 9*; Schmidt *et al*, 2019).

As expected, Orm2 ubiquitination was decreased in *tul1Δ* strains, indicating that Orm2 is ubiquitinated by Tul1-dependent EGAD (Fig 6C, lane 5). To further confirm that Orm2 degradation is independent of EGAD and INMAD, we next tested whether any of the ERAD components besides Dfm1, genetically interacted with Tsc3 and Orm1. Specifically, we examined whether any ERAD mutants phenocopy *dfm1Δtsc3Δ* cells, which rescues lethality at 37°C, or *dfm1Δorm1Δ*, which exhibits a growth defect at 37°C. To this end, double mutants were generated in which *tsc3Δ* or *orm1*Δ was knocked out, along with the following HRD and DOA pathway components: *hrd1Δ*, *hrd3*Δ, *der1*Δ, and *doa10*Δ. In all cases, the HRD and DOA pathway mutants did not phenocopy *dfm1*Δ: *hrd1Δtsc3Δ*, *hrd3Δtsc3Δ*, *der1Δtsc3Δ*, and *doa1Δtsc3Δ* were unable to rescue the temperature-sensitive lethality of *tsc3*Δ (Fig 6D); and *hrd1Δorm1Δ*, *doa10Δorm1Δ*, *hrd2-1orm1Δ*, and *cdc48-2Δorm1Δ* did not exhibit an exacerbated growth defect at 30°C (Fig EV2E). Hence, the DOA and HRD ERAD pathways do not genetically interact with Tsc3 or Orm1. In summary, CHX-chase, genetics, and *in vivo* ubiquitination assays confirmed that Orm2 is degraded solely by the EGAD pathway and not by INMAD or ERAD.

Dfm1 does not function at the post-ubiquitination step of Orm2 degradation pathway

To determine the step at which Dfm1 functions in Orm2 degradation, the ubiquitination status of Orm2 in *dfm1Δ* strains was

© 2022 The Authors

analyzed. We have previously demonstrated that Dfm1 functions at the post-ubiquitination step of ERAD, with an increased degree of polyubiquitination of ERAD-M substrates observed in *dfm1Δ* strains (Neal *et al*, 2018). This was caused by the inability of Dfm1 to retrotranslocate its substrates, resulting in a build-up of polyubiquitinated membrane substrates along the ER membrane. Surprisingly, in *dfm1Δ* strains, the level of Orm2 ubiquitination was decreased to Orm2 similar levels as *tul1Δ* strains. This suggests that in *dfm1Δ* strains, Orm2 does not get ubiquitinated. Moreover, the lack of Orm2 ubiquitination demonstrates *dfm1Δ* strains do not phenocopy retrotranslocation-deficient strains, *cdc48-2*, or the proteasomal mutant *hrd2-1* (Fig 6C, lanes 7,8,9). Hence, Dfm1 does not function in the post-ubiquitination step of EGAD. We also tested the requirement of Der1 for Orm2 ubiquitination and saw no change in Orm2 ubiquitination levels in *der1Δ* strains compared with WT strains (Fig 6C, lanes 1 and 7). Taken together, these data suggest that Dfm1 does not function at the post-ubiquitination step of the Orm2 degradation pathway.

Dfm1 does not directly function in EGAD

Given the requirement for Dfm1 in Orm2 degradation, it is surprising that the Dfm1-dependent ERAD pathway is not involved with Orm2 degradation. It is possible that Dfm1 directly functions in EGAD. To test this hypothesis, we examined the interaction of Dfm1 with the Dsc complex (E3 ligase Tul1 and Dsc2), which mediates substrate detection and ubiquitination within the Golgi in the EGAD pathway. Dfm1-GFP was immunoprecipitated with GFP Trap antibodies followed by SDS–PAGE and immunoblotting for endogenous Tul1 and Dsc2 with anti-Tul1 and anti-Dsc2, respectively. In all cases, no association of Dfm1 with Tul1 and Dsc2 was observed, while Dfm1 was able to associate with Cdc48, as expected

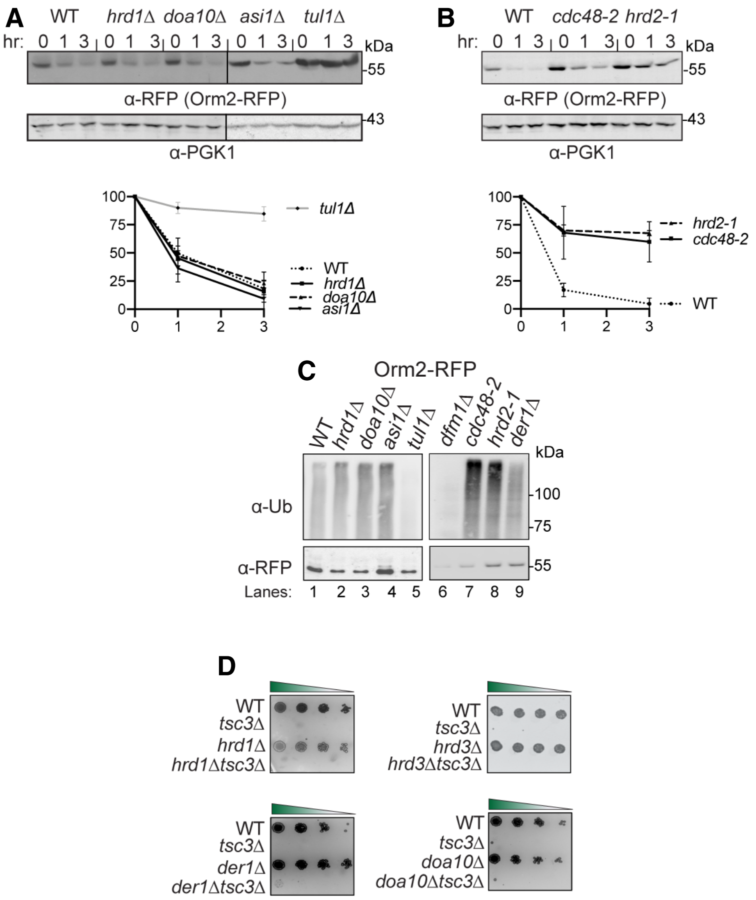

Figure 6. Orm2 degradation requires EGAD but not ERAD or INMAD pathway.

A E3 ligase Tul1 is required for Orm2 degradation. In the indicated strains, degradation of Orm2-RFP was measured by CHX-chase assay. Cells were analyzed by SDS–PAGE and immunoblotted for Orm2-RFP with α-RFP (3 biological replicates; *n* = 3). t = 0 was taken as 100% and data are represented as mean ± SEM.

B Cdc48 and the proteasome are required for Orm2 degradation. Same as (A), except *cdc48-2* and *hrd2-1* were analyzed for Orm2-RFP degradation (3 biological replicates; *n* = 3). t = 0 was taken as 100% and data are represented as mean ± SEM.

C Dfm1 does not function in the post-ubiquitination step of the Orm2 degradation pathway. Indicated strains expressing Orm2-RFP were grown into the log phase. Cells were lysed, and microsomes were collected and immunoprecipitated with α-RFP conjugated to agarose beads. Samples were then subjected to SDS–PAGE and immunoblot by α-Ubiquitin and α-RFP (3 biological replicates; *n* = 3).

D ERAD mutants do not rescue temperature-sensitive lethality of *tsc3Δ*. Indicated strains were grown to log phase in SC and serially diluted cultures were plated on SC plates and incubated at 37°C and imaged on Day 2 (3 biological replicates, 2 technical replicates; *n* = 5).

© 2022 The Authors

(Fig EV3). Moreover, fluorescence microscopy demonstrated that Dfm1 is solely localized in the ER and does not co-localize with Golgi-associated markers (Fig 2B). Finally, there were no significant interactions between Dfm1 and any EGAD components identified from our proteomic analysis (Fig 1E; Dataset EV1).

dfm1Δ cells accumulate phosphorylated Orm2 within the ER

The observation that Dfm1-5Ashp can still facilitate Orm2 degradation suggests that Dfm1-mediated degradation of Orm2 is independent of its canonical retrotranslocation role in ERAD. Hence, we sought to identify the specific step at which Dfm1 functions within the Orm2 degradation pathway. EGAD-mediated degradation of Orm2 is most well characterized in yeast where it consists of five steps: (i) phosphorylation of Orm2 by Ypk1 in the ER, (ii) COPII-mediated export of phosphorylated Orm2 from ER to Golgi and endosome, (iii) polyubiquitination of Orm2 by the E3 ligase Dsc2, (iv) retrotranslocation of substrates from the Golgi/endosome to the cytosol, and (v) degradation of the ubiquitinated substrates by the cytosolic proteasome (Schmidt *et al*, 2019). To determine which step was blocked in Dfm1-deficient cells, we analyzed the phosphorylation status of Orm2 in *dfm1*Δ cells. In *dfm1*Δ, Orm2 phosphorylation was increased to levels similar to those in the Dsc complex mutant *tul1*Δ, a knockout that blocks Orm2 degradation and leads to accumulation of phosphorylated Orm2 (Fig 7A). This indicates *dfm1*Δ cells result in defective trafficking of Orm2 to the Golgi. To validate this in a cellular context, we next utilized live cell imaging fluorescence microscopy to determine the cellular compartment in which Orm2 was accumulating in *dfm1*Δ cells. In line with a previous study, Orm2 accumulated mainly in the early endosomes in *tul1*Δ cells (Schmidt *et al*, 2019), indicating that Orm2 was being routed to the Golgi/endosomes for degradation. By contrast, in *dfm1*Δ cells, Orm2 accumulated mainly at the ER and Orm2 co-localized with an ER, but not an endosome marker (Fig 7B). In parallel, we utilized a phosphomimetic Orm2 variant (Orm2-3D), which has been shown to mimic Ypk1-dependent constitutive phosphorylation and is continuously exported from the ER and degraded via EGAD. Indeed, we and others show that in WT cells, Orm2-3D is rapidly degraded (Fig 7C; Schmidt *et al*, 2019, 2020a, 2020b). Remarkably, by employing a CHX-chase assay, we found that Orm2-3D degradation was completely prevented in *dfm1*Δ cells (Fig 7C). Using microscopy, we confirmed that Orm2-3D remained exclusively in the ER in *dfm1*Δ cells (Fig 7E). By contrast, degradation of an Orm2 phosphonull variant (Orm2-3A), lacking Ypk1 phosphorylation sites, was completely prevented in WT cells (Fig 7C) and we and others showed that Orm2-3A was not exported to the ER (Fig EV4A; Schmidt *et al*, 2019). Because *orm2*Δ cells can rescue *tsc3*Δ lethality, we wanted to test whether Orm2-3D or Orm2-3A elicits the same effect. To test this, either phosphonull Orm2-3A or phosphomimetic Orm2-3D was added to *tsc3*Δ*orm2*Δ cells and the growth assay was employed. As controls, Orm2-3A alone and Orm2-3D cells grew similarly as WT cells, whereas *tsc3*Δ cells exhibited the expected growth lethality at 37°C. Notably, *tsc3*Δ*orm2*Δ cells containing Orm2-3D alleviated *tsc3*Δ lethality, whereas *tsc3*Δ*orm2*Δ cells containing Orm2-3A did not rescue *tsc3*Δ lethality (Fig 7D). These results suggest that two conditions are sufficient in rescuing *tsc3*Δ lethality: (i) absence of Orm2 (*orm2*Δ) or (ii) continuous phosphorylation and degradation of Orm2 via EGAD (Orm2-3D). In summary, based on CHX-chase, live image florescence microscopy, and genetic interaction assays, we demonstrate that Dfm1 is required for the export of phosphorylated Orm2 from the ER to Golgi. The physiological consequence of Dfm1 dysfunction is the retention of Orm2 in the ER, which prevents its subsequent degradation.

In dfm1Δ cells, accumulation of phosphorylated Orm2 leads to increased LCB levels

Our data demonstrate that Dfm1-deficient cells accumulate phosphorylated Orm2 within the ER, the negative regulator of sphingolipid biosynthesis, which raises the question of why increased levels of LCBs, ceramides, and complex sphingolipids were observed in *dfm1*Δ and *dfm1*Δ*tsc3*Δ cells? Our results contrast with our initial expectation that the accumulation of Orm2 would decrease levels of LCB and ceramides, since Orm2 antagonizes the sphingolipid biosynthesis pathway. One possibility is that in *dfm1*Δ cells, ER-localized phosphorylated Orm2 is no longer able to repress SPT activity. To test this possibility, we measured steady-state levels

Figure 7. *dfm1*Δ accumulates phosphorylated Orm2 exclusively in the ER.

A Phosphorylated Orm2 accumulates in the absence of Dfm1. Phos-tag western blot analysis shows that there is an accumulation of phosphorylated Orm2 in *dfm1*Δ cells. The indicated strains were grown to log phase, treated with vehicle or 1.5 μM myriocin for 1 h, and subjected to SDS–PAGE or Phos-tag western blot analysis via blotting for Orm2 with α-RFP and PGK1 with α-PGK1 antibodies (2 biological replicates; *n* = 2).

B Orm2 is retained in the ER in *dfm1*Δ cells. Strains were grown to mid-exponential phase in minimal media and GFP and RFP fluorescence was examined on an AxioImager.M2 fluorescence microscope using a 100x objective and 28HE-GFP or 20HE-rhodamine filter sets (Zeiss; 2 biological replicates *n* = 2). WT, *dfm1*Δ, and *tul1*Δ cells expressing Orm2-RFP. HDEL-GFP (ER marker, green) or VPS4-GFP (endosome marker, green) was used to test for co-localization with Orm2-RFP. Arrowheads indicate Orm2 co-localizing in post-ER compartments. Scale bar, 5 μm.

C *dfm1*Δ cells block the degradation of phosphorylated mimics of Orm2 (Orm2-3D). In the indicated strains, degradation of Orm2-3A-GFP and Orm2-3D-GFP was measured by CHX-chase assay. Cells were analyzed by SDS–PAGE and immunoblotted α-GFP (3 biological replicates; *n* = 3). t = 0 was taken as 100% and data are represented as mean ± SEM.

D Accumulation of phosphorylated Orm2 within the ER is sufficient for rescuing the temperature-sensitive lethality of *tsc3*Δ cells. Indicated strains were spotted fivefold dilutions on SC plates and plates were incubated at 37°C (3 biological replicates, 2 technical replicates; *n* = 5).

E *dfm1*Δ blocks the export of phosphorylated Orm2. Fluorescence imaging was performed as in (B) except WT and *dfm1*Δ cells expressing Orm2-3D-GFP were used (2 biological replicates; *n* = 2). Sec61-mCherry (ER marker, red) or CHS5-mCherry (endosome marker, red) was used to test for co-localization with Orm2-3D-GFP. *Arrowheads* indicate Orm2 co-localizing in post-ER compartments. Scale bar, 5 μm.

F WT and *dfm1*Δ cells expressing either Orm2-3A-GFP or Orm2-3D-GFP were grown to log phase at 30°C and lipids were extracted and subjected to LC–MS/MS. C18 PHS and DHS levels were measured as described in Methods in 3 biological replicates (*n* = 3). Values represent the means ± S.E.M. Statistically significant differences compared to WT cells are indicated (pairwise Dunnett's test followed by Bonferroni's *post hoc* analysis; ***$P < 0.0001$).

© 2022 The Authors

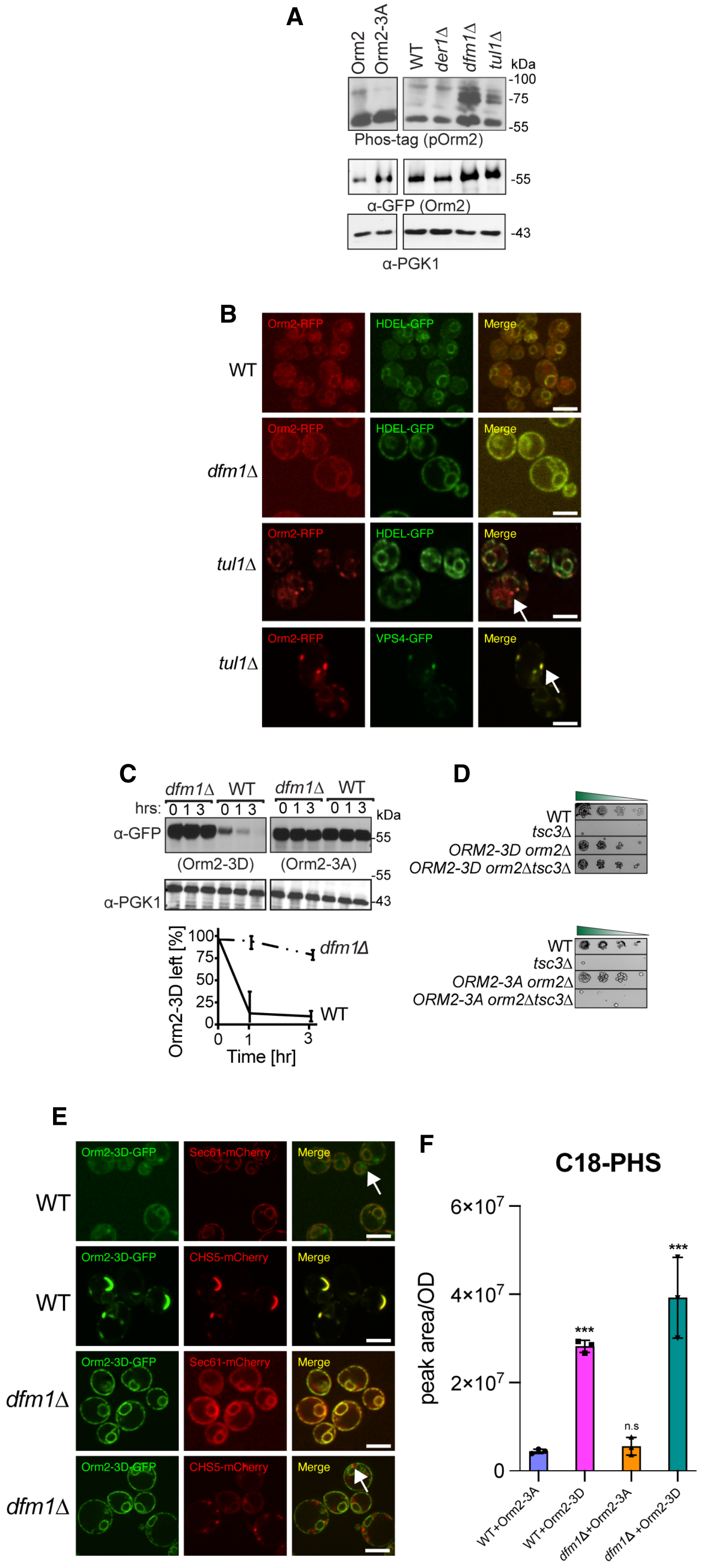

Figure 7.

 © 2022 The Authors

of PHS in WT and *dfm1*Δ cells either expressing phosphomimetic Orm2-3D or phosphonull Orm3A. As expected, in both WT and *dfm1*Δ cells, phosphonull Orm2-3A leads to low steady-state levels of PHS, whereas in WT cells with Orm2-3D, which is constitutively degraded by EGAD, leads to significantly higher levels of PHS (Fig 7F). Notably, *dfm1*Δ cells with Orm2-3D, which is phosphomimetic Orm2 variant that is accumulating in the ER, lead to significantly higher levels of PHS (Fig 7F). This finding suggests that accumulation of phosphorylated Orm2 at the ER does indeed increase SPT activity.

Loss of Dfm1 does not affect COPII-mediated trafficking

Because the loss of Dfm1 resulted in the accumulation of Orm2 in the ER, we directly interrogated the role of Dfm1 in COPII-mediated trafficking. To test whether Dfm1 has a direct function in COPII-mediated trafficking, we analyzed the steady-state levels of COPII cargo substrate, carboxypeptidase Y (CPY), and found that in *dfm1*Δ cells, the mature form of CPY accumulated at similar levels as WT cells (*m*; Fig EV4B). As a control for a deficiency in COPII-mediated export, when *sec12-1* cells were shifted to non-permissive growth temperature at 37°C, there was the expected buildup of the premature form (P; Fig EV4B). Finally, we did not identify significant interactions between Dfm1 and any COPII trafficking components from our proteomic analysis (Fig 1C; Dataset EV1). Taken together, our data suggest that Dfm1 does not directly function in COPII-dependent trafficking.

Dfm1 interacts with Ypk1-dependent phosphorylated Orm2

Because Dfm1 is not functioning directly in ER exit, we hypothesize that Dfm1 is functioning with the SPOTS complex through the interaction of phosphorylated Orm2. To interrogate this, we used co-IP to test interactions of Dfm1 with either phosphomimetic Orm2-3D or phosphonull Orm2-3A. As a negative control, a strain containing an empty vector (instead of Dfm1-HA) was included. We also tested the binding of Dfm1 to an ER membrane protein, Sec61, that is not normally associated with SPOTS complex members and Dfm1. Indeed, we show that Dfm1 is associated with both WT Orm2 or phosphomimetic Orm2-3D and not phosphonull Orm2-3A (Fig 8A). Since Dfm1 no longer associates with Orm2-3A, we next tested whether Orm2-3A affected Dfm1's association with other SPOTS complex members. Notably, when we performed co-IP of cells expressing Orm2-3A, Dfm1 did not interact with Orm1, Lcb1, and Lcb2. This suggests that Dfm1 associates with the SPOTS complex through the binding of Orm2. We confirmed this using co-IP in the *orm2*Δ cell in which Dfm1 no longer interacted with Orm1, Lcb1, and Lcb2 (Fig 8B). Because Ypk1-dependent phosphorylation of Orm2 triggers its ER export and degradation by EGAD, we surmised that the phosphorylation step was required for Orm2 to disassociate from the SPOTS complex. Notably, phosphomimetic Orm2-3D did not abrogate its association with the SPOTS complex members implicating that phosphorylation does not trigger the separation of Orm2 from the complex. In yeast, TORC2-Ypk1 signaling axis is required for Orm2 phosphorylation, which triggers Orm2 degradation by EGAD, whereas TORC1-Npr1 signaling axis is required for Orm2 phosphorylation at distinct sites to stimulate the synthesis of complex sphingolipids (Shimobayashi *et al*, 2013; Schmidt *et al*, 2020a, 2020b). We were interested in investigating the effect of Ypk1-dependent *versus* Npr1-dependent Orm2 phosphorylation on Dfm1 interaction. To address this, co-IP was performed in *ypk1*Δ and *npr1*Δ cells. In *npr1*Δ cells, the SPOTS complex remained intact where Orm2 was associated with Dfm1, Lcb1, Lcb2, and Orm1 (Fig 8C). In contrast, in *ypk1*Δ cells, Orm2 was no longer associated with Dfm1, but remains associated with Lcb1, Lcb2, and Orm1 (Fig 8C). Overall, the findings above demonstrated that Dfm1 associated with the SPOTS complex through binding of Ypk1-dependent phosphorylated Orm2.

Discussion

In this study, we describe a novel role for the derlin rhomboid pseudoprotease, Dfm1, in maintaining sphingolipid homeostasis. The function of Dfm1 in ERAD-M retrotranslocation of misfolded membrane protein substrates has been well established in our laboratory and this study uncovers an additional biological function of Dfm1. The role of Dfm1 in maintaining sphingolipid homeostasis appears to be separate from its role in ERAD-M retrotranslocation. Our study indicates that Dfm1 is required to facilitate the export of phosphorylated Orm2 from the ER and that this function requires substrate-binding and lipid-thinning activities, but not its Cdc48 recruitment function. Specifically, Dfm1 functions immediately downstream of Ypk1-dependent phosphorylation of Orm2 where Dfm1 associates with the SPOTs complex through binding of phosphorylated Orm2 and not unphosphorylated (lacking Ypk1 sites) Orm2 (Fig 8D). Overall, our studies reveal a novel role for rhomboid pseudoproteases in maintaining sphingolipid homeostasis, a function that is independent of their role in ERAD.

To identify Dfm1-interacting partner proteins, we performed proximity-dependent biotinylation (BioID) coupled with mass spectrometry. Several proteins found in close proximity to Dfm1 were involved in the sphingolipid biosynthesis pathway. The first committed step of the sphingolipid biosynthetic pathway is catalyzed by the serine palmitoyl transferase (SPT) complex, which consists of Lcb1, Lcb2, and Tsc3. This step is strictly regulated by Orm1/Orm2 and Sac1, which negatively regulates SPT, and Tsc3, and enhances SPT activity 100-fold. Indeed, we confirmed a physical interaction between Dfm1 and the SPOTS complex members Orm2 and Lcb1. To further explore the relationship between Dfm1 and SPOTS complex members, we examined the genetic interactions between *dfm1*Δ and the knockout of SPOTS complex members or sphingolipid biosynthetic enzymes. We exploited the *tsc3*Δ growth lethality phenotype at 37°C, which is due to Tsc3 being required for enhancing SPT activity at 37°C. Growth assays and lipidomic analyses indicated that *dfm1*Δ cells phenocopy established negative regulators of the SPT enzymes, *orm1*Δ and *orm2*Δ, where all three are able to reverse the temperature-sensitive lethality of *tsc3*Δ by increasing ceramide and complex sphingolipid levels. Therefore, we propose that Dfm1 antagonizes the sphingolipid biosynthesis pathway.

The mechanism associated with Dfm1-dependent Orm2 export from the ER remains to be elucidated. Our data suggest that Dfm1 functions at the post-phosphorylation step of Orm2 where the loss of Dfm1 blocks ER export of phosphorylated Orm2. Moreover, Dfm1 does not directly function in COPII export since its absence does not

© 2022 The Authors

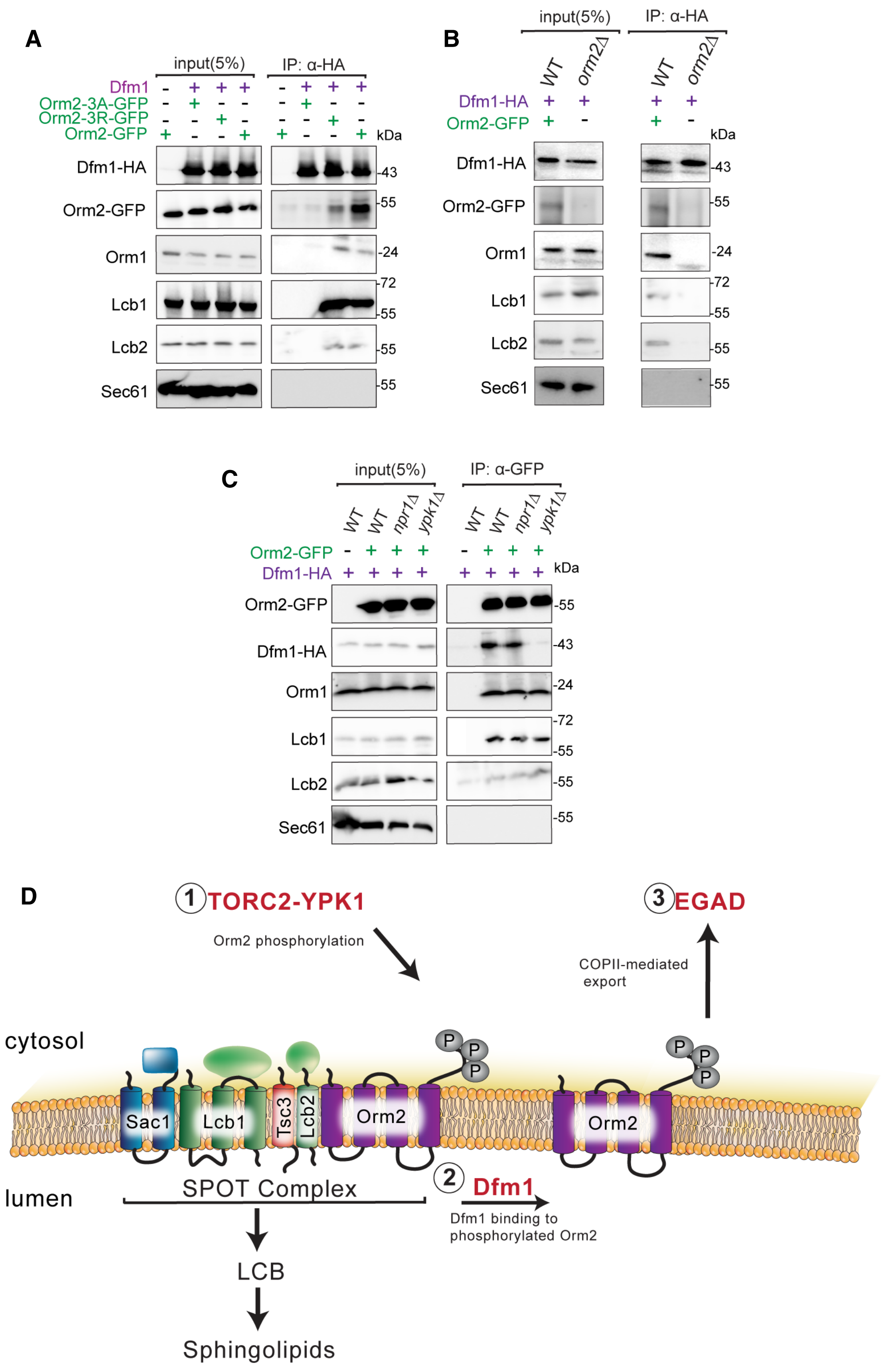

Figure 8. Dfm1 binds to phosphorylated Orm2.

A Dfm1-HA binding to Orm2-GFP, Orm2-3A-GFP, Orm2-3D-GFP, Orm1, Lcb1, and Lcb2 was analyzed by co-IP. As a negative control, cells not expressing Dfm1-HA were used. Also, Sec61 was also included to test for non-specific binding (3 biological replicates; $n = 3$).

B Same as (A), except co-IP was performed on WT and *orm2Δ* cells and Dfm1-HA binding to Orm1, Lcb1, and Lcb2 was analyzed (3 biological replicates; $n = 3$).

C Same as (A), except Orm2-GFP binding to Dfm1-HA, Orm1, Lcb1, and Lcb2 was analyzed by co-IP (3 biological replicates; $n = 3$).

D Schematic of Dfm1's role in Orm2 degradation. (i) Orm2 is inactivated via phosphorylation by the TORC2-YPK1 signaling axis. (ii) Dfm1 binds phosphorylated Orm2. (iii) Phosphorylated Orm2 is delivered to COPII vesicles. (iv) Phosphorylated Orm2 is routed to the Golgi and degraded via EGAD.

© 2022 The Authors

abrogate the export of a COPII cargo, CPY. Instead, Dfm1 interacts with Ypk1-dependent phosphorylated Orm2, which is followed by Orm2 exit from the ER and degradation by EGAD. Our laboratory has recently identified a chaperone-like Dfm1 function where it influences the solubility of aggregate-prone misfolded membrane substrates along the ER membrane (preprint: Kandel *et al*, 2022). Similar to Dfm1's function as a mediator in sphingolipid homeostasis, its chaperone-like role requires Dfm1's substrate-binding and lipid-thinning function, but not its Cdc48 recruitment function. Based on the solubility assay, where we detergent-solubilized microsomes and fractionated them into pellet (aggregated Orm2) and supernatant (solubilized Orm2), Dfm1 does not significantly enhance the solubility of Orm2 since the majority of it was already soluble (Fig EV4C). In this case, Dfm1 may not be required for influencing the solubility of Orm2, but is utilizing another chaperone-like activity to prime Orm2 for delivery to the COPII machinery for ER export (Fig 8D). The extent to which the chaperone-like activity of Dfm1 is required for Orm2 export from the ER warrants future investigations.

Based on the lipidomic analysis, *dfm1Δ* cells accumulate phosphorylated Orm2, which no longer inhibits SPT activity (Fig 7F). This was shown through a marked increase in PHS steady-state levels in *dfm1Δ* cells expressing phosphomimetic Orm2-3D. This raises the question of why the accumulation of a negative regulator would not lead to inhibition of sphingolipid biosynthesis. A possible explanation is based on a previous study in which Orms have been found to directly regulate the localization and oligomerization state of SPT at the ER in a manner that is dependent upon their phosphorylation state. Specifically, phosphorylated Orm2 shifts SPT to the monomeric state, which contributes to sustained SPT activity (Han *et al*, 2019). This model is supported by our co-IP experiment, in which phosphorylated Orm2 is still associated with the SPOTS complex (Fig 8A). In this context, the physical interaction of phosphorylated Orm2 with the SPOTS complex may have an influence on increasing SPT activity. Investigating how phosphorylated Orm2 leads to increased SPT activity will be a fruitful line of inquiry to address in the future.

The ER hosts metabolic pathways that synthesize a variety of lipids such as phospholipids, cholesterol, and sphingolipids. Thus, it is critical for the ER to sense and respond to fluctuations in lipid composition in order to maintain cellular homeostasis (Piña *et al*, 2018; Tam *et al*, 2018; Fun & Thibault, 2020). Several pathways are employed to maintain the flux of the lipid biosynthetic pathway. One such pathway is the targeting and degradation of lipid biosynthetic enzymes through ERAD-mediated degradation. For example, cholesterol synthesis is downregulated through regulated ERAD of the rate-controlling cholesterol biosynthetic enzymes Hmg-CoA reductase and squalene monooxygenase (Wangeline *et al*, 2017). Maintenance of lipid homeostasis is also critical for protein synthesis, as dysregulated lipid levels negatively impact ER protein quality control machinery. This is supported by studies demonstrating that the increased flux of sphingolipids induces UPR and sensitivity of cells to ER stress (Han *et al*, 2010). This is also in alignment with our observations where *dfm1Δorm1Δ* cells are sensitive to ER stress due to dysregulated sphingolipid metabolism (Fig EV1A). Previous studies from our lab and others have demonstrated that lipid thinning by the ERAD machinery facilitates the removal of misfolded substrates from the ER (Neal *et al*, 2018; Wu *et al*, 2020). Because lipids play a large role in ERAD retrotranslocation, fine-tuning lipid levels is critical to ensure that ERAD remains intact and functional. Our findings implicate Dfm1 as a mediator in sphingolipid homeostasis. Interestingly, by utilizing homology modeling and bioinformatic analysis, we identified sphingolipid-binding motifs on TM1 and TM6 of Dfm1, suggesting that Dfm1 may directly detect sphingolipid levels and fine-tune the control of sphingolipid production by modulating the export of Orm2 (unpublished data). Similarly, cholesterol has been shown to directly regulate levels of the E3 ligase MARCH6 levels, with increased MARCH6 levels leading to ubiquitination and degradation of the key cholesterol enzyme, squalene monooxygenase (Zelcer *et al*, 2014). Future studies on the lipid-sensing function of Dfm1 and how it de-represses SPT activity via Orm2 export from the ER will require additional experimentation.

The Orm family proteins are well conserved and all three human ORMDLs associate with SPT and directly regulate SPT activity. However, unlike their yeast counterparts, they do not appear to be phosphorylated since they lack the homologous N-terminal domains that are phosphorylated by Npr1, Ypk1, and Ypk2 in yeast. Instead, ORMDL protein levels are regulated directly by ceramide levels (Davis *et al*, 2019). Consistent with this finding, altered protein levels of ORMDLs are associated with the pathophysiology of a range of diseases, including colorectal cancer, inflammation, obesity, and diabetes (Davis *et al*, 2018). Moreover, single-nucleotide polymorphisms near ORMDL3, which lead to its increased expression, are associated with childhood asthma. Accordingly, our observation that yeast Dfm1 alters Orm2 protein levels and impacts sphingolipid metabolism raises the possibility that derlins have a causative role in these diseases. Defining the mechanism of Dfm1-mediated regulation of Orm2 levels should illuminate new treatment paradigms for patients with dysregulated sphingolipid metabolism.

In summary, we have performed proteomic analyses to enable the identification of Dfm1-interacting factors. These studies have demonstrated that several key regulators of sphingolipid biosynthesis are associated with Dfm1 and we report a novel function for Dfm1 in mediating sphingolipid homeostasis. Sphingolipids play diverse roles in cellular functions, which include cell signaling, supporting cellular structure, providing energy storage, and regulating cell growth cycles. Dysregulation of sphingolipid levels has been associated with several life-threatening disorders. Overall, this study identifies derlin rhomboid pseudoproteases as key regulators of sphingolipid levels and reveals them as potential therapeutic targets for the treatment of lipid disorders that are associated with the dysregulation of sphingolipid levels.

Material and Methods

Yeast and bacteria growth media

Standard yeast *Saccharomyces cerevisiae* growth media were used as previously described (Hampton & Rine, 1994), including yeast extract–peptone–dextrose (YPD) medium and ammonia-based synthetic complete dextrose (SC) and ammonia-based synthetic minimal dextrose (SD) medium supplemented with 2% dextrose and amino acids to enable the growth of auxotrophic strains at 30°C. *Escherichia coli* top 10 cells were grown in standard LB media with

© 2022 The Authors

ampicillin at 37°C as previously described (Gardner *et al*, 1998). HEK293 cells were cultured in DMEM medium supplemented with 10% FBS.

Plasmids and strains

Plasmids used in this study are listed in Appendix Table S1. Plasmids for this work were generated using standard molecular biological techniques (Sato *et al*, 2009) and verified by sequencing (Eton Bioscience, Inc.). Primer information is available upon request. Lcb1-RFP and Orm2-RFP plasmids were a gift from Theresa Dun (Uniformed Services University of the Health Sciences, MD). Orm2-3A-GFP and Orm2-3D-GFP plasmids were a gift from Oliver Schmidt and David Teis (Medical University of Innsbruck, Austria).

A complete list of yeast strains and their corresponding genotypes are listed in Appendix Table S2. All strains used in this work were derived from S288C or Resgen. Yeast strains were transformed with DNA or PCR fragments using the standard LiOAc method (Ito *et al*, 1983). Null alleles were generated by using PCR to amplify a selection marker flanked by 50 base pairs of the 5′ and 3′ regions, which are immediately adjacent to the coding region of the gene to be deleted. The selectable markers used for making null alleles were genes encoding resistance to G418 or CloNat/nourseothricin. After transformation, strains with drug markers were plated onto YPD followed by replica plating onto YPD plates containing (500 μg/ml G418 or 200 μg/ml nourseothricin). All gene deletions were confirmed by PCR.

Reagents

The reagents used in this study are listed in Appendix Table S3.

dfm1Δ strain handling

Due to the rapid suppression nature of *dfm1Δ* null strains, freshly transformed *dfm1Δ* null cells with the respective substrates should be used in every assay. Generation of Dfm1 mutant strains and troubleshooting guidelines are found in Bhaduri & Neal (2021).

Proximity-dependent biotinylation (BioID)

WT cells expressing either BirA-3xFlag or Dfm1-BirA-3xFlag cells were inoculated in minimal media supplemented with 1 μM biotin and 0.2% raffinose and grown overnight at 30°C. The following day, the cells were diluted to 0.2 OD_{600} and grown to log phase (0.3–0.5 OD_{600}). Once in log phase, 0.2% galactose was added to induce the expression of BirA-3xFlag and Dfm1-BirA-3xFlag. After 1 h of incubation, cells were pelleted and stored at −80°C overnight. The next day, cells were thawed and resuspended in lysis buffer (50 mM Tris–HCl pH 7.5, 150 mM NaCl, 1.5 mM $MgCl_2$, 1 mM EDTA, 0.1% SDS, 1% NP-40, 0.4% sodium deoxycholate), and 1 mM DTT supplemented with protease inhibitors: 1 mM phenylmethylsulfonyl fluoride, 260 μM 4-(2-aminoethyl) benzenesulfonyl fluoride hydrochloride, 100 μM leupeptin hemisulfate, 76 μM pepstatin A, 5 mM aminocaproic acid, 5 mM benzamidine, and 142 μM N-tosyl-L-phenylalanine chloromethyl ketone. Cells were lysed with grinding using liquid nitrogen. Crude lysate was transferred to a 1.5 ml Eppendorf tube and centrifuged for 5 min at 2,500 *g* to remove cellular debris. Clarified lysate was subjected to affinity purification with preactivated MyOne Sreptavidin C1 Magnetic Dynabeads (Invitrogen) for 4 h at 4°C using a nutator. The beads were subsequently separated from the flow-through using a magnetic stand and washed five times with cold PBS to separate the non-biotinylated proteins. Protein concentration was measured using a nanodrop, and samples (biotinylated proteins bound to magnetic beads) were submitted for mass spectrometry to analyze the biotinylated interaction partners of Dfm1.

Liquid chromatography and mass spectrometry analysis

The BioID samples were in-solution digested overnight at 37°C in 400 ng of mass spectrometry grade trypsin (Promega) enzyme. The digestion was stopped by adding formic acid to the 0.5% final concentration. The digested peptides were desalted by using C18 StageTips and were transferred to a fresh tube and then vacuum dried. The vacuum-dried peptides were resuspended in 5% formic acid/5% acetonitrile buffer and added to the vials for mass spectrometry analysis. Samples were analyzed with duplicate or triplicate injections by LC–MS–MS using EASY-nLC 1,000 liquid chromatography connected with Q-Exactive mass spectrometer (Thermo Scientific) with the following modifications. A fused silica microcapillary column (75 μm inner diameter, 15 cm) packed with C18 reverse-phase resin (ReproSil-pur 120 C18-AQ, 1.9 μm; Dr. Maisch GmbH) using an in-line nano-flow EASY-nLC 1,000 UHPLC (Thermo Scientific) was used to resolve the peptides. Peptides were eluted over a 100 min 2–30% ACN gradient, a 5 min 30–60% ACN gradient, and a 5 min 60–95% ACN gradient, with a final 10 min step at 0% ACN for a total run time of 120 min at a flow rate of 250 nl/min. All gradient mobile phases contained 0.1% formic acid. MS/MS data were collected in a data-dependent fashion using a top 10 method with a full MS mass range from 400 to 1,800 m/z, 70,000 resolutions, and an AGC target of 3e6. MS2 scans were triggered when an ion intensity threshold of 1e5 was reached with a maximum injection time of 250 ms. Peptides were fragmented using a normalized collision energy setting of 25. A dynamic exclusion time of 40 s was used, and the peptide match setting was disabled. Singly charged ions, charge states above 8, and unassigned charge states were excluded. The MS/MS spectra were searched against the UniProt *Saccharomyces cerevisiae* reference proteome database using the Maxquant software with standard settings. Statistical analysis of interactome data was carried out using differential enrichment analysis of proteomics (DEP) and Maxquant package (available online https://rdrr.io/bioc/DEP/man/DEP.html and https://www.maxquant.org/). All proteome datasets were compared to Dfm1-BioID-untreated control samples. Filter cut-offs were set at $\log_2 FC \geq 2$, *P*-value of ≤ 0.01, and at least two quantitative peptide features. These parameters were chosen in an attempt to minimize false positives while maximizing true positives. All RAW data files have been deposited and are available at MassIVE (massive.ucsd.edu) with the accession ID MSV000090333.

Spot dilution growth assay

Yeast strains were grown in YPD or minimal selection media (-Leu -Ura) supplemented with 2% dextrose to log phase (OD_{600} 0.2–0.3) at 30°C. 0.2 OD cells were pelleted and resuspended in 500 μl dH_2O. Twelve microliter of each sample was transferred to a 96-well plate

© 2022 The Authors

where a fivefold serial dilution in dH_2O of each sample was performed to obtain a gradient of 0.2–0.0000128 OD cells. The 8x12 pinning apparatus was used to pin cells onto synthetic complete (-Ura) agar plates supplemented with 2% dextrose or 2% galactose. Droplets of cells were air-dried in sterile conditions, then the plates were sealed with parafilm and incubated at 30°C. Plates were removed from the incubator for imaging after 3 days and again after 7 days.

Cycloheximide-chase assay

Cycloheximide-chase assays were performed as previously described (Sato *et al*, 2009). Cells were grown to log-phase (OD_{600} 0.2–0.03) and cycloheximide was added to a final concentration of 50 μg/ml. At each time point, a constant volume of culture was removed and lysed. Lysis was initiated with the addition of 100 μl SUME with protease inhibitors (PIs) and glass beads, followed by vortexing for 4 min. One hundred microliter of 2xUSB was added followed by incubation at 55°C for 10 min. Samples were clarified by centrifugation and analyzed by SDS–PAGE and immunoblotting.

Fluorescence microscopy

To prepare cells, overnight cultures were diluted to ~ 0.20 OD in minimal media. After growing ~ 3 h, log-phase (OD_{600} ~ 0.3–0.6) samples were pelleted and washed with dH2O. Fluorescence microscopy was accomplished using a CSU-X1 Spinning Disk (Yokogawa) confocal microscope at the Nikon Imaging Center on the UCSD campus.

Native Co-IP

Cultures from various yeast strains were grown to OD_{600} 0.2–0.45 and 15 ODs of cells were pelleted, rinsed with H_20, and lysed with 0.5 mM glass beads in 400 μl of MF buffer supplemented with protease inhibitors. This was followed by vortexing at 1 min intervals for 6–8 min at 4°C. Lysates were combined and clarified by centrifugation at 2,500 *g* for 5 min followed by centrifugation at 14,000 *g* for 15 min to obtain the microsomal pellet. The microsomal pellet was resuspended in 1 ml of Tween IP buffer (500 mM NaCl, 50 mM Tris, pH 7.5, 10 mM EDTA, and 1.5% Tween-20) and incubated on ice for 30 min. Lysates were then centrifuged for 30 min at 14,000 *g*, and the supernatant was incubated overnight with 10 μl of equilibrated GFP-Trap® agarose (ChromoTek Inc., Hauppauge, NY) at 4°C. The next day, the GFP-Trap® agarose beads were combined with one tube, washed once with non-detergent IP buffer, washed once more with IP wash buffer, and resuspended in 100 μl of 2xUSB. Samples were resolved on 8% SDS–PAGE and immunoblotted for Lcb1-RFP and Orm2-RFP α-RFP and Dfm1-GFP with α-GFP.

In vivo ubiquitination assay

Western blotting to detect *in vivo* ubiquitination was performed as described previously (Garza *et al*, 2009). Briefly, yeast strains were grown to log phase (OD600 of 0.2 to 0.3). Fifteen OD equivalents of cells were pelleted by centrifugation and resuspended in lysis buffer (0.24 M sorbitol, 1 mM EDTA, and 20 mM KH2PO4, pH 7.5) with PIs, after which 0.5 mm glass beads were added to the meniscus. The cells were lysed by vortexing in 1 min cycles at 4°C, with 1 min on ice in between, for six to eight cycles. Lysates were clarified by centrifugation at 2,500 *g* for 5 min. The clarified lysates were moved to fresh tubes, and 600 μl immunoprecipitation buffer (IPB; 15 mM Na2HPO4, 150 mM NaCl, 2% Triton X-100, 0.1% SDS, 0.5% deoxycholate, and 10 mM EDTA, pH 7.5) and 20 μl of GFP Trap (Chromotek) were added. Samples were incubated on ice for 5 min, clarified by centrifugation at 14,000 *g* for 5 min, and moved to a fresh tube. Tubes were incubated at 4°C overnight with rocking. Beads were washed twice with IPB and then washed once with IP wash buffer (50 mM NaCl and 10 mM Tris, pH 7.5). Beads were aspirated to dryness, resuspended in 55 μl 2x USB, and incubated at 65°C for 10 min. Samples were resolved by SDS–PAGE on 10% gels, transferred to nitrocellulose, and immunoblotted with monoclonal anti-ubiquitin (Fred Hutchinson Cancer Research Institute) and anti-RFP (Thermo Fisher) primary antibodies followed by goat anti-mouse (Jackson ImmunoResearch Laboratories) or goat anti-rabbit (Bio-Rad) HRP-conjugated secondary antibody.

Phosphorylation status of Orm2

Indicated strains were grown to log-phase at 30°C, pelleted, and lysed with 100 μl SUME with protease inhibitors (PIs) and glass beads, followed by vortexing for 4 min. One hundred microliter of 2xUSB was added followed by incubation at 55°C for 10 min. Samples were clarified by centrifugation, loaded onto Phos-tag precast gel (FujiFilm), and immunoblotted for Orm2 with α-GFP.

Lipid analyses

Yeast cells (1 A_{600} units) were suspended in 100 μl of extraction solution [ethanol/water/diethyl ether/pyridine/15 M ammonia (15:15:5:1:0.018, v/v)], mixed with internal standards, and incubated at 60°C for 15 min. As internal standards, four types of ceramides containing nine deuterium atoms (d_9) [N-palmitoyl(d_9)-dihydrosphingosine (d_9-C16:0 Cer-A), N-palmitoyl(d_9)-D-*ribo*-phytosphingosine (d_9-C16:0 Cer-B), N-(2′-(*R*)-hydroxypalmitoyl (d_9))-D-*erythro*-sphinganine (d_9-C16:0 Cer-B′), and N-(2′-(*R*)-hydroxypalmitoyl(d_9))-D-*ribo*-phytosphingosine (d_9-C16:0 Cer-C; all purchased from Avanti Polar Lipids, Alabaster, AL)] were used (5 pmol each). After centrifugation (2,300 *g*, room temperature, 2 min), the supernatant was recovered, and the pellets were suspended in 100 μl of extraction solution and incubated at 60°C for 15 min again. After centrifugation (2,300 *g*, room temperature, 2 min), the supernatant was recovered. The two supernatants were pooled and mixed with 700 μl of chloroform/methanol (1:2, v/v). To hydrolyze glycerolipids, alkaline treatment was performed by adding 37.5 μl of 3 M KOH and incubating at 37°C for 30 min. After neutralization by adding 22.5 μl of 5 M formic acid, the samples were sequentially mixed with 250 μl of chloroform and 250 μl of water and centrifuged (20,400 *g*, room temperature, 3 min) for phase separation. The organic phase containing lipids was recovered and dried. Lipids were resuspended in 625 μl of chloroform/methanol/water (5:4:1, v/v) and subjected to liquid chromatography (LC)-coupled tandem mass spectrometry (MS/MS) using a triple-quadrupole mass spectrometer Xevo TQ-S (Waters, Milford, MA, USA) via multiple reaction monitoring and positive-ion modes. The settings for LC separation and electrospray ionization were as

© 2022 The Authors

described previously (Ohno *et al*, 2017) and the used *m/z* values and collision energies in the MS/MS measurement were listed in Dataset EV2. Ceramides were quantified by calculating the ratio of the peak area of each ceramide species to that of the internal standard corresponding to each type of ceramide. D-type ceramides were quantified using C-type ceramide standard (d_9-C16:0 Cer-C).

Detergent solubility assay

ER microsomes were isolated by centrifuging and pelleting 15OD of yeast in log-phase growth. Pellets were resuspended in MF buffer with protease inhibitors and 0.5 mM lysis beads were added to each sample. Samples were vortexed six times in 1 min intervals, with 1 min on ice in between. Lysed cells were transferred to a new microcentrifuge tube and samples were clarified by spinning at 1,500× for 5 min at 4°C. Microsomes were separated by centrifuging clarified lysate at 14,000 *g* for 1 min. Fractions were incubated on ice in the presence or absence of 1% DDM for 1 h. The mixture was then centrifuged at 14,000 *g* for 30 min at 4°C, and the detergent soluble fraction (i.e., the supernatant) was precipitated with 20% TCA on ice for 30 min and then centrifuged at 14,000 *g* for 30 min to get a pellet of the soluble protein. Proteins from both the soluble and insoluble fractions were resuspended in the sample buffer and resolved by SDS–PAGE.

Quantification and statistical analysis

ImageJ (NIH) was used for all western blot quantifications. "Mean gray value" was set for band intensity measurements. In such experiments, a representative western blot was shown and band intensities were normalized to PGK1 loading control and quantified. t = 0 was taken as 100% and data are represented as mean ± S.E.M. from at least three experiments. GraphPad Prism was used for statistical analysis. Two-sided nested *t*-test, unpaired *t*-test, pairwise Dunnett's test, or one-way factorial ANOVA followed by Bonferroni's *post hoc* analysis was applied to compare data. Significance was indicated as follow: n.s, not significant; $*P < 0.05$, $**P < 0.01$, $***P < 0.001$, $****P < 0.0001$. The investigators were blinded during data analysis.

Data availability

The mass spectrometry proteomic data have been deposited to the Mass Spectrometry Interactive Virtual Environment (MassIVE) with the identifier MSV000090333 https://massive.ucsd.edu/ProteoSAFe/dataset.jsp?task=a163516bee184c7bbb8a87bc357df7b2. Plasmids and yeast strains generated in this study are available from our laboratory.

Expanded View for this article is available online.

Acknowledgements
We thank Peter Espenshade (Johns Hopkins Medicine), Oliver Schmidt (Medical University of Innsbruck), David Teis (Medical University of Innsbruck), Teresa Dunn (National Institutes of Health), Jim Wilhelm (University of California, San Diego), and Peter Novick (University of California, San Diego) for providing plasmids, yeast strains, and antibodies. We thank Dr. Oswald Quehenberger from the UCSD Lipidomic Core Facility for performing lipidomic analysis. We also thank Dr. Maho Niwa, Dr. David Teis, Dr. Oliver Schmidt, and the Neal lab members for in-depth discussions and technical assistance. These studies were supported by NIH grant 1R35GM133565-01, Pew Biomedical Award 34089, and NSF CAREER grant 2047391 (to S.E.N.), NIH grants 5R01GM136994-02 and DP2GM119132 (to E.J.B.), HHMI Gilliam Fellowship GT15096 (to S.E.N. and A.A.), and KAKENHI grant JPSSH04986 (to A.K.).

Author contributions
Satarupa Bhaduri: Conceptualization; data curation; formal analysis; supervision; validation; investigation; methodology; writing – original draft; writing – review and editing. **Analine Aguayo:** Conceptualization; data curation; supervision; validation; methodology; writing – original draft; writing – review and editing. **Yusuke Ohno:** Data curation; formal analysis; investigation. **Marco Proietto:** Data curation; validation; methodology. **Jasmine Jung:** Data curation; formal analysis; validation; visualization. **Isabel Wang:** Data curation; formal analysis; validation. **Rachel Kandel:** Data curation; formal analysis; validation; writing – review and editing. **Narinderbir Singh:** Data curation; formal analysis; validation. **Ikran Ibrahim:** Data curation; formal analysis; validation. **Amit Fulzele:** Data curation; validation. **Eric J Bennett:** Validation; writing – review and editing. **Akio Kihara:** Data curation; formal analysis; validation; writing – review and editing. **Sonya E Neal:** Conceptualization; data curation; validation; investigation; visualization; methodology; writing – original draft; writing – review and editing.

Disclosure and competing interest statement
The authors declare that they have no conflict of interest.

References

Bhaduri S, Neal SE (2021) Assays for studying normal versus suppressive ERAD-associated retrotranslocation pathways in yeast. *STAR Protoc* 2: 100640

Bode H, Bourquin F, Suriyanarayanan S, Wei Y, Alecu I, Othman A, Von Eckardstein A, Hornemann T (2015) HSAN1 mutations in serine palmitoyltransferase reveal a close structure-function-phenotype relationship. *Hum Mol Genet* 25: 853 – 865

Breslow DK (2013) Sphingolipid homeostasis in the endoplasmic reticulum and beyond. *Cold Spring Harb Perspect Biol* 5: a013326

Breslow DK, Collins SR, Bodenmiller B, Aebersold R, Simons K, Shevchenko A, Ejsing CS, Weissman JS (2010) Orm family proteins mediate sphingolipid homeostasis. *Nature* 463: 1048 – 1053

Carvalho P, Goder V, Rapoport TA (2006) Distinct ubiquitin-ligase complexes define convergent pathways for the degradation of ER proteins. *Cell* 126: 361 – 373

Christiano R, Arlt H, Kabatnik S, Mejhert N, Lai ZW, Farese RV, Walther TC (2020) A systematic protein turnover map for decoding protein degradation. *Cell Rep* 33: 108378

Davis D, Kannan M, Wattenberg B (2018) Orm/ORMDL proteins: gate guardians and master regulators. *Adv Biol Regul* 70: 3 – 18

Davis DL, Gable K, Suemitsu J, Dunn TM, Wattenberg BW (2019) The ORMDL/Orm–serine palmitoyltransferase (SPT) complex is directly regulated by ceramide: reconstitution of SPT regulation in isolated membranes. *J Biol Chem* 294: 5146 – 5156

Fenech EJ, Lari F, Charles PD, Fischer R, Laétitia-Thézénas M, Bagola K, Paton AW, Paton JC, Gyrd-Hansen M, Kessler BM *et al* (2020) Interaction

 © 2022 The Authors

mapping of endoplasmic reticulum ubiquitin ligases identifies modulators of innate immune signalling. *eLife* 9: e57306

Foresti O, Ruggiano A, Hannibal-Bach HK, Ejsing CS, Carvalho P (2013) Sterol homeostasis requires regulated degradation of squalene monooxygenase by the ubiquitin ligase Doa10/Teb4. *elife* 2: e00953

Foresti O, Rodriguez-Vaello V, Funaya C, Carvalho P (2014) Quality control of inner nuclear membrane proteins by the Asi complex. *Science* 346: 751 – 755

Fun XH, Thibault G (2020) Lipid bilayer stress and proteotoxic stress-induced unfolded protein response deploy divergent transcriptional and non-transcriptional programmes. *Biochim Biophys Acta Mol Cell Biol Lipids* 1865: 158449

Gable K, Slife H, Bacikova D, Monaghan E, Dunn TM (2000) Tsc3p is an 80-amino acid protein associated with serine palmitoyltransferase and required for optimal enzyme activity. *J Biol Chem* 275: 7597 – 7603

Gardner R, Cronin S, Leader B, Rine J, Hampton R, Leder B (1998) Sequence determinants for regulated degradation of yeast 3-hydroxy-3-methylglutaryl-CoA reductase, an integral endoplasmic reticulum membrane protein. *Mol Biol Cell* 9: 2611 – 2626

Garza RM, Tran PN, Hampton RY (2009) Geranylgeranyl pyrophosphate is a potent regulator of HRD-dependent 3-Hydroxy-3-methylglutaryl-CoA reductase degradation in yeast. *J Biol Chem* 284: 35368 – 35380. https://doi.org/10.1074/jbc.M109.023994

Greenblatt EJ, Olzmann JA, Kopito RR (2011) Derlin-1 is a rhomboid pseudoprotease required for the dislocation of mutant α-1 antitrypsin from the endoplasmic reticulum. *Nat Struct Mol Biol* 18: 1147 – 1152

Hampton RY, Rine J (1994) Regulated degradation of HMG-CoA reductase, an integral membrane protein of the endoplasmic reticulum, in yeast. *J Cell Biol* 125: 299 – 312

Hampton RY, Sommer T (2012) Finding the will and the way of ERAD substrate retrotranslocation. *Curr Opin Cell Biol* 24: 460 – 466

Hampton RY, Gardner RG, Rine J (1996) Role of 26 S proteasome and HRD genes in the degradation of 3-hydroxy-3-methylglutaryl-CoA reductase, an integral endoplasmic reticulum membrane protein. *Mol Biol Cell* 7: 2029 – 2044

Han S, Lone MA, Schneiter R, Chang A (2010) Orm1 and Orm2 are conserved endoplasmic reticulum membrane proteins regulating lipid homeostasis and protein quality control. *Proc Natl Acad Sci U S A* 107: 5851 – 5856

Han G, Gupta SD, Gable K, Bacikova D, Sengupta N, Somashekarappa N, Proia RL, Harmon JM, Dunn TM (2019) The ORMs interact with transmembrane domain 1 of Lcb1 and regulate serine palmitoyltransferase oligomerization, activity and localization. *Biochim Biophys Acta Mol Cell Biol Lipids* 1864: 245 – 259

Hanada K (2003) Serine palmitoyltransferase, a key enzyme of sphingolipid metabolism. *Biochim Biophys Acta Mol Cell Biol Lipids* 1632: 16 – 30

Hannun YA, Obeid LM (2018) Sphingolipids and their metabolism in physiology and disease. *Nat Rev Mol Cell Biol* 19: 175 – 191

Hiller MM, Finger A, Schweiger M, Wolf DH (1996) ER degradation of a misfolded luminal protein by the cytosolic ubiquitin-proteasome pathway. *Science* 273: 1725 – 1728

Ito H, Fukuda Y, Murata K, Kimura A (1983) Transformation of intact yeast cells treated with alkali cations. *J Bacteriol* 153: 163 – 168

Kandel R, Jung J, Syau D, Kuo T, Songster L, Aguayo A, Duttke S, Benner C, Neal S (2022) Derlin Dfm1 employs a chaperone function to resolve misfolded membrane protein stress. *bioRxiv* https://doi.org/10.1101/2022.01.25.477788 [PREPRINT]

Laney JD, Hochstrasser M (2003) Ubiquitin-dependent degradation of the yeast Matα2 repressor enables a switch in developmental state. *Genes Dev* 17: 2259 – 2270

Leto DE, Morgens DW, Zhang L, Walczak CP, Elias JE, Bassik MC, Kopito RR (2019) Genome-wide CRISPR analysis identifies substrate-specific conjugation modules in ER-associated degradation. *Mol Cell* 73: 377 – 389.e11

Mehrtash AB, Hochstrasser M (2019) Ubiquitin-dependent protein degradation at the endoplasmic reticulum and nuclear envelope. *Semin Cell Dev Biol* 93: 111 – 124

Mohassel P, Donkervoort S, Lone MA, Nalls M, Gable K, Gupta SD, Foley AR, Hu Y, Alex J, Saute M *et al* (2021) Childhood amyotrophic lateral sclerosis caused by excess sphingolipid synthesis. *Nat Med* 27: 1197 – 1204

Natarajan N, Foresti O, Wendrich K, Stein A, Carvalho P (2020) Quality control of protein complex assembly by a transmembrane recognition factor. *Mol Cell* 77: 108 – 119.e9

Neal S, Mak R, Bennett EJ, Hampton R (2017) A Cdc48 “retrochaperone” function is required for the solubility of retrotranslocated, integral membrane endoplasmic reticulum-associated degradation (ERAD-M) substrates. *J Biol Chem* 292: 3112 – 3128

Neal S, Jaeger PA, Duttke SH, Benner CK, Glass C, Ideker T, Hampton R (2018) The Dfm1 Derlin is required for ERAD Retrotranslocation of integral membrane proteins. *Mol Cell* 69: 306 – 320.e4

Nejatfard A, Wauer N, Bhaduri S, Conn A, Gourkanti S, Singh N, Kuo T, Kandel R, Amaro RE, Neal SE (2021) Derlin rhomboid pseudoproteases employ substrate engagement and lipid distortion to enable the retrotranslocation of ERAD membrane substrates. *Cell Rep* 37: 109840

Ohno Y, Kamiyama N, Nakamichi S, Kihara A (2017) PNPLA1 is a transacylase essential for the generation of the skin barrier lipid ω-O-acylceramide. *Nat Commun* 8: 14610

Piña F, Yagisawa F, Obara K, Gregerson JD, Kihara A, Niwa M (2018) Sphingolipids activate the endoplasmic reticulum stress surveillance pathway. *J Cell Biol* 217: 495 – 505

Ruggiano A, Foresti O, Carvalho P (2014) ER-associated degradation: protein quality control and beyond. *J Cell Biol* 204: 869 – 879

Sato BK, Hampton RY (2006) Yeast Derlin Dfm1 interacts with Cdc48 and functions in ER homeostasis. *Yeast* 23: 1053 – 1064

Sato BK, Schulz D, Do PH, Hampton RY (2009) Misfolded membrane proteins are specifically recognized by the transmembrane domain of the Hrd1p ubiquitin ligase. *Mol Cell* 34: 212 – 222

Schmidt O, Weyer Y, Baumann V, Widerin MA, Eising S, Angelova M, Schleiffer A, Kremser L, Lindner H, Peter M *et al* (2019) Endosome and Golgi-associated degradation (EGAD) of membrane proteins regulates sphingolipid metabolism. *EMBO J* 38: e101433

Schmidt CC, Vasic V, Stein A (2020a) Doa10 is a membrane protein retrotranslocase in er-associated protein degradation. *eLife* 9: e56945

Schmidt O, Weyer Y, Sprenger S, Widerin MA, Eising S, Baumann V, Angelova M, Loewith R, Stefan CJ, Hess MW *et al* (2020b) TOR complex 2 (TORC2) signaling and the ESCRT machinery cooperate in the protection of plasma membrane integrity in yeast. *J Biol Chem* 295: 12028 – 12044

Shimobayashi M, Oppliger W, Moes S, Jenö P, Hall MN (2013) TORC1-regulated protein kinase Npr1 phosphorylates Orm to stimulate complex sphingolipid synthesis. *Mol Biol Cell* 24: 870 – 881

Sicari D, Igbaria A, Chevet E (2019) Control of protein homeostasis in the early secretory pathway: current status and challenges. *Cell* 8: 1347

Sun Z, Brodsky JL (2019) Protein quality control in the secretory pathway. *J Cell Biol* 218: 3171 – 3187

Tam AB, Roberts LS, Chandra V, Rivera IG, Nomura DK, Forbes DJ, Niwa M (2018) The UPR activator ATF6 responds to proteotoxic and lipotoxic stress by distinct mechanisms. *Dev Cell* 46: 327 – 343.e7

© 2022 The Authors

Wang J-Z, Dehesh K (2018) ER: The silk road of interorganellar communication. *Curr Opin Plant Biol* 45: 171 – 177

Wangeline MA, Vashistha N, Hampton RY (2017) Proteostatic tactics in the strategy of sterol regulation. *Annu Rev Cell Dev Biol* 33: 467 – 489

Wu X, Siggel M, Ovchinnikov S, Mi W, Svetlov V, Nudler E, Liao M, Hummer G, Rapoport TA (2020) Structural basis of ER-associated protein degradation mediated by the Hrd1 ubiquitin ligase complex. *Science* 368: eaaz2449

Zelcer N, Sharpe LJ, Loregger A, Kristiana I, Cook ECL, Phan L, Stevenson J, Brown AJ (2014) The E3 ubiquitin ligase MARCH6 degrades squalene monooxygenase and affects 3-Hydroxy-3-methyl-Glutaryl coenzyme a reductase and the cholesterol synthesis pathway. *Mol Cell Biol* 34: 1262 – 1270

Zuzow N, Ghosh A, Leonard M, Liao J, Yang B, Bennett EJ (2018) Mapping the mammalian ribosome quality control complex interactome using proximity labeling approaches. *Mol Biol Cell* 29: 1258 – 1269

License: This is an open access article under the terms of the Creative Commons Attribution License, which permits use, distribution and reproduction in any medium, provided the original work is properly cited.

 © 2022 The Authors

Expanded View Figures

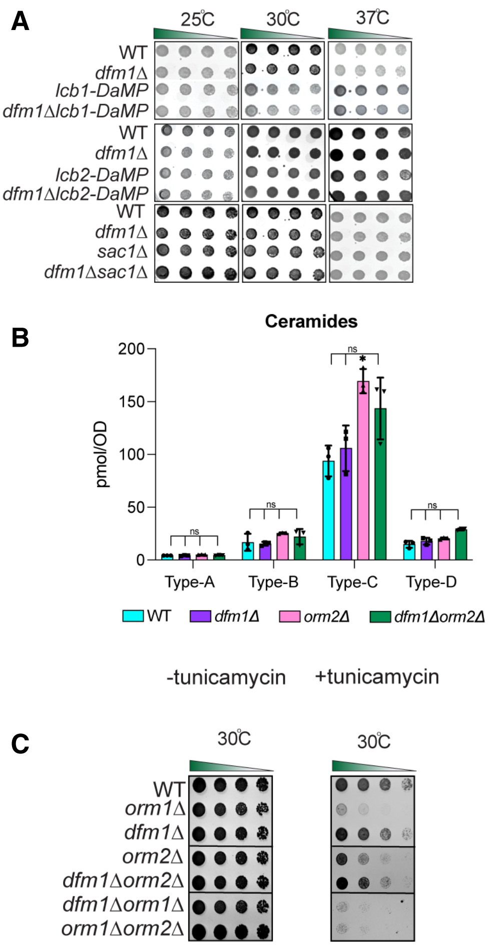

Figure EV1. Dfm1 does not genetically interact with Lcb1, Lcb2, and Sac1. Related to Fig 3.

A Indicated strains were spotted fivefold dilutions on SC plates in triplicates, and plates were incubated at room temperature, 30°C, and 37°C (3 biological replicates, 2 technical replicates; $n = 3$). *Upper panel*: WT, *dfm1Δ*, *Lcb1-DaMP*, *and dfm1ΔLcb1*-DaMP were compared for growth by dilution assay. *Middle panel*: WT, *dfm1Δ*, *Lcb2-DaMP*, *and dfm1ΔLcb2*-DaMP were compared for growth by dilution assay. *Bottom panel*: WT, *dfm1Δ*, *and dfm1Δ sac1Δ* were compared for growth by dilution assay.

B WT, *dfm1Δ*, *orm2Δ*, and *orm2Δdfm1Δ* cells were grown to log phase at 30°C and lipids were extracted and subjected to LC–MS/MS. A-, B-, C-, and D-type ceramides containing C16, C18, C20, C22, C24, and C26 fatty acids were measured (3 biological replicates; $n = 3$). Values represent the means ± S.E.M. Statistically significant differences compared to WT cells are indicated (pairwise Dunnett's test followed by Bonferroni's *post hoc* analysis; ns, non-significant, *$P < 0.05$).

C Serial dilution growth was performed on YPD plates in the presence or absence of 1 μg/ml tunicamycin using WT, *orm1Δ*, *orm2Δ*, *dfm1Δ*, *dfm1Δorm1Δ*, *orm1Δorm2Δ*, and *dfm1Δorm2Δ* cells (3 biological replicates, 2 technical replicates; $n = 3$).

© 2022 The Authors

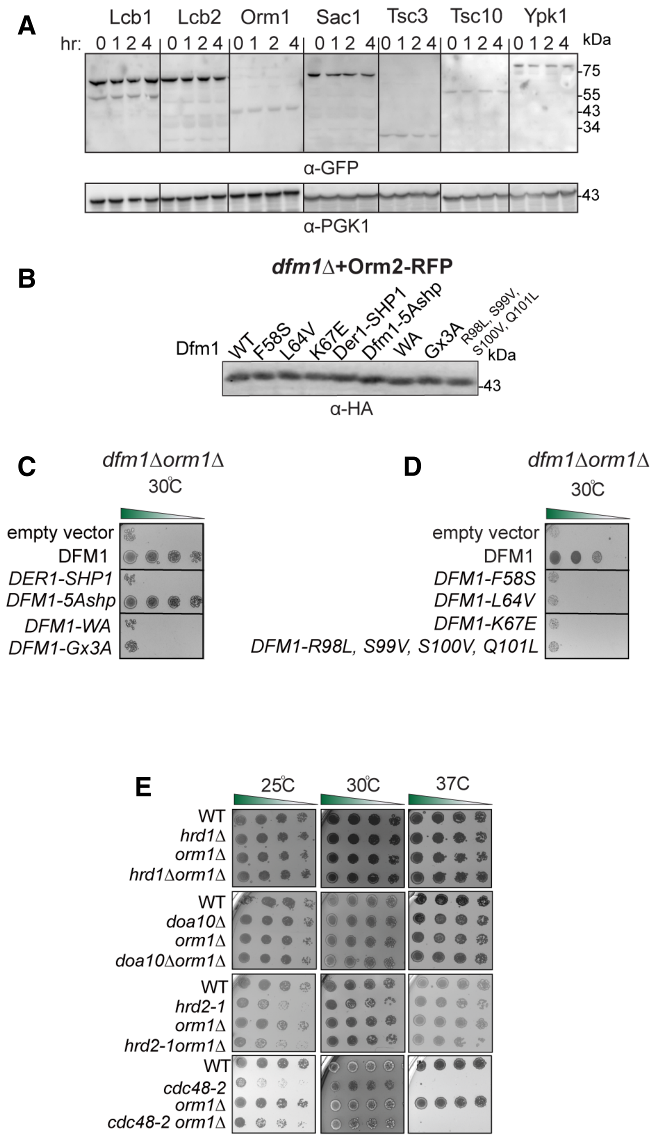

Figure EV2. Orm2 is degraded in WT strains. Related to Fig 5.

A In the indicated WT strains, degradation of Lcb1-GFP, Lcb2-GFP, Orm2-GFP, Orm2-GFP, Sac1-GFP, Tsc3-GFP, Tsc10-GFP, and Ypk1-GFP was measured by CHX-chase assay (3 biological replicates; $n = 3$). Cells were analyzed by SDS–PAGE and immunoblotted with α-GFP.

B Steady-state levels of Dfm1 and corresponding Dfm1 mutants. Cells were analyzed by SDS–PAGE and immunoblotted with α-HA.

C Serial dilution growth assay was performed on *dfm1Δorm1Δ* and strains with DFM1, DER1-SHP, DFM1-AA, DFM1-Ax3A, DFM1-5Ashpmtnt, and empty vector add back (3 biological replicates, 2 technical replicates; $n = 3$).

D Same as (C), except serial dilution growth assay was performed on *dfm1Δorm1Δ* strains with L1 mutant add back: F58S, L64V, K67E, and TMD2 quad mutant add back: DFM1-R98L, S99V, S100V, and Q101L. Indicated strains were grown on SC-Leu plates at room temperature, 30°C, and 37°C, and imaged on Day 2 and Day 7 (3 biological replicates, 2 technical replicates; $n = 3$).

E ERAD mutants do not genetically interact with *orm1Δ*. Indicated strains were spotted fivefold dilutions on SC plates in triplicate, and plates were incubated at room temperature, 30°C, and 37°C (3 biological replicates, 2 technical replicates; $n = 3$).

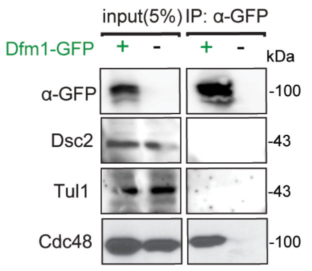

Figure EV3. Dfm1 does not interact with EGAD components. Related to Fig 6.

Dfm1-GFP binding to EGAD members, Dsc2 and Tul1, were analyzed by co-IP. As a negative control, cells not expressing Dfm1-GFP were used (2 biological replicates; $n = 2$).

© 2022 The Authors

Appendix Tables S1-S3 for An ERAD-independent role for rhomboid pseudoprotease Dfm1 in mediating sphingolipid homeostasis

Appendix Table S1-related to Methods: Plasmids used in this study, pg. 2-3

Appendix Table S2-related to Methods: Yeast strains used in this study,
pg. 4-8

Appendix Table-S3-related to Methods: Reagents used in this study, pg. 9-10

Appendix Table S1. Plasmids used in this study, Related to Methods

Plasmid	Gene		
pSN184	YIp	ADE2/ URA3	pTDH3-HMG2-GFP
pSN149	2μ	URA3	pGAL- BIRA-3xFLAG
pSN148	2μ	URA3	pGAL-DFM1-BIRA-3xFLAG
pSN246	YCp	URA3	pORM2-RFP
pSN247	YCp	LEU2	pLCB1-RFP
pSN266	YCp	URA3	pORM2-GFP
pSN267	YCp	URA3	pORM2-3D-GFP
pSN268	YCp	URA3	pORM2-3A-GFP
pSN60	YCp	LEU2	pDFM1-L64V
pSN90	YCp	LEU2	pDFM1
pSN93	YCp	LEU2	pDFM1-K67E
pSN230	YCp	LEU2	pDFM1-R98L, S99A, S100A, Q101L
pSN95	YCp	LEU2	pDFM1-F58S
pSN162	YCp	LEU2	pDFM1-5Ashp-3HA
pSN161	YCp	LEU2	pDER1-SHP-3HA
pSN164	YCp	LEU2	pDFM1-AR-3HA
pSN163	YCp	LEU2	pDFM1-Ax$_3$G-3HA
pSN269	YCp	TRP1	pORM2-RFP

pSN261	YCp	TRP1	CHS5-mCherry
pSN258	YCp	LEU2/HIS3	SEC61-mCherry
pSN252	YCp	URA3	HDEL-GFP

Appendix Table S2. Yeast strains used in this study, Related to Methods

Strain	Genotype	Reference
SEN 1	*Mata ade2-101 met2 lys-801 ura3-52 trp1::hisG leu2 his3Δ200*	This study
BY4741	*Mata met15Δ0 his3Δ1 leu2Δ0 ura3Δ0*	This study
SEN 322	*SEN1 2µ::URA3::GAL1pr-Dfm1-BIRA-FLAG*	This study
SEN 324	*SEN1 2µ::URA3::GAL1pr- BIRA-FLAG*	This study
SEN 167	*SEN1 dfm1Δ::KanMX ade2-101::ADE2::URA3::TDH3pr-HMG2-GFP 2µ::URA3::GAL1pr-Dfm1-BIRA-3xFLAG*	This study
SEN 168	*SEN1 dfm1Δ::KanMX ade2-101::ADE2::URA3::TDH3pr- HMG2-GFP 2µ::URA3::GAL1pr-BIRA-3xFLAG*	This study
SEN 169	*SEN1 dfm1Δ::KanMX ade2-101::ADE2::URA3::TDH3pr- HMG2-GFP 2µ::URA3*	This study
SEN 280	*BY4741 DFM1::DFM1-GFP::HIS*	This study
SEN 439	*BY4741 DFM1::DFM1-GFP::HIS CEN::URA3:: prORM2-RFP*	This study
SEN 438	*BY4741 DFM1::DFM1-GFP::HIS CEN::LEU2:: prLCB1-RFP*	This study
SEN 635	*SEN1 dfm1Δ::KanMX*	This study
SEN 637	*SEN1 tsc3Δ::NatR*	This study
SEN 638	*SEN1 dfm1Δ::KanMX tsc3Δ::NatR*	This study
SEN 640	*SEN1 orm1Δ::KanMX*	This study
SEN 641	*SEN1 orm1Δ::KanMX tsc3Δ::NatR*	This study
SEN 644	*SEN1 orm2Δ::KanMX*	This study
RHY 646	*SEN1 orm2Δ::KanMX tsc3Δ::NatR*	This study
SEN 285	*SEN1 dfm1Δ::KanMX orm1Δ::NatR*	This study

SEN 291	*SEN1 dfm1Δ::KanMX orm2Δ::NatR*	This study
SEN 280	*BY4741 orm1Δ::KanMX*	This study
SEN 281	*BY4741 orm2Δ::KanMX*	This study
SEN 369	*BY4741 orm1Δ::KanMX orm2Δ::LEU2*	This study
SEN 282	*BY4741 Lcb2-DaMP*	This study
SEN 266	*BY4741 dfm1Δ::KanMX Lcb2-DaMP*	This study
SEN 392	*SEN1 sac1Δ:NatR*	This study
SEN 301	*SEN1 dfm1Δ::KanMX sac1Δ:NatR*	This study
SEN 599	*SEN1 CEN::URA3:: prORM2-RFP*	This study
SEN 659	*SEN1 dfm1Δ::KanMX CEN::URA3:: prORM2-RFP*	This study
SEN 615	*SEN1 der1Δ::KanMX CEN::URA3:: prORM2-RFP*	This study
SEN 623	*SEN1 dfm1Δ::KanMX CEN::URA3:: prORM2-RFP CEN::LEU2:: prDFM1-3xHA*	This study
SEN 624	*SEN1 dfm1Δ::KanMX CEN::URA3:: prORM2-RFP CEN::LEU2*	This study
SEN 625	*SEN1 dfm1Δ::KanMX CEN::URA3:: prORM2-RFP CEN::LEU2:: prDER1-SHP1*	This study
SEN 629	*SEN1 dfm1Δ::KanMX CEN::URA3:: prORM2-RFP CEN::LEU2:: prDFM1-5Ashp*	This study
SEN 626	*SEN1 dfm1Δ::KanMX CEN::URA3:: prORM2-RFP CEN::LEU2:: prDFM1-Ax3G*	This study
SEN 628	*SEN1 dfm1Δ::KanMX CEN::URA3:: prORM2-RFP CEN::LEU2:: prDFM1-AR*	This study
SEN 635	*SEN1 dfm1Δ::KanMX CEN::URA3:: prORM2-RFP CEN::LEU2:: prDFM1-F58S*	This study
SEN 632	*SEN1 dfm1Δ::KanMX CEN::URA3:: prORM2-RFP CEN::LEU2:: prDFM1-L64V*	This study
SEN 634	*SEN1 dfm1Δ::KanMX CEN::URA3:: prORM2-RFP CEN::LEU2:: prDFM1-K67E*	This study
SEN 630	*SEN1 dfm1Δ::KanMX CEN::URA3:: prORM2-RFP CEN::LEU2:: prDFM1-R98L, S99V, S100V, Q101L*	This study
SEN 598	*SEN1 hrd1Δ::KanMX CEN::URA3:: prORM2-RFP*	This study

SEN 604	*SEN1 doa10Δ::NatR CEN::URA3:: prORM2-RFP*	This study
SEN 607	*SEN1 asi1Δ::HphMX CEN::URA3:: prORM2-RFP*	This study
SEN 622	*SEN1 tul1Δ::KanMX CEN::URA3:: prORM2-RFP*	This study
SEN 606	*SEN1 cdc48Δ::cdc48-2::NatR CEN::URA3:: prORM2-RFP*	This study
SEN 608	*SEN1 hrd2-1 CEN::URA3:: prORM2-RFP*	This study
SEN 345	*SEN1 hrd3Δ::KanMX*	This study
SEN 346	*SEN1 tsc3Δ::KanMX hrd3Δ::NatR*	This study
SEN 437	*SEN1 tsc3Δ::KanMX hrd1Δ::NatR*	This study
SEN 3	*SEN1 hrd1Δ::KanMX*	This study
SEN 652	*SEN1 doa10Δ::HphMx*	This study
SEN 654	*SEN1 doa10Δ:: HphMx tsc3Δ::KanMX*	This study
SEN 649	*SEN1 der1Δ::NatR*	This study
SEN 650	*SEN1 der1Δ::NatR tsc3Δ::KanMX*	This study
SEN 521	*BY4741 LCB1-GFP::HIS3*	This study
SEN 522	*BY4741 LCB2-GFP::HIS3*	This study
SEN 523	*BY4741 ORM1-GFP::HIS3*	This study
SEN 524	*BY4741 ORM2-GFP::HIS3*	This study
SEN 525	*BY4741 SAC1-GFP::HIS3*	This study
SEN 526	*BY4741 TSC3-GFP::HIS3*	This study
SEN 527	*BY4741 YPK1-GFP::HIS3*	This study
SEN 528	*BY4741 TSC10-GFP::HIS3*	This study
SEN 662	*SEN1 dfm1Δ::KanMX orm1Δ::NatR CEN::LEU2::prDFM1-3xHA*	This study
SEN 661	*SEN1 dfm1Δ::KanMX orm1Δ::NatR*	This study

	CEN::LEU2	
SEN 675	*SEN1 dfm1Δ::KanMX orm1Δ::NatR CEN::LEU2::prDER1-SHP*	This study
SEN 676	*SEN1 dfm1Δ::KanMX orm1Δ::NatR CEN::LEU2::prDFM1-5Ashp*	This study
SEN 677	*SEN1 dfm1Δ::KanMX orm1Δ::NatR CEN::LEU2::prDFM1-AR*	This study
SEN 678<br><br>SEN 665	*SEN1 dfm1Δ::KanMX orm1Δ::NatR CEN::LEU2::prDFM1-Ax3G*<br>*SEN1 dfm1Δ::KanMX orm1Δ::NatR CEN::LEU2::prDFM1-L64V*	This study<br><br>This study
SEN 664	*SEN1 dfm1Δ::KanMX orm1Δ::NatR CEN::LEU2::prDFM1-F58S*	This study
SEN 666	*SEN1 dfm1Δ::KanMX orm1Δ::NatR CEN::LEU2::prDFM1-K67E*	This study
SEN 667	*SEN1 dfm1Δ::KanMX orm1Δ::NatR CEN::LEU2::prDFM1-R98L, S99V, S100V, Q101L*	This study
SEN 552	*SEN1 orm1Δ::NatR*	This study
SEN 551	*SEN1 orm1Δ::NatR hrd1Δ::KanMX*	This study
SEN 543	*SEN1 hrd2-1*	This study
SEN 544	*SEN1 cdc48::cdc48-2::NatR*	This study
SEN 545	*SEN1 doa10Δ:: HphMx*	This study
SEN 546	*SEN1 hrd2-1 orm1Δ::NatR*	This study
SEN 547	*SEN1 cdc48::cdc48-2::NatR orm1Δ::KanMx*	This study
SEN 548	*SEN1 doa10Δ:: HphMx orm1Δ::NatR*	This study
SEN 671	*SEN1 orm2Δ::KanMX tsc3Δ::NatR CEN::URA3::prORM2-3R-GFP*	This study
SEN 672	*SEN1 orm2Δ::KanMX tsc3Δ::NatR CEN::URA3::prORM2-3A-GFP*	This study

SEN 673	*BY4741 orm2Δ::KanMX CEN::URA3::prORM2-3R-GFP*	This study
SEN 674	*BY4741 orm2Δ::KanMX CEN::URA3::prORM2-3A-GFP*	This study
SEN 679	*SEN1 CEN::TRP1::ORM2-RFP CEN::URA3::HDEL-GFP*	This study
SEN 680	*SEN1 dfm1Δ::KanMX CEN::TRP1::ORM2-RFP CEN::URA3::HDEL-GFP*	This study
SEN 681	*SEN1 tul1Δ::KanMX CEN::TRP1::ORM2-RFP CEN::URA3::HDEL-GFP*	This study
SEN 682	*SEN1 tul1Δ::KanMX CEN::TRP1::ORM2-RFP CEN::URA3:VPS4-GFP*	This study
SEN 683	*SEN1 CEN::URA3::ORM2-3R-GFP CEN::LEU2::HIS3::SEC61-mCherry*	This study
SEN 684	*SEN1 CEN::URA3::ORM2-3R-GFP CEN::TRP1::CHS5-mCherry*	This study
SEN 685	*SEN1 dfm1Δ::KanMX CEN::URA3::ORM2-3R-GFP CEN::LEU2::HIS3::SEC61-mCherry*	This study
SEN 686	*SEN1 dfm1Δ::KanMX CEN::URA3::ORM2-3R-GFP CEN::TRP1::CHS5-mCherry*	This study
SEN 687	*SEN1 CEN::URA3::ORM2-3A-GFP CEN::LEU2::HIS3::SEC61-mCherry*	This study
SEN 688	*dfm1Δ::KanMX CEN::URA3::ORM2-3A-GFP CEN::LEU2::HIS3::SEC61-mCherry*	This study
SEN 708	*SEN1 dfm1Δ::KanMX CEN::URA3:: prORM2-GFP CEN::LEU2:: prDFM1-3xHA*	This study
SEN 709	*SEN1 dfm1Δ::KanMX CEN::URA3:: prORM2-3D-GFP CEN::LEU2:: prDFM1-3xHA*	This study
SEN 710	*SEN1 dfm1Δ::KanMX CEN::URA3:: prORM2-3A-GFP CEN::LEU2:: prDFM1-3xHA*	This study
SEN 711	*SEN1 dfm1Δ::KanMX CEN::URA3:: prORM2-GFP CEN::LEU2*	This study
SEN 712	*SEN1 dfm1Δ::KanMX orm2Δ::NatR CEN::LEU2:: prDFM1-3xHA*	This study
SEN 713	*BY4741 ORM2-GFP::HIS3 CEN::LEU2:: prDFM1-3xHA*	This study
SEN 714	*BY4741 ORM2-GFP::HIS3 ypk1Δ::KanMX CEN::LEU2:: prDFM1-3xHA*	This study
SEN 715	*BY4741 ORM2-GFP::HIS3 npr1Δ::KanMX CEN::LEU2:: prDFM1-3xHA*	This study
SEN 716	*BY4741 CEN::LEU2:: prDFM1-3xHA*	This study

Appendix Table S3. Reagents used in this study. Related to Methods

REAGENT or RESOURCE	SOURCE	IDENTIFIER
Antibodies		
Mouse monoclonal anti-GFP	Clontech Laboratories, Inc.	Cat#632381; RRID: AB_2313808
Mouse monoclonal anti-HA	Thermo Fisher Scientific	Cat#32-6700; RRID: AB_2533092
Rabbit polyclonal anti-myc	Genscript	Cat#A00172; RRID: AB_914457
Rabbit polyclonal anti-Cdc48	Gift from Randolph Hampton	Neal et al., 2016
Mouse monoclonal anti-PGK	Thermo Fisher Scientific	Cat#459250; RRID: AB_2569747
Mouse monoclonal anti-Ubiquitin	Gift from Richard Gardner	This study
Mouse monoclonal anti-FLAG	Sigma-Aldrich	Cat# F3165 RRID:AB_259529
Rabbit Polyclonal anti-Sec61	Gift from Randolph Hampton	(Flagg et al., 2021)
Rabbit polyclonal anti-Tul1	Gift from Peter Espenshade	Tong et al., 2014
Rabbit polyclonal anti-Dsc2	Gift from Peter Espenshade	Tong et al., 2014
Rabbit polyclonal anti-Orm1	Gift from Teresa Dunn	Gable et al., 2000
Rabbit polyclonal anti-Lcb1	Gift from Teresa Dunn	Gable et al., 2000
Rabbit polyclonal anti-Lcb2	Gift from Teresa Dunn	Gable et al., 2000
**Bacterial Strain**		
Escherichia coli DH5 alpha Competent Cells	Thermo Fisher Scientific	Cat#18265017
**Chemicals, Peptides, and Recombinant Proteins**		
MG132 (benzyloxycarbonyl-Leu-Leu-aldehyde)	Sigma-Aldrich	474787; CAS: 133407-82-6
Cycloheximide	Sigma-Aldrich	C7698; CAS: 66-819
Protein A Sepharose	GE Healthcare	17-0780-01
GFP-Trap Agarose	ChromoTek	RRID: AB_2631357
RFP-Trap Agarose	ChromoTek	RRID: AB_2631362
Anti-HA Agarose	Thermo Fisher Scientific	Cat# 26181
Estreptavidina C1 Dynabeads™ MyOne™	Thermo Fisher Scientific	Cat# 65001
n-Dodecyl β-D-maltoside	Sigma-Aldrich	Cat# D4641

Primers for gene knockout		Primer name:
ATGACCGAATTAGATTATCAAGGAACTGCT GACATGGAGGCCCAGAATACCCTCCTTGAC		ORM1 For
TCAACTAATTTGGGCGCGACCTGTGATACC CAGTATAGCGACCAGCATTCACATACGATT		ORM1 Rev
ATGATTGACCGCACTAAAAACGAATCTCCA GACATGGAGGCCCAGAATACCCTCCTTGAC		ORM2 For
TTAATCTCTTTTTAAAGGATCCGGCTTGGA CAGTATAGCGACCAGCATTCACATACGATT		ORM2 Rev
ATGGCACACATCCCAGAGGTTTTACCCAAA GACATGGAGGCCCAGAATACCCTCCTTGAC		LCB1 For
TTATTTATTAGATTCTTGGCAACAGGCAAG CAGTATAGCGACCAGCATTCACATACGATT		LCB1 Rev
ATGACACAACATAAAAGCTCGATGGTGTAC GACATGGAGGCCCAGAATACCCTCCTTGAC		TSC3 For
AAGGAAGCAATACTTTAGTATACCGAAGAA CAGTATAGCGACCAGCATTCACATACGATT		TSC3 Rev
ATGGATGTTGATTCTGACGTTAATGTCTC GACATGGAGGCCCAGAATACCCTCCTTGAC		DOA10 For
TTAACTTTCATCTGGTAAATTTTCTAAAGC CAGTATAGCGACCAGCATTCACATACGATT		DOA10 Rev
ATGGTGCCAGAAAATAGAAGGAAACAGTTG GACATGGAGGCCCAGAATACCCTCCTTGAC		HRD1 For
CTAGATATGCTGGATAAATTTATCTGGTAT CAGTATAGCGACCAGCATTCACATACGATT		HRD Rev
ATGATAACACTCTTATTATACCTGTGCGTA GACATGGAGGCCCAGAATACCCTCCTTGAC		HRD3 For
TCATATGGCGAATATCTGAACATTGAAGTC CAGTATAGCGACCAGCATTCACATACGATT		HRD3 Rev
ATGGATGCTGTAATACTGAATCTCTTAGGC GACATGGAGGCCCAGAATACCCTCCTTGAC		DER1 For
TTAGGGTGTTTCAGTGTTGCGGAACCAGTC CAGTATAGCGACCAGCATTCACATACGATT		DER1 Rev
ATGTATTCTTGGAAGTCAAAGTTTAAGTTT GACATGGAGGCCCAGAATACCCTCCTTGAC		YPK1 For
CTATCTAATGCTTCTACCTTGCACCATTGA CAGTATAGCGACCAGCATTCACATACGATT		YPK1 Rev
ATGTCTTCATTAACTCGATTGCTACAGGAA GACATGGAGGCCCAGAATACCCTCCTTGAC		NPR1 For
TTATTGATTATTTTGCTTTTTCTTTTTCTT CAGTATAGCGACCAGCATT		NPR1 Rev

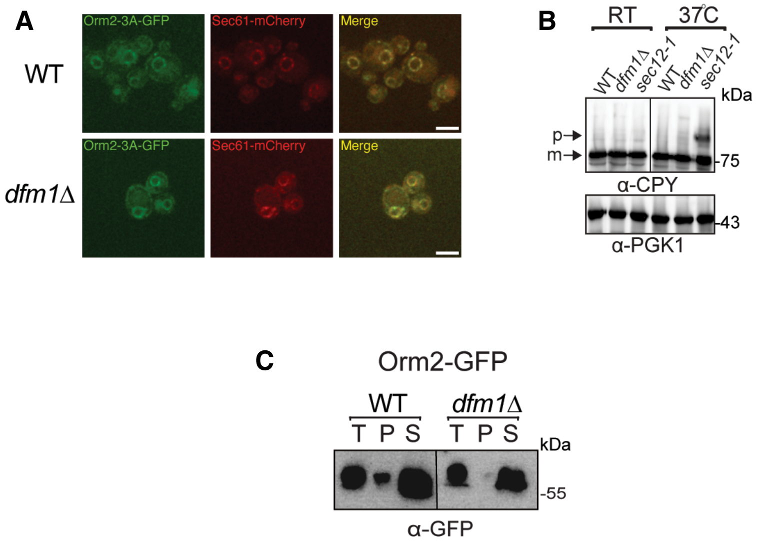

Figure EV4. Orm2-3A accumulates exclusively in the ER. Related to Fig 7.

A Fluorescence imaging was performed as in Fig 7B except for WT and *dfm1Δ* cells expressing Orm2-3A-GFP was used. Sec61-RFP (ER marker, red) was used to test for co-localization with Orm2-3A-GFP (2 biological replicates; $n = 2$). *Arrowheads* indicate Orm2 co-localizing in post-ER compartments. Scale bar, 5 μm.

B *dfm1Δ* cells do not abrogate COPII-mediated export of CPY. The indicated cells were either grown at room temperature or shifted to non-permissive growth at 37°C. Cells were analyzed by SDS–PAGE and immunoblotted for CPY with α-CPY and PGK1 with α-PGK1.

C Western blot of aggregated versus soluble Orm2-GFP at the ER. Lysates from WT and *dfm1Δ* cells containing ORM2-GFP were blotted using anti-GFP to detect Orm2. P is ER aggregated fraction and S is ER soluble fraction.

© 2022 The Authors
