# Supplementary material for: An ERAD‐independent role for rhomboid pseudoprotease Dfm1 in mediating sphingolipid homeostasis
Source: EMBO J. 2022 Nov 9;42(4):e112275. doi: 10.15252/embj.2022112275 (PMC9929635; doi:10.15252/embj.2022112275)
Supplement: Supplementary file 6 [file EMBJ-42-e112275-s006.pdf]

THE EMBO JOURNAL

An ERAD-independent role for rhomboid pseudoprotease Dfm1 in mediating sphingolipid homeostasis

Satarupa Bhaduri, Analine Aguayo, Yusuke Ohno, Marco Proietto, Jasmine Jung, Isabel Wang, Rachel Kandel, Narinderbir Singh, Ikran Ibrahim, Amit Fulzele, Eric Bennett, Akio Kihara, and Sonya Neal
DOI: 10.15252/embj.2022112275

Corresponding author(s): Sonya Neal (seneal@ucsd.edu)

Review Timeline:

Submission Date:	2nd Aug 2022
Editor correspondence:	2nd Sep 2022
Author correspondence:	7th Sep 2022
Editorial Decision:	9th Sep 2022
Revision Received:	27th Sep 2022
Accepted:	19th Oct 2022

Editor: Hartmut Vodermaier

Transaction Report:

(Note: With the exception of the correction of typographical or spelling errors that could be a source of ambiguity, letters and reports are not edited. Depending on transfer agreements, referee reports obtained elsewhere may or may not be included in this compilation. Referee reports are anonymous unless the Referee chooses to sign their reports.)

Thank you again for submitting your manuscript on ERAD-independent Dfm1 function in sphingolipid homeostasis to The EMBO Journal. We have now received the reports of three expert referees, which I am copying below for your information. As you will see, the referees acknowledge the potential interest of your findings, but are not convinced that the current mechanistic conclusions and model are decisively supported by the present data, with both alternative explanations and apparent internal discrepancies remaining to be addressed. While these conceptual concerns would currently preclude EMBO Journal publication, the reports do offer a number of constructive suggestions for deepening the insight and strengthening the main conclusions. However, since it is not clear if, and to which extent, these points might be easily addressable within the time frame of a regular revision, I would in this case consider it helpful to give you an opportunity to carefully consider all points raised together with your coworkers, and to provide a tentative point-by-point response proposing how you could envision answering the reviewers' criticisms. Based on these tentative response (parts of which we may choose to share and discuss with our referees) and possible further discussions via email or video call, we would then decide whether a major revision for The EMBO Journal would seem realistic and justified in this case, as well as whether the study might alternatively become a candidate for some of our sister journals (such as EMBO reports or Life Science Alliance) with less extensive revisions. It would be great if you could get back to me with such a response over the course of the coming two weeks.
Looking forward to hearing from you,

Referee #1 (Report for Author)

In this manuscript, Neal and coworkers describe an involvement of the rhomboid pseudoprotease Dfm1 in the regulation of sphingolipid metabolism. This is based on the observations that (1) Dfm1 physically interacts with the SPOTS complex, (2) dfm1 deletion rescues tsc3 deletion and increases flux through the sphingolipid biosynthetic pathway, and that dfm1 and orm1 interact genetically. The second part of the manuscript investigates how Dfm1 contributes to Orm2 degradation mediated by the Dsc complex in the Golgi. It is found that Dfm1 is necessary for ER export of phosphorylated Orm2. Interestingly, this activity of Dmf1 does not require Cdc48 recruitment and is independent of ubiquitination, thus establishing a retrotranslocation independent function for Dfm1.
The authors convincingly show that Dfm1 contributes to degradation of Orm2, and that this function does not rely on its "classical" retrotranslocation and Cdc48 recruitment function but on promoting ER exit (but see point 3 below). However, no convincing mechanistic model is provided how this is linked to the other observation, that dfm1 deletion in conjunction with tsc3 deletion phenocopies double deletions of orm1/2 and tsc3.
My main concern is that the discrepancy between, (1) lack of Orms restores viability through increased SPOTS activity, but (2) increased stability of Orm2 in the absence of dfm1 has a similar phenotype, is not resolved. Other possible mechanisms by which Dfm1 might act on sphingolipid metabolism, as suggested by variable effects on different ceramide classes, are not explored. The fact that orm deletions are potent activators of ER stress (Han et al, PNAS, 2010) und potentially cross-talk with Dfm1 in this way, is not explored. In my view, these limitations reduce the overall impact of the study. The finding of a retrotranslocation-independent mode of action for Dfm1 is certainly of interest for the membrane protein quality control field. However, this presumably chaperone-like activity of Dfm1 has also been explored by the Neal laboratory in a recent biorxiv paper (Bhaduri et al, 2022). Under what circumstances Dfm1 interacts with phosphorylated Orm2 (still part of the SPOTS complex, a rearranged spots complex, or dissociated Orm2) is not explored.

Other major experimental concerns:

- Growth assays using serial dilutions of cultures on plates are used to evaluate the effect of mutations. The quality of these experiments is not very high. In many cases, no effect of dilution is discernable.
- BioID, coIPs, and colocalization are used to establish interaction of Dfm1 with the SPOTS complex. Each of these methods has its short-coming. BioID e.g., relies on strong overexpression of Dfm1. Co-IPs of membrane proteins are tricky, especially if both proteins interrogated are relatively abundant and reside in the same membrane. Experiments in Fig. 2 would thus benefit from some control of other abundant ER proteins that should not coIP with the SPOTS complex or Dfm1. Does the interaction rely on the presence of phosphorylated Orm2? This could be tested using the phosphomimetic or phosphorylation defective Orm2 mutants. Does Dfm1 only interact with the SPOTS complex through Orm2?
- In Fig. 4, deletion of orm2 or double deletion of both Orms are shown to result in increased ceramide levels. Han et al. (PNAS, 2010, Figure S6) reported a decrease of ceramides under these conditions, while LCBs were increased. Please comment.
- The claim that Dfm1 acts on Orm2 independent of its Cdc48-recruitment is largely based on Fig. 5D and the comparison of stability in the presence of wt Dfm1 and the 5Ashp mutant. In this particular figure however, degradation with wt Dfm1 is considerably impaired compared to those shown in 5A and 5G. Steady state levels too do not appear to be much affected. . Please comment. In the same figure, expression levels of the different dfm1 mutants should be shown, to exclude that defects are merely due to reduced Dfm1 levels.

Minor comments:

- p.2/3 Paragraph 2 of the introduction lacks a statement that the situation in S. cerevisiae is described here, e.g. no mention of the increased repertoire of E3 ligases involved in ERAD-M in animals, or the fact that the Asi complex only exist in fungi.
- Methods: Description of how BioID experiments in incomplete. In which buffer were the cells lysed, especially which detergent? Which strain was used? Table S2 suggests that this was done in SEN1 (which contains a DMF1 copy), whereas Fig. 1C used a dfm1 KO (although that strain is not listed in table S2).
- Figure legend to Fig. 1: panel enumeration is wrong. Second "D" should be "E", "E" should be "F".
- Fig. 1D: Why is Cdc48 signal missing from flow-through samples? It's a very abundant protein.
- Ref. 19 is incomplete.
- Methods section lacks information on how experiments relying on Phos-tag technology were performed.
- Several typos in Fig. 5: In 5D, it should be "Orm2-RFP" instead of "Hmg2-GFP", in 5G it should be "Orm2-RFP" instead of "Orm2-GFP".
- Table S4 is impossible to evaluate because column labels are not legible.

Referee #2 (Report for Author)

In their manuscript, Bhaduri et al. report that the ER membrane protein Dfm1 physically interacts with the SPOTS complex which catalyzes the first step in sphingolipid synthesis. Using genetic and biochemical experiments, the authors show that dfm1Δ cells have increased ceramides, similar to orm1 and orm2 cells lacking negative regulators of sphingolipid synthesis. The authors show that Orm2 protein is stabilized in dfm1Δ cells; although Dfm1 plays a role in ERAD, the effect of dfm1Δ on Orm2 is through EGAD. In their model, the authors suggest Dfm1 promotes export of Orm2 from the ER for degradation by EGAD in post-ER compartments. The manuscript is well-written and suggests a novel function for Dfm1.

To support their model in Fig. 8, the authors show genetic evidence that Orm2-3A lacking Ypk1 phosphorylation is unable to undergo EGAD, i.e. Fig 7D shows that orm2-3D does not affect growth of orm2Δ tsc3Δ cells while orm2-3A reverses the suppression seen in tsc3Δ orm2Δ cells. Because the interpretation of the genetic result is indirect and complicated, the authors should show, more to the point, that Ypk1 is required for Orm2 interaction with Dfm1. Will Orm2 lacking Ypk1 phosphorylation sites (Orm2-3A) still interact with Dfm1? In this way, the model will be better supported and the manuscript improved. It is also of interest to determine whether Ypk1-mediated phosphorylation abrogates Orm2 association with SPOTS components while Npr1-mediated phosphorylation may not.

Minor corrections:
The Han et al. reference is incompletely listed.
At the top of page 21, one of the references to orm1Δ should be changed to orm2Δ.

Referee #3 (Report for Author)

In this manuscript, Bhaduri, Aguayo and colleagues report a new function for the ERAD component Dfm1. Using unbiased protein-proximity labeling, they identify interactions with components of the SPOTS complex that functions in the first step of sphingolipid synthesis. Using genetics, they provide strong evidence that DFM1 interacts genetically with key regulators of the Lcb1/Lcb2 (SPT) enzyme and that lipid homeostasis is perturbed significantly in double mutants. Mechanistically, the authors determine that Dfm1 functions as a positive regulator of SPT by promoting the ER exit of the negative regulator Orm2. This novel function for Dfm1 requires its pseudo-rhomboid domain that interacts with ERAD substrates, but not the SHP boxes required for ERAD, supporting a non-ERAD function for Dfm1. The manuscript is very clearly written, and experiments are rigorously performed and support the conclusions. Suggestions are provided below to strengthen the conclusions and to increase the impact of the findings. Given the role of Dfm1 in ERAD, it is particularly important to show that the effects of Dfm1 deletion are direct and not through changes in the levels of another Dfm1-dependent ERAD substrate.

Major comments:
1. To demonstrate specificity, Fig. 2A IP should include an ER membrane protein that is not detected by BioID as a negative control, especially since the detergent solubilized membranes were cleared at 14,000xg. Dfm1-GFP would not be expected to pull down Sec61, for example.

2. The authors propose that Dfm1 acts directly on Orm2 through its membrane domain. To further support this model, the authors should test whether mutation of the pseudo-rhomboid disrupts the protein-protein interaction observed in Fig. 2A.

3. The authors demonstrate that Orm2 accumulates in the ER in dfm1D cells and is protected from degradation due to its inability to access Tul1 E3 ligase. The results in Fig 6C indicate that Orm2 is ubiquitinylated at wild-type levels in dfm1D cells indicating that it has access to Tul1. How do the authors reconcile this observation with the model that Dfm1 promotes ER exit? Could another ERAD E3 ligase act on Orm2 in dfm1D cells?

4. Lipidomic analysis of dfm1D cells suggests that DFM1 plays a relatively minor role in control of sphingolipid homeostasis. The only lipid defect observed in dfm1D cells alone is the accumulation of C18-PHS. Other defects are observed in combination with tsc3D or orm1D. The most significant

finding presented is that Dfm1 functions outside of ERAD (or at least SHP box function) to promote ER exit of Orm2, but mechanism is not explored. To confirm that the defect is at ER exit, the authors should test the requirement for DFM1 in an in vitro vesicle budding assay.

Minor comments:
1. Why did the authors test interactions of Dfm1 with Lcb1 and Orm2 when Lcb2 and Orm1 were identified in the BioID experiment?

2. Please denote Lcb1 and Orm2 on the volcano plot for completeness.

3. Page 8, line 1, the statement "Hence, Dfm1 binds directly to SPOTS complex members at the ER." is not supported by the data, which would require an in vitro binding experiment.

UNIVERSITY OF CALIFORNIA, SAN DIEGO

BERKELEY • DAVIS • IRVINE • LOS ANGELES • MERCED • RIVERSIDE • SAN DIEGO • SAN FRANCISCO SANTA BARBARA • SANTA CRUZ

Sonya E. Neal, Ph. D.
Assistant Professor of Biological Sciences
Cell and Developmental Biology
Division of Biological Sciences
4401 Tata Hall
La Jolla, CA 92093

Mobile: (323)-303-4845
seneal@ucsd.edu

September 7, 2022

Dear Dr. Hartmut Vodermaier,

Please find our tentative response to the Bhaduri and Aguayo et al. paper "An ERAD-independent role for rhomboid pseudoprotease Dfm1 in mediating sphingolipid homeostasis." We have included some recent results, which addresses major concerns of all three reviewers, including plans to address issues of reportage, analysis, and discussion. We would like to thank the reviewers for their thorough, incisive, and detailed critiques of the work. Their suggestions will make the manuscript much better in many ways, from the detailed level of data presentation all the way up to the conceptual level of thinking about how derlin Dfm1 employs an ERAD-independent role for regulating sphingolipid metabolism. Below, we address in detail each point made by each reviewer, and hope that our response is satisfactory to proceed with the the next step of our story. The **black text** is verbatim from the reviewer and the **blue text** is our response. Thank you!

Referee #1

In this manuscript, Neal and coworkers describe an involvement of the rhomboid pseudoprotease Dfm1 in the regulation of sphingolipid metabolism. This is based on the observations that (1) Dfm1 physically interacts with the SPOTS complex, (2) dfm1 deletion rescues tsc3 deletion and increases flux through the sphingolipid biosynthetic pathway, and that dfm1 and orm1 interact genetically. The second part of the manuscript investigates how Dfm1 contributes to Orm2 degradation mediated by the Dsc complex in the Golgi. It is found that Dfm1 is necessary for ER export of phosphorylated Orm2. Interestingly, this activity of Dmf1 does not require Cdc48 recruitment and is independent of ubiquitination, thus establishing a retrotranslocation independent function for Dfm1.

The authors convincingly show that Dfm1 contributes to degradation of Orm2, and that this function does not rely on its "classical" retrotranslocation and Cdc48 recruitment function but on promoting ER exit (but see point 3 below).

- “However, no convincing mechanistic model is provided how this is linked to the other observation, that dfm1 deletion in conjunction with tsc3 deletion phenocopies double deletions of orm1/2 and tsc3. My main concern is that the discrepancy between, (1) lack of Orms restores viability through increased SPOTS activity, but (2) increased stability of Orm2 in the absence of dfm1 has a similar phenotype, is not resolved. “

Thank you for pointing out this conundrum. We do agree these are important observations that needs to be reconciled. We analyzed this further by measuring PHS levels in WT or *dfm1Δ* cells either expressing phospho-null Orm2-3A or phosphomimic Orm2-3D. As expected, in both WT and *dfm1Δ* cells, Orm2-3A (Orm2 version accumulating in the ER and not degraded by EGAD) leads to low levels of PHS whereas WT cells + Orm2-3D (phosphomimic Orm2 constitutively degraded by EGAD) leads to significantly higher levels of PHS. Remarkably, *dfm1Δ* cells +Orm2-3D (phosphomimic Orm2 accumulating in the ER) leads to significantly higher levels of PHS. **This finding suggests accumulation of phosphorylated Orm2 at the ER, increases SPT activity, which phenocopy cells lacking orms1/2 where SPT activity is no longer inhibited.**

In the future, we are interested in precisely determining how stabilized phosphorylated Orm2 at the ER leads to increased SPT activity. We plan to flesh out this new line of inquiry in the discussion section.

- “Other possible mechanisms by which Dfm1 might act on sphingolipid metabolism, as suggested by variable effects on different ceramide classes, are not explored.”

Thank you for pointing this out. Based on *dfm1Δorm1Δ* cells having variable effects on different ceramide classes, we hypothesize that Dfm1 modulates another enzyme(s) involved in ceramide synthesis. This is an exciting result, which paves the way for identifying an additional role for Dfm1 in regulating sphingolipid biosynthesis. We believe this line of inquiry is outside the scope of this study since we are solely focused on deciphering Dfm1’s novel role in regulating Orm2. We believe several more papers can be borne out from studying its effect on different classes of ceramides

- The fact that orm deletions are potent activators of ER stress (Han et al, PNAS, 2010) und potentially cross-talk with Dfm1 in this way, is not explored. In my view, these limitations reduce the overall impact of the study.

Thank you so much for this suggestion. Given Dfm1's role in mediating ERAD, we have indeed considered how Dfm1 cross-talks with sphingolipid metabolism and ER stress.

Previously, Han et al, PNAS demonstrated that dysregulated sphingolipid metabolism through double deletion of *orm1Δorm2Δ* triggers ER stress and mounts UPR. Recently, we have also tested whether *orm1Δdfm1Δ* activates UPR. **We have successfully introduced a UPR fluorescent reporter in each strain, and found that *dfm1Δ*, *orm1Δ*, and *orm2Δ* alone doesn't mount UPR whereas *orm1Δdfm1Δ* activate UPR, which phenocopies the previous observation with *orm1Δorm2Δ* cells.** These findings further strengthen our claim that sphingolipid homeostasis is dysregulated in *orm1Δdfm1Δ* cells, and we plan to include this new data in our revised manuscript. Moreover, we are at the tip of the iceberg with understanding how Dfm1 potentially serves as a liaison between sphingolipid metabolism and ER stress. We believe further efforts in this arena are outside the scope of this particular study and is a future line of inquiry we wish to address.

- "The finding of a retrotranslocation-independent mode of action for Dfm1 is certainly of interest for the membrane protein quality control field. However, this presumably chaperone-like activity of Dfm1 has also been explored by the Neal laboratory in a recent biorxiv paper (Bhaduri et al, 2022). Under what circumstances Dfm1 interacts with phosphorylated Orm2 (still part of the SPOTS complex, a rearranged spots complex, or dissociated Orm2) is not explored."

Thank you for pointing this out. We have supporting evidence demonstrating Dfm1's role in sphingolipid metabolism is completely new and independent from both its ERAD retrotranslocation function (Figure 5, this manuscript) and solubilization function (recently identified in our preprint (Kandel et al., 2022)).

To test if Dfm1 is required for solubilizing Orm2, we employed our solubilization assay and showed that Dfm1 addback doesn't lead to marked increase in solubilized Orm2. Hence, Dfm1 is employing an entirely new function in sphingolipid metabolism. We believe this novel role serves as a major conceptual advancement in several fields such as membrane protein quality control, membrane trafficking, and sphingolipid metabolism

In regard to studying Dfm1's interaction with phosphorylated Orm2, we addressed this in a similar question asked below by adding in several new co-IP experiments.

- "Other major experimental concerns:
  - Growth assays using serial dilutions of cultures on plates are used to evaluate the effect of mutations. The quality of these experiments is not very high. In many cases, no effect of dilution is discernable. "

For initial submission, we had to upload very low quality dilution assay images to keep the file size at a minimum. We will swap in high quality images where dilution effect is discernable.

- "BioID, coIPs, and colocalization are used to establish interaction of Dfm1 with the SPOTS complex. Each of these methods has its short-coming. BioID e.g., relies on strong overexpression of Dfm1. Co-IPs of membrane proteins are tricky, especially if both proteins interrogated are relatively abundant and reside in the same membrane. Experiments in Fig. 2 would thus benefit from some control of other abundant ER proteins that should not coIP with the SPOTS complex or Dfm1."

Thank you. We do agree this is a major short-coming of BioID and coIPs and plan to include Sec61 as a negative control.

- "Does the interaction rely on the presence of phosphorylated Orm2? This could be tested using the phosphomimetic or phosphorylation defective Orm2 mutants. Does Dfm1 only interact with the SPOTS complex through Orm2?"

We are pleased with your advice on these forward-thinking experiments. In fact, we have already begun to rigorously test this. We have included results from our latest experiments where Dfm1 specifically interacts with Orm2-3D, but not Orm2-3A. Interestingly, with Orm2-3A, Dfm1 no longer associates with additional SPOT complex members (Orm1, Lcb1, and Lcb2). This implies, Dfm1 interacts with SPOT complex members through phosphorylated Orm2. We further confirmed this with co-IP in *orm2*Δ cell which prevents Dfm1's association with Orm1, Lcb1, and Lcb2.

- In Fig. 4, deletion of orm2 or double deletion of both Orms are shown to result in increased ceramide levels. Han et al. (PNAS, 2010, Figure S6) reported a decrease of ceramides under these conditions, while LCBs were increased. Please comment.

  We have also noted this discrepancy. There were also two seminal studies(Breslow et al., 2010) (Shimobayashi et al., 2013), which are in agreement with our findings that lack of orms increases ceramide levels. We believe these differences could be a result of yeast growth conditions used prior to analysis (i.e-growth media, growth phase that yeasts were harvested).

- "The claim that Dfm1 acts on Orm2 independent of its Cdc48-recruitment is largely based on Fig. 5D and the comparison of stability in the presence of wt Dfm1 and the 5Ashp mutant. In this particular figure however, degradation with wt Dfm1 is considerably impaired compared to those shown in 5A and 5G. Steady state levels too do not appear to be much affected. Please comment."

  Thank you for pointing this out. This particular replicate does not show an effect on Orm2 steady-state levels. We do have two other replicates where there is a decrease in steady-state levels with both WT and Dfm1-5Ashp mutants. We will include this in the newly revised manuscript.

  In the same figure, expression levels of the different dfm1 mutants should be shown, to exclude that defects are merely due to reduced Dfm1 levels."

  We are grateful that you noticed this important data missing in our manuscript. We plan to assess steady-state levels of each mutant in the corresponding strains and will incorporate this into the revised manuscript. We do expect expression levels of each mutant to be at

similar levels as wild-type Dfm1 since this was rigorously tested in our previous publications (Neal et al., 2018; Nejatfard et al., 2021)

Minor comments:

- p.2/3 Paragraph 2 of the introduction lacks a statement that the situation in S. cerevisiae is described here, e.g. no mention of the increased repertoire of E3 ligases involved in ERAD-M in animals, or the fact that the Asi complex only exist in fungi.
- Methods: Description of how BioID experiments in incomplete. In which buffer were the cells lysed, especially which detergent? Which strain was used? Table S2 suggests that this was done in SEN1 (which contains a DMF1 copy), whereas Fig. 1C used a dfm1 KO (although that strain is not listed in table S2).
- Figure legend to Fig. 1: panel enumeration is wrong. Second "D" should be "E", "E" should be "F".
- Fig. 1D: Why is Cdc48 signal missing from flow-through samples? It's a very abundant protein.
- Ref. 19 is incomplete.
- Methods section lacks information on how experiments relying on Phos-tag technology were performed.
- Several typos in Fig. 5: In 5D, it should be "Orm2-RFP" instead of "Hmg2-GFP", in 5G it should be "Orm2-RFP" instead of "Orm2-GFP".
- Table S4 is impossible to evaluate because column labels are not legible.

All minor comments will be addressed an incorporated into the revised manuscript.

Referee #2

In their manuscript, Bhaduri et al. report that the ER membrane protein Dfm1 physically interacts with the SPOTS complex which catalyzes the first step in sphingolipid synthesis. Using genetic and biochemical experiments, the authors show that dfm1Δ cells have increased ceramides, similar to orm1 and orm2 cells lacking negative regulators of sphingolipid synthesis. The authors show that Orm2 protein is stabilized in dfm1Δ cells; although Dfm1 plays a role in ERAD, the effect of dfm1Δ on Orm2 is through EGAD. In their model, the authors suggest Dfm1 promotes export of Orm2 from the ER for degradation by EGAD in post-ER compartments. The manuscript is well-written and suggests a novel function for Dfm1.To support their model in Fig. 8, the authors show genetic evidence that Orm2-3A lacking Ypk1 phosphorylation is unable to undergo EGAD, i.e. Fig 7D shows that orm2-3D does not affect growth of orm2Δ tsc3Δ cells while orm2-3A reverses the suppression seen in tsc3Δ orm2Δ cells.

Because the interpretation of the genetic result is indirect and complicated, the authors should show, more to the point, that Ypk1 is required for Orm2 interaction with Dfm1. Will Orm2 lacking Ypk1 phosphorylation sites (Orm2-3A) still interact with Dfm1? In this way, the model will be better supported and the manuscript improved. It is also of interest to determine whether Ypk1-mediated phosphorylation abrogates Orm2 association with SPOTS components while

Npr1-mediated phosphorylation may not.

We are pleased to see this feedback since we immediately set out to test this. We have included the co-IP experiments above demonstrating that Dfm1 specifically interacts with Orm2-3D, but not Orm2-3A. Interestingly, with Orm2-3A, Dfm1 no longer associates with Orm2 along with other SPOTS complex members (Orm1, Lcb1, and Lcb2).

We were also interested in investigating the effect of Ypk1-mediated vs. Npr1-mediated Orm2 phosphorylation on Dfm1 binding. Interestingly, in cells devoid of Npr1, Orm2 still associated with Dfm1whereas in cells devoid of Ypk1, Orm2 no longer associates with Dfm1. Interestingly, in cells lacking either Npr1 or Ypk1, Orm2 still associates with SPOT complex members. We will include these exciting new results into the revised manuscript.

Minor corrections:
The Han et al. reference is incompletely listed.
At the top of page 21, one of the references to orm1Δ should be changed to orm2Δ.

We will address and incorporate all minor comments into revised manuscript.

Referee #3 (Report for Author)

In this manuscript, Bhaduri, Aguayo and colleagues report a new function for the ERAD component Dfm1. Using unbiased protein-proximity labeling, they identify interactions with components of the SPOTS complex that functions in the first step of sphingolipid synthesis. Using genetics, they provide strong evidence that DFM1 interacts genetically with key regulators of the Lcb1/Lcb2 (SPT) enzyme and that lipid homeostasis is perturbed significantly in double mutants. Mechanistically, the authors determine that Dfm1 functions as a positive regulator of SPT by promoting the ER exit of the negative regulator Orm2. This novel function for Dfm1

requires its pseudo-rhomboid domain that interacts with ERAD substrates, but not the SHP boxes required for ERAD, supporting a non-ERAD function for Dfm1. The manuscript is very clearly written, and experiments are rigorously performed and support the conclusions. Suggestions are provided below to strengthen the conclusions and to increase the impact of the findings.

"Given the role of Dfm1 in ERAD, it is particularly important to show that the effects of Dfm1 deletion are direct and not through changes in the levels of another Dfm1-dependent ERAD substrate. "

Thank you so much for this important feedback. We do agree with the possibility of dealing with pleiotropic effects associated with Dfm1 deletion, given its role in ERAD.

Our newly-acquired co-IP results demonstrates the specificity of Dfm1's effect in which it controls Orm2 ER exit by specifically binding to phosphorylated Orm2.

Major comments:
1. To demonstrate specificity, Fig. 2A IP should include an ER membrane protein that is not detected by BioID as a negative control, especially since the detergent solubilized membranes were cleared at 14,000xg. Dfm1-GFP would not be expected to pull down Sec61, for example.

Yes, we do agree with the shortcomings of BioID experiments and plan to include a Sec61 negative control to further validate that Dfm1 interactions are specific.

2. The authors propose that Dfm1 acts directly on Orm2 through its membrane domain. To further support this model, the authors should test whether mutation of the pseudo-rhomboid disrupts the protein-protein interaction observed in Fig. 2A.

We are grateful for this suggestion since this was one of the ongoing experiments being done in the lab. Specifically, we demonstrate that rhomboid mutants we previously characterized are important for substrate targeting, are also important for Orm2 binding.

3. The authors demonstrate that Orm2 accumulates in the ER in dfm1D cells and is protected from degradation due to its inability to access Tul1 E3 ligase. The results in Fig 6C indicate that Orm2 is ubiquitinylated at wild-type levels in dfm1D cells indicating that it has access to Tul1. How do the authors reconcile this observation with the model that Dfm1 promotes ER exit? Could another ERAD E3 ligase act on Orm2 in dfm1D cells?

Thank you so much for pointing this out. We have previously studied the nature of dfm1Δ cells and found that these cells are inherently unstable and rapidly suppressed by Hrd1(Neal et al., 2020). In other words, when dfm1 is deleted, additional copy of Hrd1 arise via aneuploidy and can compensate for lack of Dfm1 function. We believe in this particular case, Hrd1 is taking over and ubiquitinating Orm2. To address this, we plan to look at Orm2 ubiquitination status in dfm1Δhrd1Δ. If Hrd1 is indeed compensating for lack of Dfm1 function, Orm2 shouldn't be ubiquitinated in dfm1Δhrd1Δ double-null.

4. Lipidomic analysis of dfm1D cells suggests that DFM1 plays a relatively minor role in control of sphingolipid homeostasis. The only lipid defect observed in dfm1D cells alone is the accumulation of C18-PHS. Other defects are observed in combination with tsc3D or orm1D.

Initially, we were also surprised that dfm1Δ alone didn't have a significant effect on sphingolipid levels. However, from previous studies and our observations alone, regulators of sphingolipid homeostasis are redundant and pleiotropic in nature. For example, most characterized negative regulators of SPT, orm1 or orm2 deletion alone, also has little effect on sphingolipid homeostasis (Breslow et al., 2010; Han et al., 2010.). This is what we see with dfm1-null, where we see a major effect with double-nulls.

The most significant finding presented is that Dfm1 functions outside of ERAD (or at least SHP box function) to promote ER exit of Orm2, but mechanism is not explored. To confirm that the defect is at ER exit, the authors should test the requirement for DFM1 in an in vitro vesicle budding assay.

Thank you for pointing this out. We believe Dfm1 functions upstream of ER exit for the reason as follow:

We did not observe a general defect in COPII-mediated export in dfm1Δ cells (Fig. S4 B) and believe Dfm1 doesn't function directly in ER exit (also supported by no enrichment with COPII machineries in BioID experiment), but functions directly with the SPOTS complex in priming Orm2 for ER exit. This is supported by our new co-IP data, where we demonstrate that Dfm1 engages with phosphorylated Orm2 and not the phospho-null version of Orm2 (Orm2-3A).

Minor comments:
1. Why did the authors test interactions of Dfm1 with Lcb1 and Orm2 when Lcb2 and Orm1 were identified in the BioID experiment?

We have included Lcb2 and Orm1 in our co-IP experiments.

2. Please denote Lcb1 and Orm2 on the volcano plot for completeness.
We will incorporate this in our revised manuscript.

3. Page 8, line 1, the statement "Hence, Dfm1 binds directly to SPOTS complex members at the ER." is not supported by the data, which would require an in vitro binding experiment.

Thank you. We performed co-IP in orm2-null cells and show that Dfm1 no longer associates with the SPOT complex. Our data implicates that Dfm1 interacts with SPOTs complex through its interaction with Orm2. We will revise this statement accordingly.

1. Breslow, D.K., Collins, S.R., Bodenmiller, B., Aebersold, R., Simons, K., Shevchenko, A., Ejsing, C.S., Weissman, J.S., 2010. Orm family proteins mediate sphingolipid homeostasis. Nature 463, 1048–1053. doi:10.1038/NATURE08787
2. Han, S., Lone, M.A., Schneiter, R., Chang, A., n.d. Orm1 and Orm2 are conserved endoplasmic reticulum membrane proteins regulating lipid homeostasis and protein quality control. doi:10.1073/pnas.0911617107
3. Neal, S., Jaeger, P.A., Duttke, S.H., Benner, C.K., Glass, C., Ideker, T., Hampton, R., 2018. The Dfm1 Derlin Is Required for ERAD Retrotranslocation of Integral Membrane Proteins. Mol. Cell 69. doi:10.1016/j.molcel.2017.12.012
4. Neal, S., Syau, D., Nejatfard, A., Nadeau, S., Hampton, R.Y., 2020. HRD Complex Self-Remodeling Enables a Novel Route of Membrane Protein Retrotranslocation. iScience 23. doi:10.1016/j.isci.2020.101493
5. Nejatfard, A., Wauer, N., Bhaduri, S., Conn, A., Gourkanti, S., Singh, N., Kuo, T., Kandel, R., Amaro, R.E., Neal, S.E., 2021. Derlin rhomboid pseudoproteases employ substrate engagement and lipid distortion to enable the retrotranslocation of ERAD membrane substrates. Cell Rep. 37, 109840. doi:10.1016/j.celrep.2021.109840
6. Shimobayashi, M., Oppliger, W., Moes, S., Jenö, P., Hall, M.N., 2013. TORC1-regulated protein kinase Npr1 phosphorylates Orm to stimulate complex sphingolipid synthesis. Mol. Biol. Cell 24, 870–881. doi:10.1091/mbc.E12-10-0753

1st Editorial Decision **9th Sep 2022**

Thank you for sending me your detailed tentative response to the referee reports on your Dfm1/sphingolipid homeostasis study. I have now had a chance to consider your answers and plans for revising this work, and found them overall promising for addressing the key concerns raised by our three reviewers. In this light, I shall be happy to formally invite you to prepare and resubmit a manuscript modified and extended as proposed in your draft response.

UNIVERSITY OF CALIFORNIA, SAN DIEGO

BERKELEY • DAVIS • IRVINE • LOS ANGELES • MERCED • RIVERSIDE • SAN DIEGO • SAN FRANCISCO SANTA BARBARA • SANTA CRUZ

Sonya E. Neal, Ph. D.
Assistant Professor of Biological Sciences
Cell and Developmental Biology
Division of Biological Sciences
4401 Tata Hall
La Jolla, CA 92093

Mobile: (323)-303-4845
seneal@ucsd.edu

September 21, 2022

Dear Reviewers,

Please find our tentative response to the Bhaduri and Aguayo et al. paper "An ERAD-independent role for rhomboid pseudoprotease Dfm1 in mediating sphingolipid homeostasis." We have included some recent results, which addresses major concerns of all three reviewers, including plans to address issues of reportage, analysis, and discussion. We would like to thank the reviewers for their thorough, incisive, and detailed critiques of the work. Their suggestions will make the manuscript much better in many ways, from the detailed level of data presentation all the way up to the conceptual level of thinking about how derlin Dfm1 employs an ERAD-independent role for regulating sphingolipid metabolism. Below, we address in detail each point made by each reviewer, and hope that our response is satisfactory to proceed with the the next step of our story. The **black text** is verbatim from the reviewer and the **blue text** is our response. Thank you!

Sincerely yours,

Sonya Neal
Assistant Professor

Point by Point Response

Referee #1

In this manuscript, Neal and coworkers describe an involvement of the rhomboid pseudoprotease Dfm1 in the regulation of sphingolipid metabolism. This is based on the observations that (1) Dfm1 physically interacts with the SPOTS complex, (2) dfm1 deletion rescues tsc3 deletion and increases flux through the sphingolipid biosynthetic pathway, and that dfm1 and orm1 interact genetically. The second part of the manuscript investigates how Dfm1 contributes to Orm2 degradation mediated by the Dsc complex in the Golgi. It is found that Dfm1 is necessary for ER export of phosphorylated Orm2. Interestingly, this activity of Dmf1 does not require Cdc48 recruitment and is independent of ubiquitination, thus establishing a retrotranslocation independent function for Dfm1.

The authors convincingly show that Dfm1 contributes to degradation of Orm2, and that this function does not rely on its "classical" retrotranslocation and Cdc48 recruitment function but on promoting ER exit (but see point 3 below).

- “However, no convincing mechanistic model is provided how this is linked to the other observation, that dfm1 deletion in conjunction with tsc3 deletion phenocopies double deletions of orm1/2 and tsc3. My main concern is that the discrepancy between, (1) lack of Orms restores viability through increased SPOTS activity, but (2) increased stability of Orm2 in the absence of dfm1 has a similar phenotype, is not resolved. “

Thank you for pointing out this conundrum. We do agree these are important observations that needs to be reconciled. We analyzed this further by measuring PHS levels in WT or *dfm1Δ* cells either expressing phosphonull Orm2-3A or phosphomimetic Orm2-3D. As expected, in both WT and *dfm1Δ* cells, Orm2-3A (Orm2 version accumulating in the ER and not degraded by EGAD) leads to low levels of PHS whereas WT cells + Orm2-3D (phosphomimetic Orm2 constitutively degraded by EGAD) leads to significantly higher levels of PHS. Remarkably, *dfm1Δ* cells +Orm2-3D (phosphomimetic Orm2 accumulating in the ER) leads to significantly higher levels of PHS. **This finding suggests that accumulation of phosphorylated Orm2 at the ER, increases SPT activity, which phenocopies cells lacking orms1/2 where SPT activity is no longer inhibited.** We have included this new data as **Fig. 7E.**

In the future, we are interested in precisely determining how stabilized phosphorylated Orm2 at the ER leads to increased SPT activity. We have fleshed out this new line of inquiry in the discussion section.

- "Other possible mechanisms by which Dfm1 might act on sphingolipid metabolism, as suggested by variable effects on different ceramide classes, are not explored."

Thank you for pointing this out. Based on *dfm1Δorm1Δ* cells having variable effects on different ceramide classes, we hypothesize that Dfm1 modulates another enzyme(s) involved in ceramide synthesis. This exciting result opens up new studies for identifying additional role(s) for Dfm1 in contributing to sphingolipid biosynthesis. We believe this line of inquiry is outside the scope of this study since we are solely focused on deciphering Dfm1's novel role in regulating Orm2. We believe several more papers can be borne out from studying its effect on different classes of ceramides

- The fact that orm deletions are potent activators of ER stress (Han et al, PNAS, 2010) und potentially cross-talk with Dfm1 in this way, is not explored. In my view, these limitations reduce the overall impact of the study.

Thank you so much for this suggestion.

Previously, Han et al, PNAS, 2010 demonstrated that dysregulated sphingolipid metabolism through double deletion of *orm1Δorm2Δ* triggers ER stress. This was seen from growth lethality of *orm1Δorm2Δ* with treatment of protein misfolding inducing agent, tunicamycin. We have included this data (Fig. EV1C) where we see the same exacerbated growth defect with *dfm1Δorm1Δ* and not *dfm1Δorm2Δ*. These findings strengthen our claim that sphingolipid homeostasis is dysregulated in *orm1Δdfm1Δ* and that DFM1 genetically interacts with ORM1 and not ORM2. Furthermore, we are at the tip of the iceberg with understanding how Dfm1 potentially serves as a liaison between sphingolipid metabolism and ER stress. We believe further efforts in this arena are outside the scope of this particular study and is a future line of inquiry we wish to address. We have fleshed this out in the discussion section.

- "The finding of a retrotranslocation-independent mode of action for Dfm1 is certainly of interest for the membrane protein quality control field. However, this presumably chaperone-like activity of Dfm1 has also been explored by the Neal laboratory in a recent biorxiv paper (Bhaduri et al, 2022).

Thank you for pointing this out. We have supporting evidence demonstrating Dfm1's role in sphingolipid metabolism is completely new and independent from both its ERAD retrotranslocation function (Figure 5) and solubilization function (Kandel et al., 2022 & Fig. EV4C).

To test if Dfm1 is required for solubilizing Orm2, we employed our solubilization assay and showed that Dfm1 addback doesn't lead to marked increase in solubilized Orm2 since majority of Orm2 is already soluble (Fig. EV4C). Hence, Dfm1 is employing an entirely new function in sphingolipid metabolism. We believe this novel role serves as a major conceptual advancement in several fields that intersect at membrane protein quality control, membrane trafficking, and sphingolipid metabolism.

- Under what circumstances Dfm1 interacts with phosphorylated Orm2 (still part of the SPOTS complex, a rearranged spots complex, or dissociated Orm2) is not explored."

In regards to studying Dfm1's interaction with phosphorylated Orm2, we addressed this in a similar question asked (see below).

- “Other major experimental concerns:
  - Growth assays using serial dilutions of cultures on plates are used to evaluate the effect of mutations. The quality of these experiments is not very high. In many cases, no effect of dilution is discernable. “

For initial submission, we had to upload very low-quality dilution assay images to keep the file size at a minimum. We will swap in high quality images where dilution effect is discernable.

- “BioID, coIPs, and colocalization are used to establish interaction of Dfm1 with the SPOTS complex. Each of these methods has its short-coming. BioID e.g., relies on strong overexpression of Dfm1. Co-IPs of membrane proteins are tricky, especially if both proteins interrogated are relatively abundant and reside in the same membrane. Experiments in Fig. 2 would thus benefit from some control of other abundant ER proteins that should not coIP with the SPOTS complex or Dfm1.”

Thank you. We do agree this is a major short-coming of BioID and coIPs and included Sec61 as a control (see new co-IP data below).

- “Does the interaction rely on the presence of phosphorylated Orm2? This could be tested using the phosphomimetic or phosphorylation defective Orm2 mutants. Does Dfm1 only interact with the SPOTS complex through Orm2?”

We are pleased with your advice on these forward-thinking experiments. In fact, we have already begun to rigorously test this. We have included results where Dfm1 specifically interacts with phosphomimetic Orm2-3D, but not phosphonull Orm2-3A. Interestingly, with Orm2-3A, Dfm1 no longer associates with additional SPOT complex members (Orm1, Lcb1, and Lcb2). This implies, Dfm1 interacts with SPOT complex members through phosphorylated Orm2. We further confirmed this with co-IP in *orm2*Δ cell which prevents Dfm1’s association with Orm1, Lcb1, and Lcb2. This new data is in **Fig. 8A&B**.

- In Fig. 4, deletion of orm2 or double deletion of both Orms are shown to result in increased ceramide levels. Han et al. (PNAS, 2010, Figure S6) reported a decrease of ceramides under these conditions, while LCBs were increased. Please comment.

We have also noted this discrepancy. There were also two seminal studies(Breslow et al., 2010) (Shimobayashi et al., 2013), which are in agreement with our findings that lack of orms increases both LCB and ceramide levels. We believe these differences could be a result of differences in yeast growth conditions used prior to analysis (i.e-growth media, growth phase that yeasts were harvested).

- “The claim that Dfm1 acts on Orm2 independent of its Cdc48-recruitment is largely based on Fig. 5D and the comparison of stability in the presence of wt Dfm1 and the 5Ashp mutant. In this particular figure however, degradation with wt Dfm1 is considerably impaired compared to those shown in 5A and 5G. Steady state levels too do not appear to be much affected. Please comment.”

  Thank you for pointing this out. This particular replicate does not show an effect on Orm2 steady-state levels. We do have two other replicates where there is a decrease in steady-state levels with both WT and Dfm1-5Ashp mutants. We have included this in **Fig. 5D** in the revised manuscript.

  In the same figure, expression levels of the different dfm1 mutants should be shown, to exclude that defects are merely due to reduced Dfm1 levels.”

  We are grateful that you noticed this important data missing in our manuscript. We have added new data (**Fig. EV2B**) assessing steady-state levels of each mutant. We have also demonstrated expression of our mutants in our previous publications as well (Neal et al., 2018; Nejatfard et al., 2021).

Minor comments:

- p.2/3 Paragraph 2 of the introduction lacks a statement that the situation in S. cerevisiae is described here, e.g. no mention of the increased repertoire of E3 ligases involved in ERAD-M in animals, or the fact that the Asi complex only exist in fungi.

Thank you for this suggestion. We have included these very informing statements in pg. 3.

- Methods: Description of how BioID experiments in incomplete. In which buffer were the cells lysed, especially which detergent? Which strain was used? Table S2 suggests that this was done in SEN1 (which contains a DMF1 copy), whereas Fig. 1C used a dfm1 KO (although that strain is not listed in table S2).

We have added more details to the protocol in the methods section on pg. 41-42 including the lysis buffer, which is 50 mM Tris-HCl pH 7.5, 150 mM NaCl, 1.5 mM $MgCl_2$, 1 mM EDTA, 0.1% SDS, 1% NP-40, 0.4% sodium deoxycholate, and 1 mM DTT supplemented with protease inhibitors. We have included reagents table (**Appendix Table S3**), which includes the streptavidin beads used for the study. Also, thank you pointing out the strains that were not included. We have included these corresponding dfm1-null strains in **Appendix Table S2**.

- Figure legend to Fig. 1: panel enumeration is wrong. Second "D" should be "E", "E" should be "F".

We have fixed this

- Fig. 1D: Why is Cdc48 signal missing from flow-through samples? It's a very abundant protein.

We only loaded 5% of samples for flow-thru and this was a very light exposure. We added a darker exposure where you can see Cdc48 signal in flow-thru.

- Ref. 19 is incomplete.

We have included all the information in bibliography and updated reference throughout manuscript.

- Methods section lacks information on how experiments relying on Phos-tag technology were performed.

We have included a separate section in Methods section explaining how Orm2 phosphorylation status was analyzed (pg. 46).

- Several typos in Fig. 5: In 5D, it should be "Orm2-RFP" instead of "Hmg2-GFP", in 5G it should be "Orm2-RFP" instead of "Orm2-GFP".

We appreciate your diligence! We have fixed this.

- Table S4 is impossible to evaluate because column labels are not legible.

The column labels are now in larger font size. This table is now annotated as **Dataset EV1**.

Referee #2

In their manuscript, Bhaduri et al. report that the ER membrane protein Dfm1 physically interacts with the SPOTS complex which catalyzes the first step in sphingolipid synthesis. Using genetic and biochemical experiments, the authors show that dfm1Δ cells have increased ceramides, similar to orm1 and orm2 cells lacking negative regulators of sphingolipid synthesis. The authors show that Orm2 protein is stabilized in dfm1Δ cells; although Dfm1 plays a role in ERAD, the effect of dfm1Δ on Orm2 is through EGAD. In their model, the authors suggest Dfm1 promotes export of Orm2 from the ER for degradation by EGAD in post-ER compartments. The manuscript is well-written and suggests a novel function for Dfm1.To support their model in Fig. 8, the authors show genetic evidence that Orm2-3A lacking Ypk1 phosphorylation is unable to undergo EGAD, i.e. Fig 7D shows that orm2-3D does not affect growth of orm2Δ tsc3Δ cells while orm2-3A reverses the suppression seen in tsc3Δ orm2Δ cells.

Because the interpretation of the genetic result is indirect and complicated, the authors should show, more to the point, that Ypk1 is required for Orm2 interaction with Dfm1. Will Orm2 lacking Ypk1 phosphorylation sites (Orm2-3A) still interact with Dfm1? In this way, the model will be better supported and the manuscript improved. It is also of interest to determine whether Ypk1-mediated phosphorylation abrogates Orm2 association with SPOTS components while Npr1-mediated phosphorylation may not.

We are pleased to see this feedback since we immediately set out to test this. We have included the co-IP experiments in our manuscript (addressed with referee 1; **Fig. 8A-B**) demonstrating that Dfm1 specifically interacts with Orm2-3D, but not Orm2-3A. Interestingly, with Orm2-3A, Dfm1 no longer associates with Orm2 along with other SPOTS complex members (Orm1, Lcb1, and Lcb2).

We were also interested in investigating the effect of Ypk1-mediated vs. Npr1-mediated Orm2 phosphorylation on Dfm1 binding. Interestingly, in cells devoid of Npr1, Orm2 still associated with Dfm1whereas in cells devoid of Ypk1, Orm2 no longer associated with Dfm1. Interestingly, in cells lacking either Npr1 or Ypk1 or expressing Orm2-3D, Orm2 still associated with SPOT complex members implicating Orm2's phosphorylation doesn't abrogate its association with SPOTS complex. We have included this exciting new results into the revised manuscript **(Fig. 8C).**

Minor corrections:
The Han et al. reference is incompletely listed.
We have fixed this. Thank you.

At the top of page 21, one of the references to orm1Δ should be changed to orm2Δ.
Thank you for pointing this out. We have fixed this.

Referee #3 (Report for Author)

In this manuscript, Bhaduri, Aguayo and colleagues report a new function for the ERAD component Dfm1. Using unbiased protein-proximity labeling, they identify interactions with components of the SPOTS complex that functions in the first step of sphingolipid synthesis. Using genetics, they provide strong evidence that DFM1 interacts genetically with key regulators of the Lcb1/Lcb2 (SPT) enzyme and that lipid homeostasis is perturbed significantly in double mutants. Mechanistically, the authors determine that Dfm1 functions as a positive regulator of SPT by promoting the ER exit of the negative regulator Orm2. This novel function for Dfm1 requires its pseudo-rhomboid domain that interacts with ERAD substrates, but not the SHP boxes required for ERAD, supporting a non-ERAD function for Dfm1. The manuscript is very clearly written, and experiments are rigorously performed and support the conclusions. Suggestions are provided below to strengthen the conclusions and to increase the impact of the findings.

"Given the role of Dfm1 in ERAD, it is particularly important to show that the effects of Dfm1 deletion are direct and not through changes in the levels of another Dfm1-dependent ERAD substrate. "

Thank you so much for this important feedback. We do agree with the possibility of dealing with pleiotropic effects associated with Dfm1 deletion, given its role in ERAD.

Our newly acquired co-IP results demonstrates the specificity of Dfm1's effect in which it controls Orm2 ER exit by specifically binding to Ypk1-dependent phosphorylated Orm2.

Major comments:
1. To demonstrate specificity, Fig. 2A IP should include an ER membrane protein that is not detected by BioID as a negative control, especially since the detergent solubilized membranes were cleared at 14,000xg. Dfm1-GFP would not be expected to pull down Sec61, for example.

Yes, we do agree with the shortcomings of BioID experiments and plan to include a Sec61 negative control to further validate that Dfm1 interactions are specific (**Fig. 8A-C**).

2. The authors propose that Dfm1 acts directly on Orm2 through its membrane domain. To further support this model, the authors should test whether mutation of the pseudo-rhomboid disrupts the protein-protein interaction observed in Fig. 2A.

We are grateful for this suggestion since this was one of the ongoing experiments being done in the lab. Specifically, we demonstrate that rhomboid mutants we previously characterized are important for substrate targeting, are also important for Orm2 binding (**Fig. 5H**).

3. The authors demonstrate that Orm2 accumulates in the ER in dfm1D cells and is protected from degradation due to its inability to access Tul1 E3 ligase. The results in Fig 6C indicate that Orm2 is ubiquitinylated at wild-type levels in dfm1D cells indicating that it has access to Tul1. How do the authors reconcile this observation with the model that Dfm1 promotes ER exit? Could another ERAD E3 ligase act on Orm2 in dfm1D cells?

Thank you so much for pointing this out. This was failed diligence on our part. The major reason is that we have previously studied the nature of *dfm1Δ* cells and found that these cells are inherently unstable and rapidly suppressed by Hrd1(Neal et al., 2020). In other words, when DFM1 is deleted, an additional copy of Hrd1 arise via aneuploidy and can compensate for lack of Dfm1 function (Neal et al., 2020; Neal et al., 2018). We believe in this particular case, Hrd1 is taking over and ubiquitinating Orm2. We have established methods in the lab to work with this highly unstable strain (Bhaduri and Neal, 2021). We repeated the experiments with this in mind and found negligible Orm2 ubiquitination in *dfm1Δ* cells. We have added this new data in the figure (**Fig. 6C**). We also repeated the cycloheximide chase experiments to ensure we were not working with suppressed cells, and all results are aligned with the original result.

4. Lipidomic analysis of dfm1D cells suggests that DFM1 plays a relatively minor role in control of sphingolipid homeostasis. The only lipid defect observed in dfm1D cells alone is the accumulation of C18-PHS. Other defects are observed in combination with tsc3D or orm1D.

Initially, we were also surprised that dfm1Δ alone didn't have a significant effect on sphingolipid levels. However, from previous studies and our observations alone, regulators of sphingolipid homeostasis are redundant and pleiotropic in nature. For example, most characterized negative regulators of SPT, orm1 or orm2 deletion alone, also has little effect on sphingolipid homeostasis (Breslow et al., 2010; Han et al., 2010.). This is what we see with dfm1-null, where we see a major effect with double-nulls.

The most significant finding presented is that Dfm1 functions outside of ERAD (or at least SHP box function) to promote ER exit of Orm2, but mechanism is not explored. To confirm that the defect is at ER exit, the authors should test the requirement for DFM1 in an in vitro vesicle budding assay.

Thank you for pointing this out. We believe Dfm1 functions upstream of ER exit for the reason as follow:

We did not observe a general defect in COPII-mediated export in dfm1Δ cells (**Fig. EV4B**) and believe Dfm1 doesn't function directly in ER exit (also supported by no enrichment with COPII machineries in BioID experiment (**Dataset EV1**), but functions directly with the SPOTS complex in priming Orm2 for ER exit. This is supported by our new co-IP data, where we demonstrate that Dfm1 engages with phosphorylated Orm2 and not the phospho-null version of Orm2 (Orm2-3A) (addressed with referee 1 and 2; **Fig. 8A-C**).

Minor comments:
1. Why did the authors test interactions of Dfm1 with Lcb1 and Orm2 when Lcb2 and Orm1 were identified in the BioID experiment?

We have included Lcb2 and Orm1 in our co-IP experiments (**Fig. 8A-C**).

2. Please denote Lcb1 and Orm2 on the volcano plot for completeness.
We have denoted this in the volcano plot for completeness.

3. Page 8, line 1, the statement "Hence, Dfm1 binds directly to SPOTS complex members at the ER." is not supported by the data, which would require an in vitro binding experiment.

Thank you. We agree this statement is not accurate and have omitted this statement. We will soften this claim and mention that "Dfm1 associates with SPOTS complex members" instead.

References

1. Bhaduri, S., Neal, S.E., 2021. Assays for studying normal versus suppressive ERAD-associated retrotranslocation pathways in yeast. STAR Protoc. 2, 100640. doi:10.1016/j.xpro.2021.100640
2. Breslow, D.K., Collins, S.R., Bodenmiller, B., Aebersold, R., Simons, K., Shevchenko, A., Ejsing, C.S., Weissman, J.S., 2010. Orm family proteins mediate sphingolipid homeostasis. Nature 463, 1048–1053. doi:10.1038/NATURE08787
3. Han, S., Lone, M.A., Schneiter, R., Chang, A., n.d. Orm1 and Orm2 are conserved endoplasmic reticulum membrane proteins regulating lipid homeostasis and protein quality control. doi:10.1073/pnas.0911617107
4. Neal, S., Dysau, D., Nejatfard, A., Nadeau, S., and Hampton, R., n.d. HRD complex self-remodeling enables a novel route of membrane protein retrotranslocation. iScience.
5. Neal, S., Jaeger, P.A., Duttke, S.H., Benner, C.K., Glass, C., Ideker, T., Hampton, R., 2018. The Dfm1 Derlin Is Required for ERAD Retrotranslocation of Integral Membrane Proteins. Mol. Cell 69. doi:10.1016/j.molcel.2017.12.012
6. Neal, S., Syau, D., Nejatfard, A., Nadeau, S., Hampton, R.Y., 2020. HRD Complex Self-Remodeling Enables a Novel Route of Membrane Protein Retrotranslocation. iScience 23. doi:10.1016/j.isci.2020.101493
7. Nejatfard, A., Wauer, N., Bhaduri, S., Conn, A., Gourkanti, S., Singh, N., Kuo, T., Kandel, R., Amaro, R.E., Neal, S.E., 2021. Derlin rhomboid pseudoproteases employ substrate engagement and lipid distortion to enable the retrotranslocation of ERAD membrane substrates. Cell Rep. 37, 109840. doi:10.1016/j.celrep.2021.109840
8. Shimobayashi, M., Oppliger, W., Moes, S., Jenö, P., Hall, M.N., 2013. TORC1-regulated protein kinase Npr1 phosphorylates Orm to stimulate complex sphingolipid synthesis. Mol. Biol. Cell 24, 870–881. doi:10.1091/mbc.E12-10-0753

Thank you for submitting your final revised manuscript for our consideration. I am pleased to inform you that in light of the positive re-reviews by two of the original referees (copied below), we have now accepted it for publication in The EMBO Journal.

Referee #2:

I recommend the revised manuscript be accepted for publication. The authors have responded to the reviewers' comments with manuscript revision as well as new experiments. The manuscript is significantly improved.

Referee #3:

The authors have included new experiments and appropriate controls to support their conclusions and in doing so addressed all of my comments.

EMBO Press Author Checklist

Corresponding Author Name: Sonya E. Neal	
Journal Submitted to: EMBO Journal	
Manuscript Number: 112275	

USEFUL LINKS FOR COMPLETING THIS FORM
The EMBO Journal - Author Guidelines
EMBO Reports - Author Guidelines
Molecular Systems Biology - Author Guidelines
EMBO Molecular Medicine - Author Guidelines

Reporting Checklist for Life Science Articles (updated January
This checklist is adapted from Materials Design Analysis Reporting (MDAR) Checklist for Authors. MDAR establishes a minimum set of requirements in transparent reporting in the life sciences (see Statement of Task: 10.31222/osf.io/9sm4x). Please follow the journal's guidelines in preparing your
Please note that a copy of this checklist will be published alongside your article.

Abridged guidelines for figures
1. Data
The data shown in figures should satisfy the following conditions:
- the data were obtained and processed according to the field's best practice and are presented to reflect the results of the experiments in an accurate and unbiased manner.
- ideally, figure panels should include only measurements that are directly comparable to each other and obtained with the same assay.
- plots include clearly labeled error bars for independent experiments and sample sizes. Unless justified, error bars should not be shown for technical
- if n<5, the individual data points from each experiment should be plotted. Any statistical test employed should be justified.
- Source Data should be included to report the data underlying figures according to the guidelines set out in the authorship guidelines on Data

2. Captions
Each figure caption should contain the following information, for each panel where they are relevant:
- a specification of the experimental system investigated (eg cell line, species name).
- the assay(s) and method(s) used to carry out the reported observations and measurements.
- an explicit mention of the biological and chemical entity(ies) that are being measured.
- an explicit mention of the biological and chemical entity(ies) that are altered/varied/perturbed in a controlled manner.
- the exact sample size (n) for each experimental group/condition, given as a number, not a range;
- a description of the sample collection allowing the reader to understand whether the samples represent technical or biological replicates (including how many animals, litters, cultures, etc.).
- a statement of how many times the experiment shown was independently replicated in the laboratory.
- definitions of statistical methods and measures:
  - common tests, such as t-test (please specify whether paired vs. unpaired), simple χ2 tests, Wilcoxon and Mann-Whitney tests, can be unambiguously identified by name only, but more complex techniques should be described in the methods section;
  - are tests one-sided or two-sided?
  - are there adjustments for multiple comparisons?
  - exact statistical test results, e.g., P values = x but not P values < x;
  - definition of 'center values' as median or average;
  - definition of error bars as s.d. or s.e.m.

Please complete ALL of the questions below.
Select "Not Applicable" only when the requested information is not relevant for your study.

Materials

Newly Created Materials	Information included in the manuscript?	In which section is the information available? (Reagents and Tools Table, Materials and Methods, Figures, Data Availability Section)
New materials and reagents need to be available; do any restrictions apply?	Yes	Appendix Table S3

Antibodies	Information included in the manuscript?	In which section is the information available? (Reagents and Tools Table, Materials and Methods, Figures, Data Availability Section)
For **antibodies** provide the following information:<br>- Commercial antibodies: RRID (if possible) or supplier name, catalogue number and or/clone number<br>- Non-commercial: RRID or citation	Yes	Appendix Table S3

DNA and RNA sequences	Information included in the manuscript?	In which section is the information available? (Reagents and Tools Table, Materials and Methods, Figures, Data Availability Section)
**Short novel DNA or RNA including primers, probes**: provide the sequences.	Yes	Appendix Table S3

Cell materials	Information included in the manuscript?	In which section is the information available? (Reagents and Tools Table, Materials and Methods, Figures, Data Availability Section)
**Cell lines:** Provide species information, strain. Provide accession number in repository **OR** supplier name, catalog number, clone number, and/**OR** RRID.	Not Applicable	Yeast strains and plasmid strains are in Appendix Table S2
**Primary cultures:** Provide species, strain, sex of origin, genetic modification status.	Not Applicable	
Report if the cell lines were recently **authenticated** (e.g., by STR profiling) and tested for mycoplasma contamination.	Not Applicable	

Experimental animals	Information included in the manuscript?	In which section is the information available? (Reagents and Tools Table, Materials and Methods, Figures, Data Availability Section)
**Laboratory animals or Model organisms:** Provide species, strain, sex, age, genetic modification status. Provide accession number in repository **OR** supplier name, catalog number, clone number, **OR** RRID.	Not Applicable	
**Animal observed in or captured from the field:** Provide species, sex, and age where possible.	Not Applicable	
Please detail **housing and husbandry conditions**.	Not Applicable	

Plants and microbes	Information included in the manuscript?	In which section is the information available? (Reagents and Tools Table, Materials and Methods, Figures, Data Availability Section)
**Plants:** provide species and strain, ecotype and cultivar where relevant, unique accession number if available, and source (including location for collected wild specimens).	Not Applicable	
**Microbes:** provide species and strain, unique accession number if available, and source.	Yes	Appendix Table S2

Human research participants	Information included in the manuscript?	In which section is the information available? (Reagents and Tools Table, Materials and Methods, Figures, Data Availability Section)
If collected and within the bounds of privacy constraints report on age, sex and gender or ethnicity for all study participants.	Not Applicable	

Core facilities	Information included in the manuscript?	In which section is the information available? (Reagents and Tools Table, Materials and Methods, Figures, Data Availability Section)
If your work benefited from core facilities, was their service mentioned in the acknowledgments section?	Yes	Acknowledgements section

Design

Study protocol	Information included in the manuscript?	In which section is the information available? (Reagents and Tools Table, Materials and Methods, Figures, Data Availability Section)
If study protocol has been **pre-registered, provide DOI in the manuscript**. For clinical trials, provide the trial registration number **OR** cite DOI.	Not Applicable	
Report the **clinical trial registration number** (at ClinicalTrials.gov or equivalent), where applicable.	Not Applicable	

Laboratory protocol	Information included in the manuscript?	In which section is the information available? (Reagents and Tools Table, Materials and Methods, Figures, Data Availability Section)
Provide DOI OR other citation details if **external detailed step-by-step protocols** are available.	Not Applicable	

Experimental study design and statistics	Information included in the manuscript?	In which section is the information available? (Reagents and Tools Table, Materials and Methods, Figures, Data Availability Section)
Include a statement about **sample size** estimate even if no statistical methods were used.	Not Applicable	
Were any steps taken to minimize the effects of subjective bias when allocating animals/samples to treatment (e.g. **randomization procedure**)? If yes, have they been described?	Not Applicable	
Include a statement about **blinding** even if no blinding was done.	Not Applicable	
Describe **inclusion/exclusion criteria** if samples or animals were excluded from the analysis. Were the criteria pre-established?<br><br>If sample or data points were omitted from analysis, report if this was due to attrition or intentional exclusion and provide justification.	Not Applicable	
For every figure, are **statistical tests** justified as appropriate? Do the data meet the assumptions of the tests (e.g., normal distribution)? Describe any methods used to assess it. Is there an estimate of variation within each group of data? Is the variance similar between the groups that are being statistically compared?	Yes	Figure legends

Sample definition and in-laboratory replication	Information included in the manuscript?	In which section is the information available? (Reagents and Tools Table, Materials and Methods, Figures, Data Availability Section)
In the figure legends: state number of times the experiment was **replicated** in laboratory.	Yes	Figure Legends
In the figure legends: define whether data describe **technical or biological replicates**.	Yes	Figure Legends

Ethics

Ethics	Information included in the manuscript?	In which section is the information available? (Reagents and Tools Table, Materials and Methods, Figures, Data Availability Section)
Studies involving **human participants**: State details of **authority granting ethics approval** (IRB or equivalent committee(s), provide reference number for approval.	Not Applicable	
Studies involving **human participants**: Include a statement confirming that **informed consent** was obtained from all subjects and that the experiments conformed to the principles set out in the WMA Declaration of Helsinki and the Department of Health and Human Services Belmont Report.	Not Applicable	
Studies involving **human participants:** For publication of **patient photos**, include a statement confirming that consent to publish was obtained.	Not Applicable	
Studies involving experimental **animals**: State details of **authority granting ethics approva**l (IRB or equivalent committee(s), provide reference number for approval. Include a statement of compliance with ethical regulations.	Not Applicable	
Studies involving **specimen and field samples:** State if relevant **permits** obtained, provide details of authority approving study; if none were required, explain why.	Not Applicable	

Dual Use Research of Concern (DURC)	Information included in the manuscript?	In which section is the information available? (Reagents and Tools Table, Materials and Methods, Figures, Data Availability Section)
Could your study fall under dual use research restrictions? Please check biosecurity documents and list of **select agents and toxins** (CDC): https://www.selectagents.gov/sat/list.htm	Not Applicable	
If you used a select agent, is the security level of the lab appropriate and reported in the manuscript?	Not Applicable	
If a study is subject to dual use research of concern regulations, is the name of the **authority granting approval and reference number** for the regulatory approval provided in the manuscript?	Not Applicable	

Reporting

The MDAR framework recommends adoption of discipline-specific guidelines, established and endorsed through community initiatives. Journals have their own policy about requiring specific guidelines and recommendations to complement MDAR.

Adherence to community standards	Information included in the manuscript?	In which section is the information available? (Reagents and Tools Table, Materials and Methods, Figures, Data Availability Section)
State if relevant guidelines or checklists (e.g., **ICMJE, MIBBI, ARRIVE, PRISMA**) have been followed or provided.	Not Applicable	
For **tumor marker prognostic studies,** we recommend that you follow the **REMARK** reporting guidelines (see link list at top right). See author guidelines, under 'Reporting Guidelines'. Please confirm you have followed these guidelines.	Not Applicable	
For **phase II and III randomized controlled trials**, please refer to the **CONSORT** flow diagram (see link list at top right) and submit the CONSORT checklist (see link list at top right) with your submission. See author guidelines, under 'Reporting Guidelines'. Please confirm you have submitted this list.	Not Applicable	

Data Availability

Data availability	Information included in the manuscript?	In which section is the information available? (Reagents and Tools Table, Materials and Methods, Figures, Data Availability Section)
Have **primary datasets** been deposited according to the journal's guidelines (see 'Data Deposition' section) and the respective accession numbers provided in the Data Availability Section?	Yes	Data availability section
Were **human clinical and genomic datasets** deposited in a public access-controlled repository in accordance to ethical obligations to the patients and to the applicable consent agreement?	Not Applicable	
Are **computational models** that are central and integral to a study available without restrictions in a machine-readable form? Were the relevant accession numbers or links provided?	Not Applicable	
If publicly available data were reused, provide the respective **data citations in the reference list**.	Not Applicable	
